# Efficacy and safety outcomes reported in human leptospirosis studies to inform the development of a core outcome and core outcome measurement set: A systematic review

**DOI:** 10.1371/journal.pntd.0013651

**Published:** 2026-07-13

**Authors:** Nathaniel Lee, Ines Corcuera Hotz, Chris Smith, Robin Bailey, Koya Ariyoshi, Tansy Edwards

**Affiliations:** 1 Faculty of Epidemiology and Population Health, London School of Hygiene & Tropical Medicine, London, United Kingdom; 2 School of Tropical Medicine and Global Health, Nagasaki University, Japan; 3 Emergency Department, Royal Melbourne Hospital, Melbourne, Australia; 4 Faculty of Infectious and Tropical Diseases, London School of Hygiene & Tropical Medicine, London, United Kingdom; 5 Department of Clinical Medicine, Institute of Tropical Medicine (NEKKEN), Nagasaki University, Nagasaki, Japan; 6 Global Health Trials Unit, Department of Clinical Sciences, Liverpool School of Tropical Medicine, Liverpool, United Kingdom; Faculty of Medicine and Health Sciences, Universiti Putra Malaysia, MALAYSIA

## Abstract

**Background:**

Leptospirosis is a neglected bacterial zoonotic disease of global health importance, disproportionately affecting marginalised communities in tropical and subtropical settings. Current clinical trial evidence for leptospirosis treatments remains limited by heterogeneous outcome reporting and trial design. This systematic review represents the first comprehensive synthesis of outcomes, outcome measures, and treatments reported across human leptospirosis research to inform the development of a core outcome and outcome measurement set.

**Methods:**

This systematic review was registered (COMET and PROSPERO: CRD42023397461) and conducted in accordance with a pre-published protocol. Global databases were searched for studies published on human leptospirosis without language or geographical restriction. Screening and full-text review were performed independently by two reviewers. Study characteristics, population, interventions, and reported outcomes, definitions and measurement tools were extracted. Quality and risk of bias assessments were performed. Outcomes were analysed descriptively and grouped thematically into domains.

**Results:**

This review included 298 studies from 63 countries. There were 172 unique outcome types extracted and categorized into 23 domains. The clinical/physiological core area was the most frequently reported, with the renal and urinary domain comprising a quarter of all studies. Individual outcomes that predominated included mortality (165/298 studies), discharge from clinical services (79/298), and days of hospitalisation (77/298). Patient focused and harms outcomes were under-reported across studies. Quality assessments highlighted considerable study bias which did not improve with increasing study rigour.

There were 92 outcome measures extracted and categorized into 13 thematic areas and of note was the inconsistency in types of measures, absence of patient-reported outcomes, and need for standardised and validated outcome definitions. Data captured on treatments highlighted the variability in clinical management practices worldwide.

**Discussion/Conclusion:**

This review highlights the heterogeneity in outcome reporting, outcome measures, and treatments across human leptospirosis research. Establishing a consensus on core outcomes and core outcome measurements that are clinically relevant, patient-centred, and robust is needed to better support evidence-based guidelines.

## Author summary

Human leptospirosis is the most common bacterial infection that spreads from animals to people worldwide. Severe disease can lead to organ damage and death. The current clinical trial evidence supporting the treatment of Leptospirosis is weak and inconclusive. To improve this, standardised outcomes and outcome measures are required to enable better comparability between studies. This was the first systematic review done to summarize the outcomes previously used in leptospirosis studies as well as the way they were measured. The objective was to construct a group of crucial outcomes that would be measured in any future leptospirosis interventional study, which is known as a core outcome set.

We reviewed 298 studies and found 172 different types of outcomes. These individual outcomes were grouped, and those in the category of body functions and clinical signs were most reported (222 studies). Only 34 studies reported outcomes describing treatment harms, and even fewer (6 studies) reported quality of life outcomes. No studies included patient-reported outcomes. The ways outcomes were measured varied widely, with no consistent approach. Treatment strategies for leptospirosis also differed greatly across countries.

This systematic review supports the need for a core outcome set for future leptospirosis trials that includes well-defined outcomes that are both practical to use and measure. This would improve data nd research quality and encourage the use of patient-centred outcomes.

## Introduction

Leptospirosis is a globally distributed zoonotic bacterial infection, with an estimated attributable 1.03 million new cases and 58,900 deaths worldwide per year [1]. Those living in marginalized communities, often in tropical or subtropical urban settings with limited physical infrastructure, face the greatest burden of disease [2]. The point estimate for global productivity loss due to leptospirosis is USD 29.3 billion, with upper ranges of the estimate as high as USD 52.3 billion [3]. There is a broad spectrum of clinical manifestations ranging from a flu-like illness to multi-organ failure [4]. Severe disease manifests as a variety of end-organ complications including a reversible hepatic injury, meningoencephalitis, myocarditis, renal failure, and pulmonary haemorrhage [5]. Disease morbidity is most attributable to renal and pulmonary complications [6].

Methodological inconsistencies across the limited number of existing trials constrains the current evidence base for the management of leptospirosis. A 2024 Cochrane review of antibiotic treatment included nine randomised trials, with outcome measures of interest including mortality, serious adverse events, quality of life, and length of hospital stay. The review concluded there was insufficient evidence to advocate for or against the use of antimicrobials due to poor methodological quality or underpowered studies [7]. Similar conclusions were drawn from the Cochrane reviews of antibiotic prophylaxis as well as corticosteroid treatment for leptospirosis [8,9].

A core outcome set (COS) is a minimum set of outcomes that should be measured and reported across all studies for a particular disease or condition [10]. Developing a COS for a disease or condition creates a robust tool to measure the effect of an intervention, enhancing the ability to make meaningful comparisons between trials, observational cohorts, and surveillance studies [11]. There has not yet been a COS established for use in human leptospirosis studies. Establishing a COS is a multi-step process, of which this study represents the first stage summarizing the existing outcomes previously investigated across all human leptospirosis studies. Beyond improving trial quality and comparability, standardisation of outcomes using a COS would enable more reliable synthesis of evidence to inform clinical guidelines, result in targeted treatment resource allocation in endemic settings, and ensure that treatment evaluation is aligned with outcomes meaningful to patients and health system.

The aim of this review is to establish existing knowledge about outcomes and treatments previously reported in quantitative and qualitative human leptospirosis studies to inform the development of a core outcome set and core outcome measure set.

## Methods

### Ethics statement

Ethics approval was received by London School of Hygiene and Tropical Medicine Observational Research Ethics Committee (LSHTM Ethics Ref 29935). Formal consent was not required as publications reviewed were in the public domain.

The methodology for this systematic review is provided in detail in a published protocol describing the process for the core outcome set development for leptospirosis trials (COS-LEP) project [12]. The review was registered on the COMET initiative and PROSPERO database (CRD42023397461). Preferred Reporting Items for Systematic reviews and Meta-analysis (PRISMA) checklists are provided in **S1 Appendix** **and** **S2 Appendix**.

### Study search strategy and selection

A search was conducted of the following databases: the Cochrane Database of Systematic Reviews, Cochrane Central Register of Controlled Trials, MEDLINE, EMBASE, LILACS, Web of Science Core Collection, Clinicaltrials.gov, and OpenSIGLE. Detailed search terms by database and all dates of searches are described in **S3 Appendix**. For each identified study, forward and backward reference searching using Google Scholar was used to identify additional publications for inclusion.

Any study focusing on human leptospirosis that reported patient outcomes across all age groups globally was included. To maximise identification of all previously reported outcomes and ensure broad international representation while minimising the potential for overlooking clinically significant outcomes, all study design types, both descriptive and analytical, were eligible for inclusion. The incorporation of descriptive studies, including case reports and case series, was intentional as, given the neglected status of leptospirosis and likely limitations of existing literature, inclusion of descriptive study designs was considered essential to ensure capture of all previously reported outcomes. This is in line with established Core Outcome Measures in Effectiveness Trials (COMET) Initiative recommendations [10]. Non peer-reviewed studies such as protocols or registry entries (trial or observational) were included as long as planned outcomes were reported. Full manuscripts were prioritised. Abstract-only publications were included if we were unable to obtain the full publication from the publisher or manuscript author, and as a minimum the abstract included outcomes reported by the study [10].

The search was not limited by language, and translations were done either by the reviewing authors or using machine translation (Google Translate). Further guidance and requirements were followed as specified by the COMET Initiative and PRISMA statement [10,13].

### Data extraction

We planned to assess studies in batches chronologically by descending years in 5-year frames until saturation in outcome types was achieved. However, all screened studies were included as we did not reach a stage of outcome saturation.

Studies were screened using the Covidence systematic review software (https://www.covidence.org/). Title and abstract review, followed by full text review, was performed by two reviewers (NL and ICH) working independently. A third reviewer (TE) was planned to be consulted if there was disagreement between the first two reviewers, but this did not occur. Agreement at these stages was calculated using Cohen’s coefficient kappa to demonstrate the level of agreement between reviewers [14].

The following data was extracted during the full text review (**S4 Appendix**): study information including year of publication, institution, and location study was conducted, study design, inclusion/exclusion criteria verbatim; study population – demographics, epidemiological risk factors; interventions used and details; outcomes; outcome measurements, instruments, and/or definitions provided by the authors for each outcome; and the study conclusion verbatim.

### Quality assessment and outcome reporting bias

Quality assessment was not performed with the intent to exclude studies from the review, but rather to provide an objective analysis of the study quality of leptospirosis literature reporting outcomes. The primary author (NL) assessed the quality of included studies using the QualSyst tool (**S5 Appendix**), developed for quality assessment of a variety of study designs [15]. The study was assessed using a template with scoring categories of Yes (2 points), Partial (1 point), No (0 points), and N/A. Summary scores are reported as the percentage proportion of the total possible score (i.e., exclusive of N/A scores), with a larger proportion indicative of a higher level of quality.

Selection of outcomes that are reported in any study is often subject to bias, usually based on the result that is being reported. The Outcome Reporting Bias in Trials (ORBIT) tool has been designed to assess for this selective reporting bias (**S5 Appendix**) [16]. Studies included in this systematic review were assessed using the ORBIT tool by the primary author (NL) to quantify the amount of selective outcome reporting provided they were observational studies (cross-sectional, cohort, or case-control), trials, or meta-analysis. The goal was to assess and demonstrate the risk of reporting bias in included studies, rather than as a basis for study exclusion.

### Data synthesis

All summary statistics were performed in Stata (Stata/IC 15.1). Data describing study design, study population, treatments, and quality/risk of bias assessments were summarized with descriptive statistics. A narrative synthesis was conducted of reported outcomes, which were then gathered based on terminology into group names and then further grouped into domains [10]. As outcome group names and domains were dependent on actual outcomes identified, these were not defined *a priori*. Outcome domains were initially recorded as reported. Thematically similar domains were combined and mapped to the outcome framework proposed by the COMET Initiative [10]. Outcome groupings and COMET domain mapping was validated by consensus amongst all authors. We considered presenting outcomes by subgroup if there were defined populations targeted by a particular outcome, but this did not apply for this review. Any disagreements regarding outcome classification would have been resolved first by discussion between study authors, and where necessary, at a project steering committee meeting, but this did not apply for this review. Reported outcomes measures were summarized first by grouping thematically, and then categorizing based on the type of outcome. Outcomes were plotted on a sunburst chart to visually illustrate their reporting frequency. Similarly, reported antibiotic treatment types were plotted to a treemap chart.

## Results

**Fig 1:**
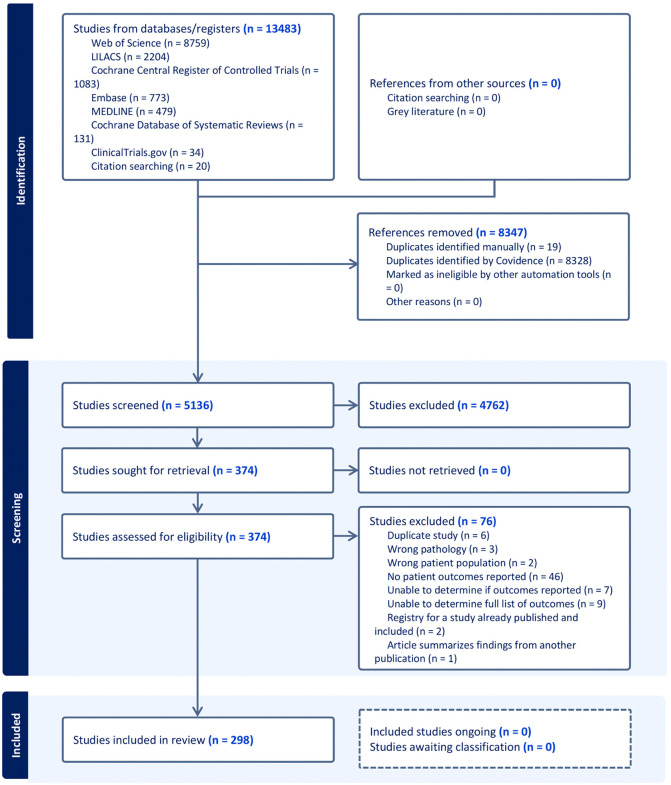
PRISMA flowchart demonstrating database and register searches, screened studies, and included studies.

The full study search returned a total of 13,483 results. The search was up-to-date as of 28/05/2025. After an automatic, and then further manual removal of duplicate studies, there remained 5,136 studies to be screened. Of these, 374 were selected from title and abstract review to go on to full-text review and 76 studies were subsequently excluded at this stage (see **Fig 1**), with 298 studies being included for data extraction. A complete reference list of the included 298 studies is included in **S6 Appendix**. The complete cleaned summary dataset is available at DOI:10.5281/zenodo.17148991.

### General characteristics

**Fig 2:**
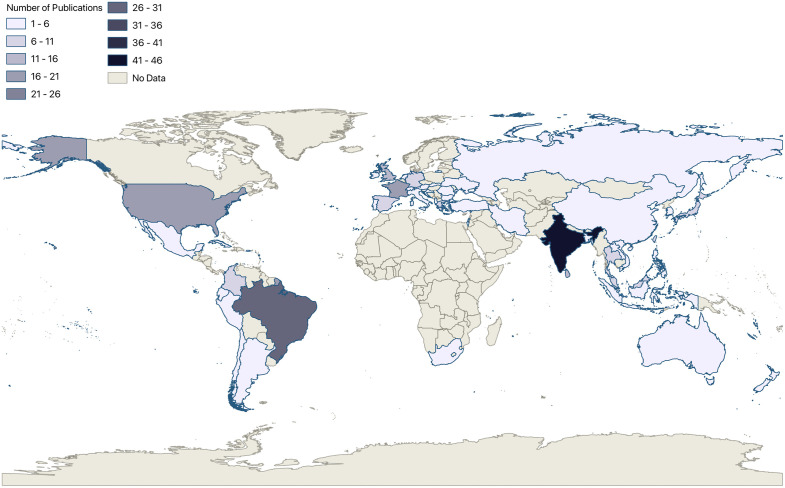
Number of publications reporting Leptospirosis treatment outcomes by country. Created using QGIS 3.34.11-Prizren and Natural Earth (naturalearthdata.com).

Consistent with the global distribution of disease, publications from 63 countries across most continents were included (**Fig 2**). The country with the most publications was India (14.4%; 43/298) followed by Brazil (9.7%; 29/298). Descriptive studies comprised 58.4% (174/298) of included studies, of which case reports formed the majority (81.0%; 141/174) of the group. Observational studies were the second most frequent included study type, of which almost half were of retrospective cohort design (47.1%; 33/70). Interventional studies were the third most frequent study type, with randomised controlled trials being the most frequently used design within this category (79.2%; 19/24).

Most included studies were published in the last 15 years (67.8%; 202/298). Prior to 2000, the Latin American region produced the largest proportion of included studies (~30%; **S7 Appendix**). The most common study design was case reports (37%) followed by interventional studies (33%). In subsequent periods, South Asia became the leading region, contributing 21% of included studies between 2000 – 2019, and 25% from 2020 onwards. Case reports remained the most common study design followed by observational studies (respectively 53% and 23% between 2000 – 2019, followed by 35% and 29% from 2020 onwards).

There were 25,876 participants described across all studies exclusive of systematic reviews (to avoid duplication of counts), protocols, registries, modelling, and economic evaluations (Table 1). In studies reporting population descriptions (38.1%; 114/298), half described endemic settings assessing participants drawn from the general population (50.0%; 57/114).

**Table 1:** Characteristics of included studies.

Total number of studies = 298	Number of studies N (%)		Number of studies N (%)
Decade of publication		Population description	
1956 – 1959	2 (0.7%)	Total number of participants (n)*	25,876
1960 – 1969	0	Endemic setting	
1970 – 1979	0	General population	57 (19.1%)
1980 – 1989	11 (3.7%)	Hospitalized population	37 (12.4%)
1990 – 1999	14 (4.7%)	Agricultural population	3 (1.0%)
2000 – 2009	69 (23.2%)	Elderly population	1 (0.3%)
2010 – 2019	125 (42.0%)	Military population	4 (1.3%)
2020 – 2025	77 (25.8%)	Non-endemic setting	
		General population	8 (2.7%)
**Publication language**		At-risk occupation population	4 (1.3%)
English	262 (87.9%)	Description not applicable	129 (43.3%)
Spanish	19 (6.4%)	Not reported	55 (18.5%)
French	6 (2.0%)		
German	3 (1.0%)		
Portuguese	2 (0.7%)	Inclusion criteria given** (n = 142)	
Romanian	2 (0.7%)	Interventional studies (n = 24)	22 (91.7%)
Chinese	1 (0.3%)	Observational studies (n = 70)	63 (90.0%)
Dutch	1 (0.3%)	Systematic reviews (n = 22)	17 (77.3%)
Russian	1 (0.3%)	Surveillance (n = 6)	6 (100%)
Turkish	1 (0.3%)	Modelling (n = 1)	1 (33.3%)
		Trial/study registry (n = 15)	15 (100.0%)
**Study design**		Trial/study protocol (n = 2)	2 (100.0%)
Interventional			
Randomised controlled clinical trial	19 (6.4%)	Exclusion criteria given**(n = 142)	
Non-randomised controlled clinical trial	3 (1.0%)	Interventional studies (n = 24)	14 (58.3%)
Non-randomised non-controlled clinical trial	2 (0.7%)	Observational studies (n = 70)	9 (12.9%)
Trial protocol	1 (0.3%)	Systematic studies (n = 22)	7 (31.8%)
Trial registry	6 (2.0%)	Surveillance (n = 5)	0
Observational		Modelling (n = 2)	0
Cross sectional	8 (2.7%)	Trial/study registry (n = 15)	12 (80.0%)
Case-control	7 (2.4%)	Trial/study protocol (n = 2)	1 (50.0%)
Cohort (Prospective)	22 (7.4%)		
Cohort (Retrospective)	33 (11.1%)		
Study Registry	9 (3.0%)		
Review			
Literature review	2 (0.7%)		
Systematic review (narrative only)	11 (3.7%)		
Systematic review with meta-analysis	9 (3.0%)		
Protocol	1 (0.3%)		
Surveillance study	6 (2.0%)		
Modelling	3 (1.0%)		
Economic evaluation	1 (0.3%)		
Case report	141 (47.3%)		
Case series	14 (4.7%)		

*Excluding participants described in systematic reviews, protocols, registries, modelling, and economic evaluations **Not including economic evaluations, case reports, and case series

### Reported outcomes

**Fig 3:**
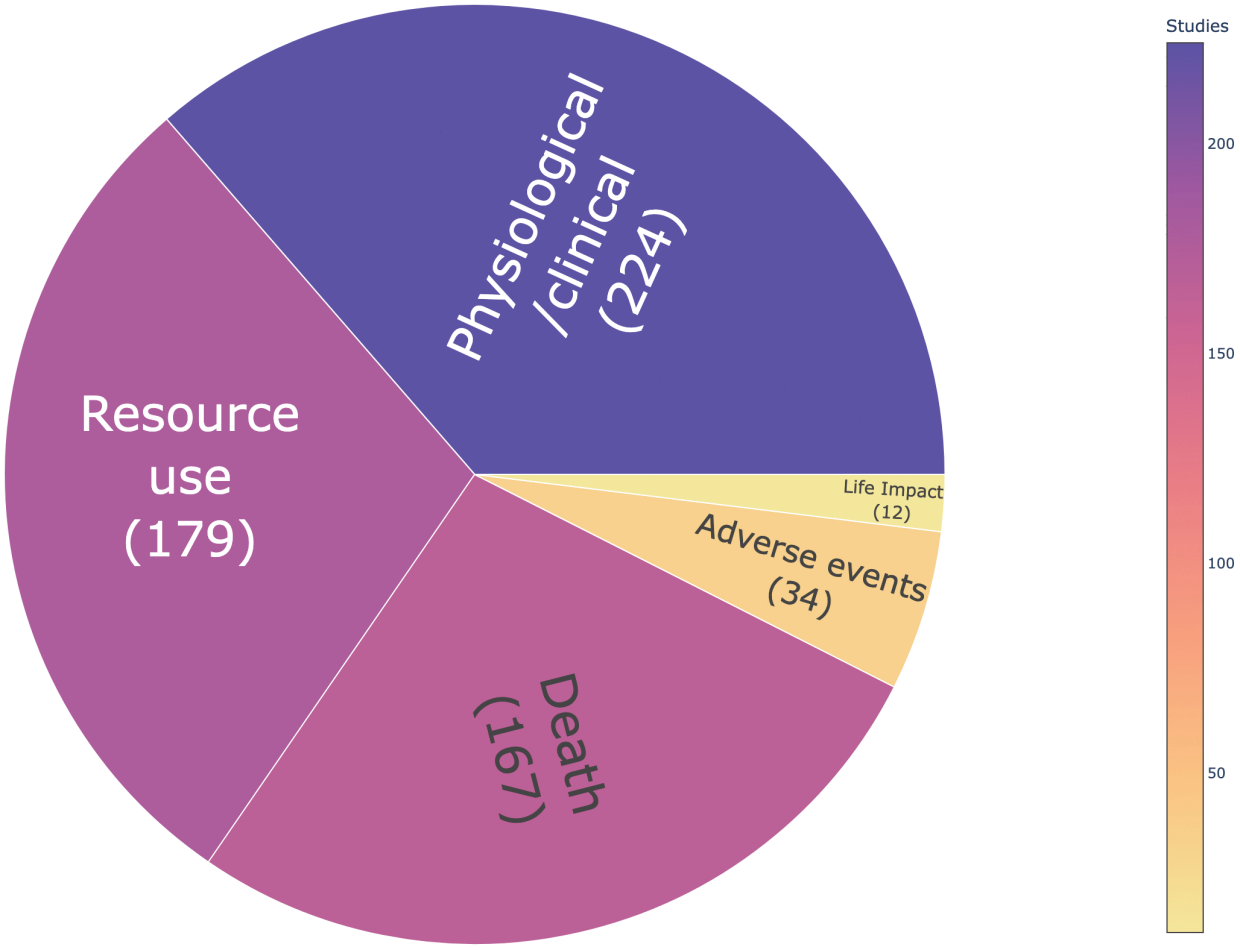
Sunburst chart demonstrating discrete outcomes and frequency of reporting by number of studies across all COMET core areas. Made using plotly on Python 3.13.

There were 172 discrete outcomes reported across all studies (**Fig 3** and **S6 Appendix**). The median number of outcomes per study was 3 (range 1–12). There were 55.0% (164/298) studies reporting 1–3 outcomes, 32.9% (98/298) reporting 4–6 outcomes, 7.7% (23/298) reporting 7–9 outcomes, 3.7% (11/298) reporting 10–12 outcomes, and 0.7% (2/298) reporting 13–15 outcomes. Included outcomes were categorized across all five COMET taxonomical core areas.

**Fig 4:**
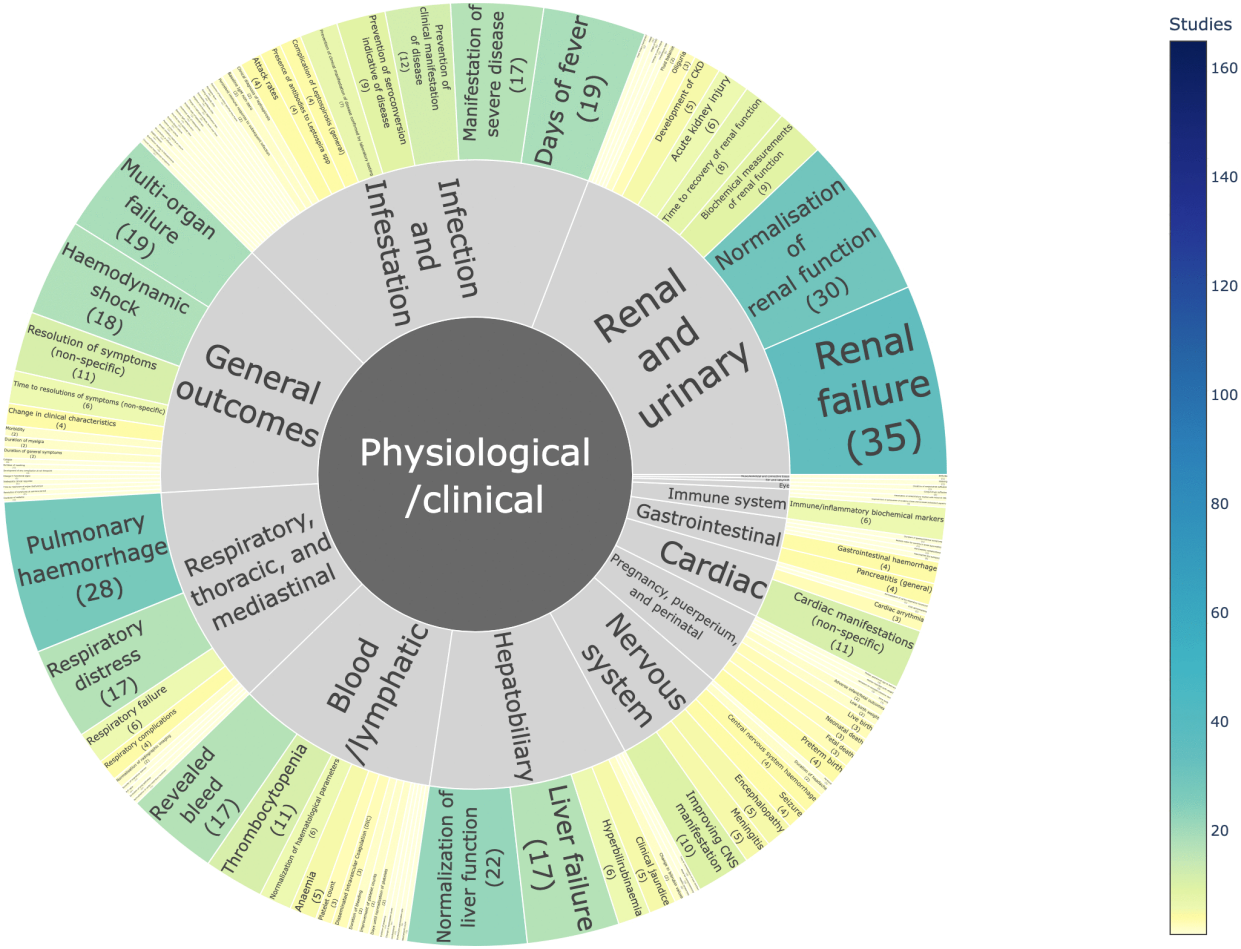
Sunburst chart demonstrating discrete outcomes and frequency of reporting by number of studies across COMET core area of physiological/clinical. Made using plotly on Python 3.13.

**Fig 5:**
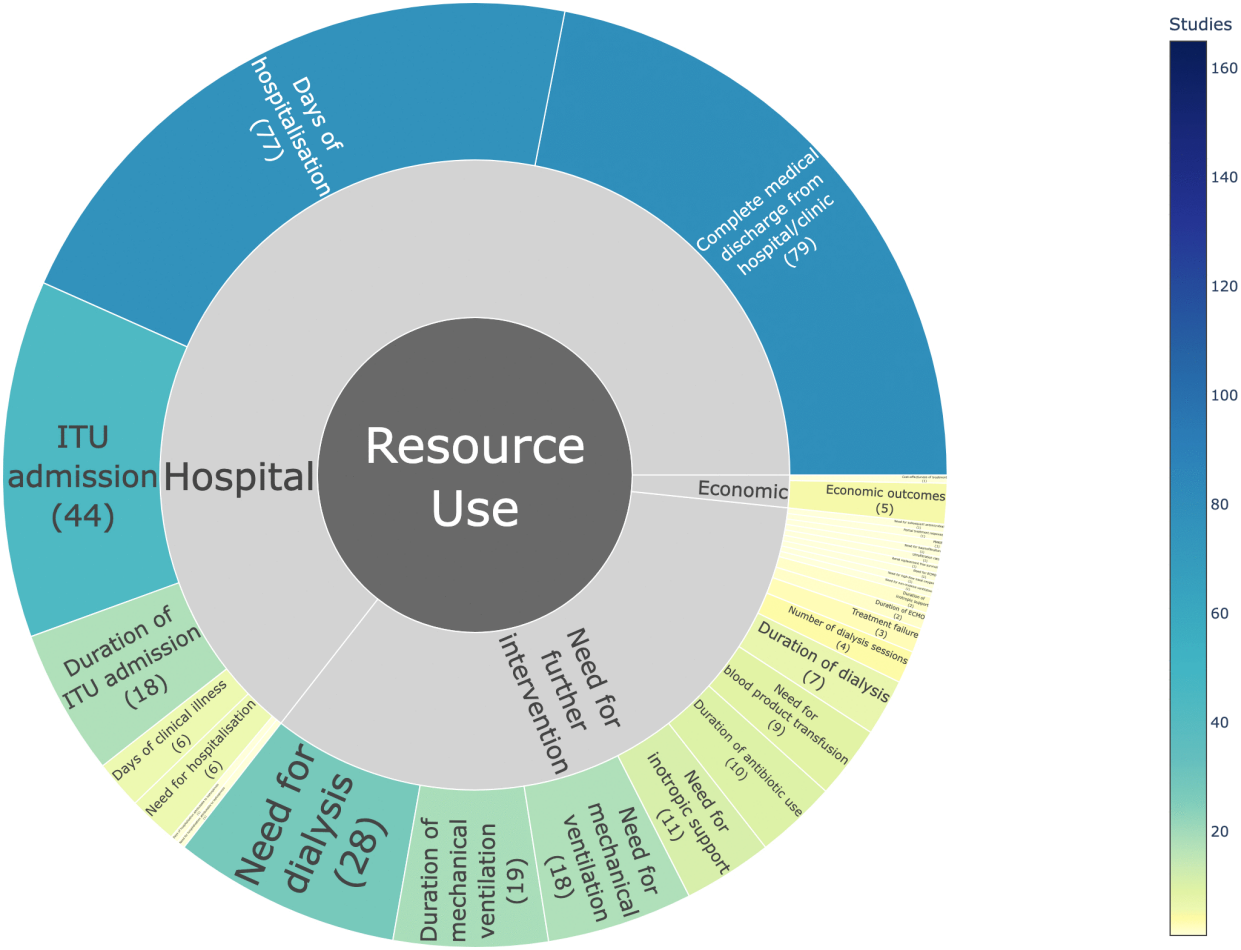
Sunburst chart demonstrating discrete outcomes and frequency of reporting by number of studies across COMET core area of resource use. Made using plotly on Python 3.13.

The COMET core area of physiological/clinical was the most frequently reported core area (**Fig 4**; 74.5%; 222/298), with 14 COMET domains included in this core area. The number of outcome types per domain ranged from 1 – 28. All included outcomes were classified as benefit associated except for one – “Association of inflammatory marker with onset of JHR”. The COMET domain of renal and urinary included 13 outcome types reported in 26.5% (79/298) of studies. The COMET domain of general outcomes had 15 outcome types reported in 20.5% (61/298) of studies. The COMET domain of infection and infestation had 28 outcome types reported in 20.1% (60/298) of studies. The COMET domain of hepatobiliary included 8 outcome types reported in 16.8% (50/298) of studies. The COMET domain of respiratory, thoracic, and mediastinal included 9 outcome types reported in 16.4% (49/298) of studies. The COMET domain of blood and lymphatic system included 14 outcome types reported in 14.4% (43/298) of studies. The COMET domain of nervous system included 7 outcome types reported in 9.1% (27/298) of studies. The COMET domain of cardiac included 4 outcome types reported in 5.0% (15/298) of studies. The COMET domain of gastrointestinal included 6 outcome types reported in 3.0% (9/298) of studies. The COMET domain of immune system included 3 outcome types reported in 2.4% (7/298) of studies. The COMET domain of pregnancy, puerperium, and perinatal included 10 outcome types reported in 1.7% (5/298) of studies. The COMET domain of eye included 2 outcome types reported in 0.7% (2/298) of studies. The COMET domains of ear as well as musculoskeletal/connective tissues both included one outcome type reported in 0.3% (1/298) of studies respectively. In studies published prior to 2000, the most commonly reported outcomes came from the COMET renal domain (37.0%; **S7 Appendix**). This pattern persisted between 2000 – 2019 (28.4%), however since 2020 infection outcomes have become the most frequently reported (28.6%),

The COMET core area of resource use was the second most frequently reported core area (**Fig 5**; 59.4%; 177/298). There were three COMET domains included in this core area. The number of outcome types per domain ranged from 2 – 20. All included outcomes were classified as benefit associated. The COMET domain of hospital included 8 outcome types reported in 53.7% (160/298) of studies. The COMET domain of need for further intervention included 20 outcome types reported in 24.8% (74/298) of studies. The COMET domain of economic included 2 outcome types reported in 2.0% (6/298) of studies. Although the COMET domain of hospital outcomes remains consistently reported on, the proportion of outcomes describing the need for further intervention became increasingly more commonly reported in recent years – 24.7% after 2020 compared to 7.4% prior to 2000 (**S7 Appendix**).

**Fig 6:**
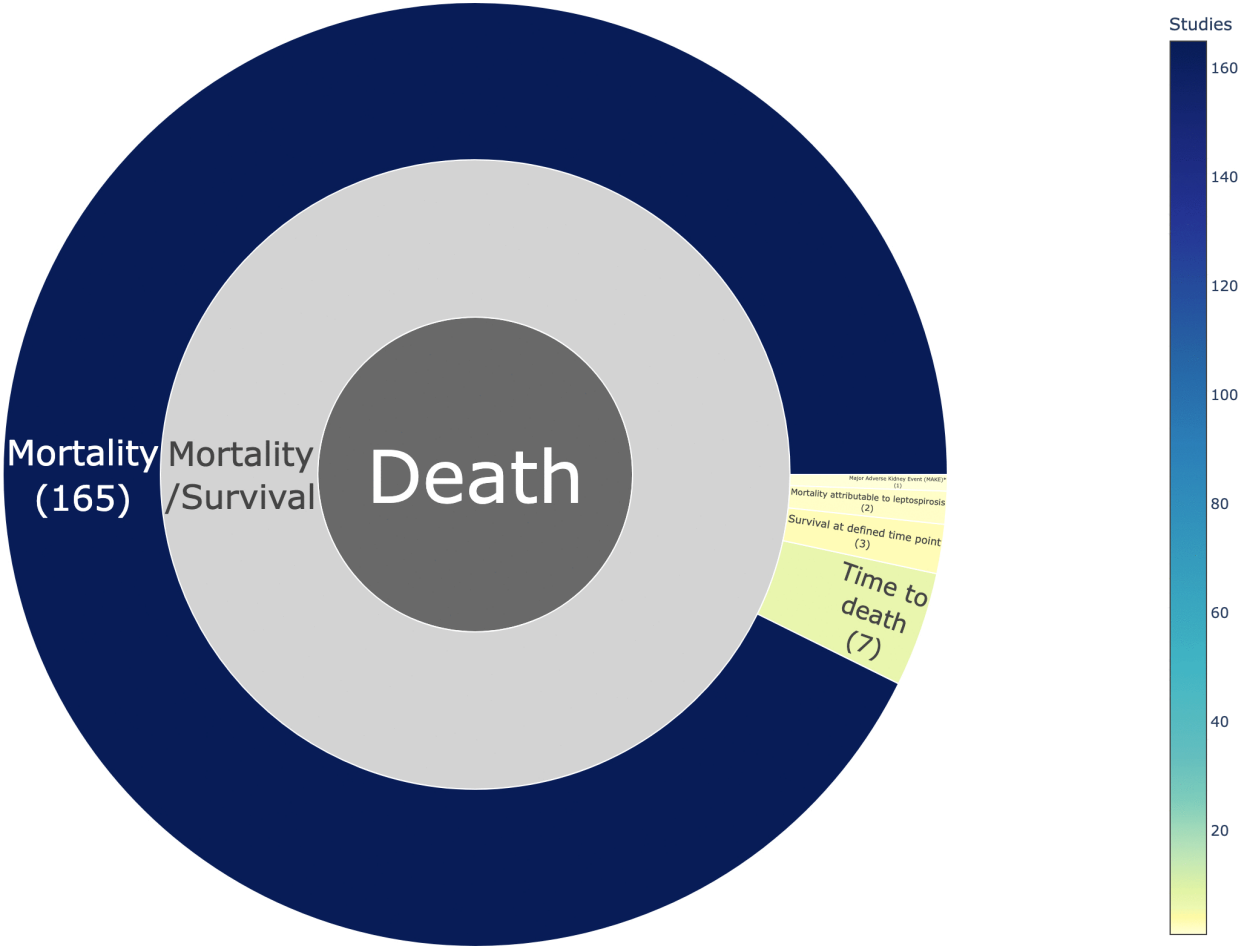
Sunburst chart demonstrating discrete outcomes and frequency of reporting by number of studies across COMET core area of death. Made using plotly on Python 3.13.

The COMET core area of death was the third most frequently reported core area (**Fig 6**; 56.0%; 167/298), with 5 outcome types included in the sole domain which were all classified as benefit associated. The proportion of studies reporting death has remained consistently around 55% throughout the included published years (**S7 Appendix**).

**Fig 7:**
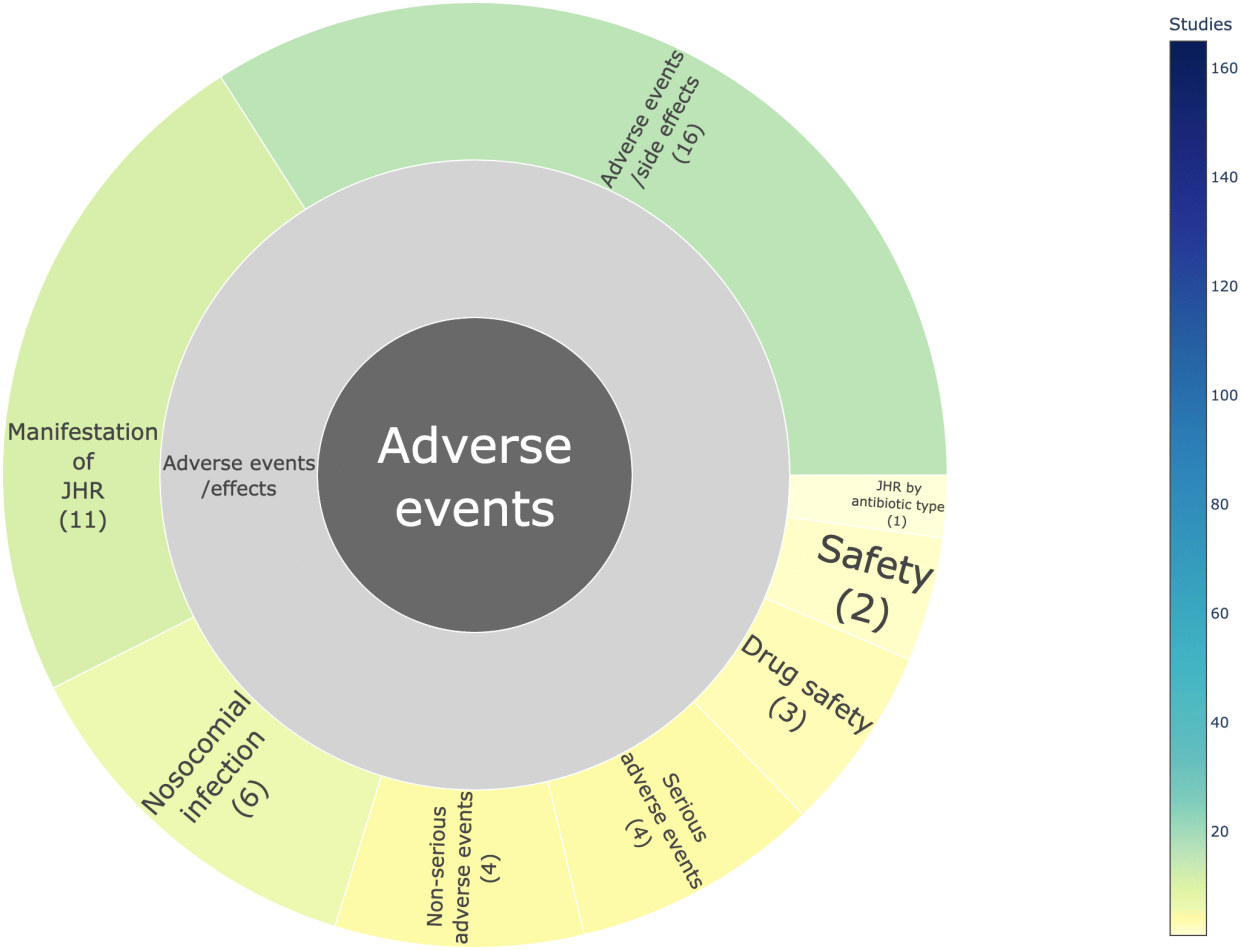
Sunburst chart demonstrating discrete outcomes and frequency of reporting by number of studies across COMET core area of adverse events. Made using plotly on Python 3.13.

The COMET core area of adverse events was the fourth most frequently reported core area (**Fig 7**; 11.4%; 34/298), with 8 outcome types included in the sole domain which were all classified as harm associated. The proportion of studies reporting adverse events has remained consistently between 10% - 15% throughout the included published years (**S7 Appendix**).

**Fig 8:**
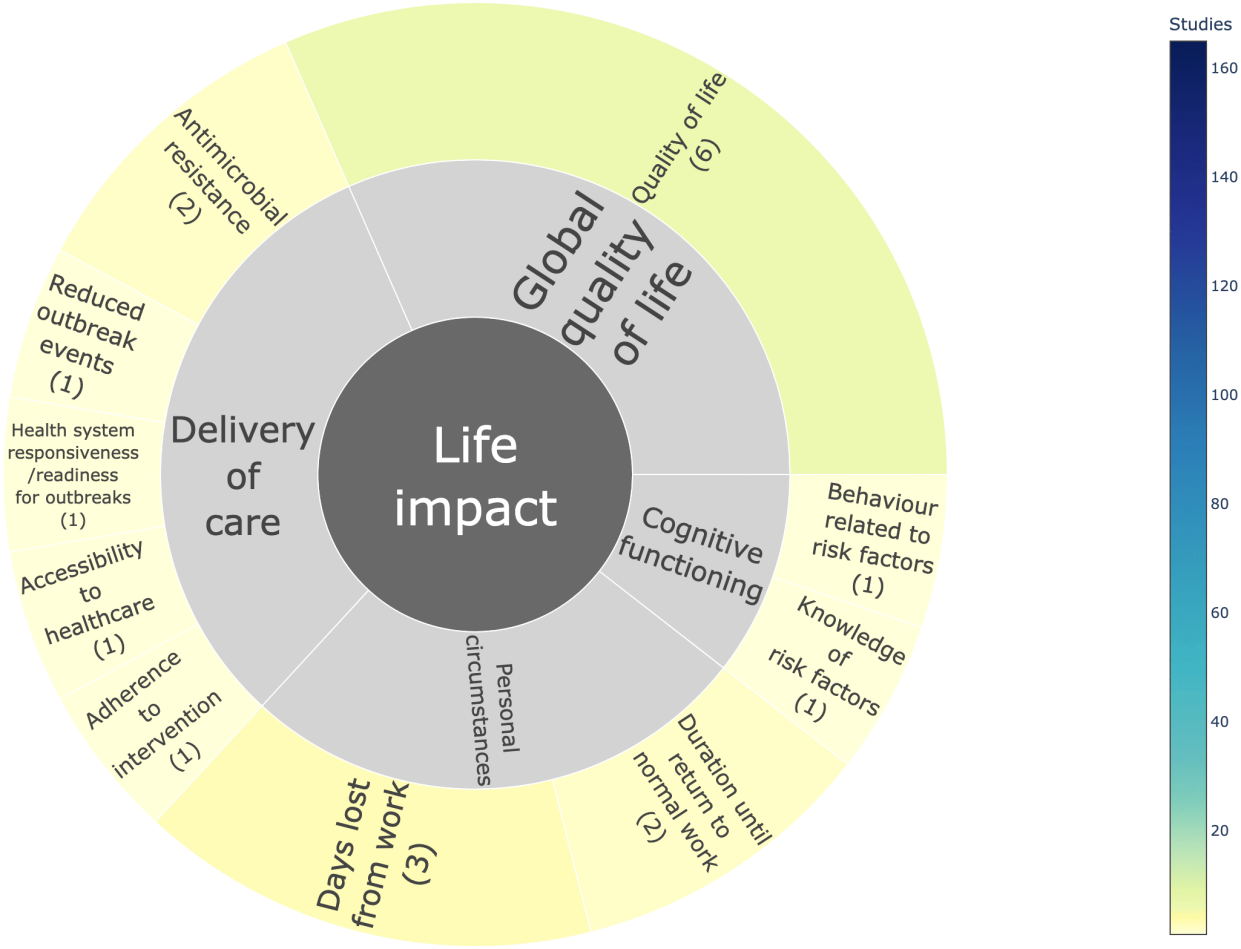
Sunburst chart demonstrating discrete outcomes and frequency of reporting by number of studies across COMET core area of life impact. Made using plotly on Python 3.13.

And finally, the COMET core area of life impact was the fifth most frequently reported core area (**Fig 8**; 4.0%; 12/298). There were four COMET domains included in this core area. The number of outcome types ranged from 1 – 5. All included outcomes were classified as benefit associated except for “Development of antimicrobial resistance” in the domain of delivery of care. The COMET domain of global quality of life included 1 outcome type reported in 2.0% (6/298) of studies. The COMET domain of personal circumstances included 2 outcome types reported in 1.7% (5/298) of studies. The COMET domain of delivery of care included 5 outcome type reported in 1.0% (3/298) of studies. The COMET domain of cognitive functioning included 2 outcome types reported in 0.3% (1/298) of studies. The proportion of studies reporting adverse events has remained consistently between absent or low throughout the included published years (**S7 Appendix**). Notably, outcomes in the life impact core area were the most infrequently reported, representing a key gap in the existing literature that future trials should seek to address.

### Outcome measures

There were 92 individual outcome measures which could be extracted from included studies, summarized in Table 2. These were categorized into 13 thematic areas, and then further sub-categorized by whether the measures were categorical or continuous.

**Table 2:** List of outcome measures thematically grouped and categorized by measure type.

Outcome Measure	Reported measure	Definition
Disease Severity	Categorical	• Death • In-hospital • In-Intensive Treatment Unit (ITU) • Modified sequential organ failure assessment (mSOFA) score (≤ 2 non-severe, > 2 severe) Composite outcomes • Multi-organ failure, persistent fever, death* • Hospitalisation accompanied by jaundice, acute kidney injury, and/or pulmonary involvement. • Organ dysfunction^§^ defined as a composite comprising serum creatinine > 180 µmol/L, aspartate aminotransferase > 100 IU/L, bilirubin >100 µmol/L, and platelet < 100 x 10^9^/L
Time measures of disease, organ failure, or treatment	Categorical	Mortality • Within 28 days • At 30 days • At 32 days Disease severity • Admission to hospital > 2 days • Manifestation within pre-defined time period • ≤ 48 hours • ≤ 21 days • ≥ 48 hours of treatment General clinical outcomes • Manifestation of chronic post-leptospirosis symptoms within 2 years • Resolution of symptoms < 5 weeks • Resolution of fever < 5 days • Change in clinical characteristics and/or functional signs and/or evolution of infection at 21 days Hepatobiliary outcomes • Proportion with normal liver function at 90 days • Change in serum bilirubin at 21 days Treatment outcomes • *Preventive* – change in antibody levels within 2 years • *Therapeutic* – treatment failure (see *) within 7 days of beginning antibiotic therapy Renal outcomes • Resolution of acute kidney injury within 7 days • Proportion with normal renal function at 90 days • Change in serum creatinine at 21 days Haematological outcomes • Change in haemoglobin levels at 21 days
	Continuous	Consecutive time in hours; applicable to • Clinical outcome: duration of bleeding Consecutive time in days; applicable to • Time to death • General clinical outcomes: duration of fever, duration of ITU admission, duration of antibiotics, duration of mechanical ventilation, duration of dialysis, duration of extracorporeal membrane oxygenation, duration of inotropes, resolution of symptoms, organ dysfunction^§^ • Renal outcomes: recovery of renal function • Disease severity Consecutive time in weeks; applicable to • Clinical outcome: duration in dialysis
Leptospira pathogen detection	Categorical	• Single positive microscopic agglutination test titre ranging from ≥ 1:100–1:400 • Positive serology using Enzyme-Linked Immunosorbent Assay (ELISA) • Positive culture from blood, urine, or tissue • Leptospires demonstrated in tissue by immunofluorescence, polymerase chain reaction, or immunostaining
Renal outcomes	Categorical	• Serum creatinine > 1.4 mg/dL • Estimated glomerular filtration rate (eGFR) <60 mL/min • Microalbuminaemia (albumin-to-creatinine ratio ≥ 30 mg/g in first morning urine catch) • Renal replacement therapy • Tubolopathy defined as presence of renal magnesium or phosphate wasting • Kidney Disease Improving Global Outcomes (KDIGO) criteria - cut-off points for serum creatinine and urine output • Persistent renal function decline of >25% in eGFR.
	Continuous	• Serum creatinine (point and maximum measures) • Serum urea • eGFR • Serum and urine neutrophil gelatinase-associated lipocalin • Serum kidney injury molecule-1 creatinine ratio
Inflammatory response	Continuous	• Point-measures of IL-2, IL-5, IL-7, IL-8, IL-10, IL-17, IL-1b, or TNF-α levels • Activated monocyte (CD14+ or CD16+) levels • Serum monocyte chemoattractant protein-1
Immune response	Categorical	In association with preventative treatment; • Presence of humoral immune response • ELISA IgG titre ≥ 20 • Seropositivity as measured by ELISA
Gastrointestinal outcomes	Categorical	Presence of pancreatitis complications • Acute peri-pancreatic fluid collection • Interstitial oedematous pancreatitis • Necrotising pancreatitis or walled-off necrosis
	Continuous	• Serum bilirubin
Haematological outcomes	Categorical	Thrombocytopenia • Platelet <100,000 x 10^9^/L
	Continuous	• Platelet count nadir • Haematocrit nadir
Neurological outcomes	Categorical	• Paralysis defined as facial palsy
Quality of life outcome	Categorical	• Euroquol EQ-5D questionnaire
Pregnancy outcomes	Categorical	• Preterm birth (<37 weeks) • Low birth weight (< 2500 grams)
Public health outcomes	Continuous	• Cost of therapy • Economic cost saving
Adverse event outcomes	Categorical	• Nosocomial infection; including • Ventilator-associated pneumonia • Urinary tract infection • Catheter-related bloodstream infection • Infected pressure ulcer • Symptoms or signs developing post-administration of study drug not reported prior

Measures marked by * and § are linked.

The median number of measures per thematic area was 3 (range 1–17). “Time measures of disease, organ failure, or treatment” and “renal outcomes” were the two thematic areas that had both categorical and continuous measures, and both contained the greatest number of measures by category. No patient reported outcome measures were identified. The wide variation in measure types across domains highlighted the absence of standardised outcome measurement in human leptospirosis research.

### Quality assessment and risk of bias

The inter-rater reliability during title and abstract screening was calculated as a Cohen’s kappa score of 0.71, indicative of substantial agreement. During full text review this was calculated as 0.92, indicative of excellent agreement.

Quality and risk of bias assessments by study category are provided in Table 3. The overall median QualSyst quality score for studies was 0.70 (interquartile range, IQR; 0.50 – 0.83). The median quality scores for interventional studies were the lowest amongst study types, with contributing factors being QualSystem tool category “No” scores in the sections of characterisation of subject/comparator (50%; 12/24), blinding of investigators and participants (62.5%; 15/24, and 66.7%; 16/24 respectively), sample size specification (66.7%; 16/24), and control of confounders (91.7%; 22/24). The median quality for observational studies were the second lowest amongst study types, with contributing factors being QualSyst tool category “No” scores in the sections of sample size specification (35.7%; 25/70), and control of confounders (60.0%; 42/70).

**Table 3:** Quality assessment and risk of bias results by study type.

	Interventional (n = 24)	Observational (n = 70)	Systematic review with meta-analysis (n = 9)	Other Analytic (n = 4)	Descriptive studies (n = 175)
**Quality Assessment** **(Median [IQR])**	0.52 (0.36 – 0.66)	0.68 (0.5 – 0.86)	0.81 (0.68 – 0.96)	0.98 (0.95 – 1)	0.70 (0.50 – 0.81)
**Risk of Bias** **Assessment (N [%])**					
No risk					
ORBIT B	8 (33.3)	26 (37.1)	6 (66.7)	–	–
ORBIT I	–	–	–	–	–
High risk					
ORBIT A	13 (54.2)	6 (8.6)	2 (22.2)	–	–
ORBIT D	–	2 (2.9)	–	–	–
ORBIT E	–	5 (7.1)	–	–	–
ORBIT G	–	1 (1.4)	–	–	–
Low risk					
ORBIT C	3 (12.5)	12 (17.1)	1 (11.1)	–	–
ORBIT F	–	18 (25.7)	–	–	–
ORBIT H	–	–	–	–	–

Descriptive studies include surveillance, narrative systematic reviews, case reports, and case series.

Other analytic studies include modelling and economic evaluations.

Observational studies include case-control, cross-sectional, Prospective/retrospective cohorts.

Only 34.4% (103/299) of studies could be analysed using the ORBIT scoring, which included interventional, observational, and systematic reviews with meta-analysis study designs. For interventional studies, 33.3% (8/24) were judged to have no risk of bias, 54.2% (13/24) a high risk of bias, and 12.5% (3/24) a low risk of bias. For studies with an observational design, 37.1% (26/70) were judged to have a risk of no bias, 20% (14/70) a high risk of bias, and 42.9% (30/7)) a low risk of bias. Finally, among systematic reviews with a meta-analysis component, 66.7% were judged to have no risk of bias, 22.2% (2/9) a high risk of bias, and 11.1% (1/9) a low risk of bias.

### Antibiotic interventions

**Fig 9:**
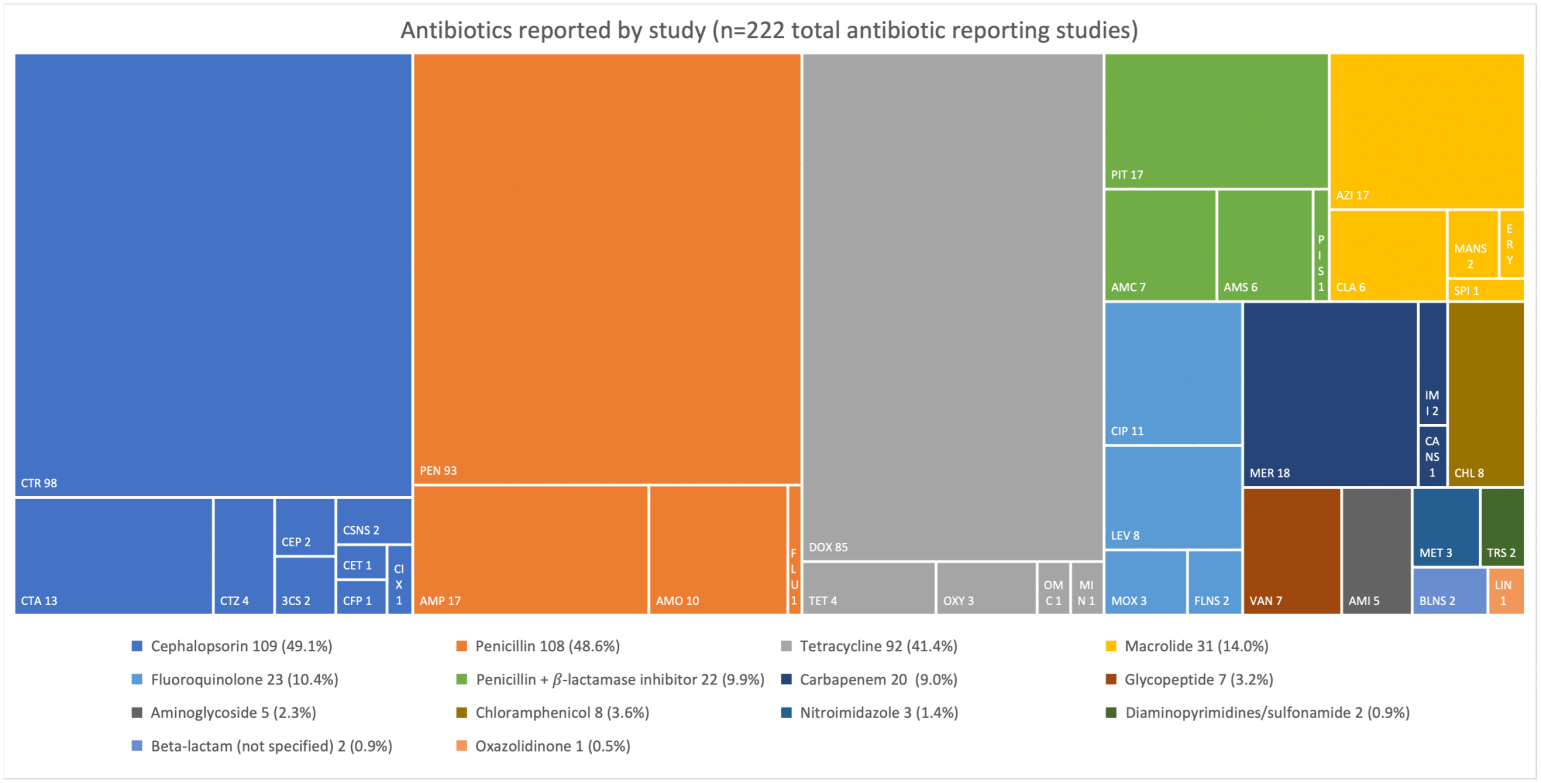
Treemap plot of antibiotics reported by studies. Abbreviations follow European Committee on Antimicrobial Susceptibility Testing nomenclature except for *: CTR Ceftiaxone, CTA Cefotaxime, CTZ Ceftazidime, CEP Cefepime, CET Cefalothin, *CFP Cefoperzone, CIX Cefixime, *3CS Third generation cephalosporin (not specified), *CSNS Cephalosporin (not specified), PEN Penicllin, AMO Amoxicillin, FLU Flucloxacillin, AMP Ampicillin, DOX Doxycyline, TET tetracycline, OXY Oxytetracycline, *OMC Omadacycline, MIN Minocycline, AZI Azithromycin, CLA Clarithromycin, ERY Erythromycin, *SPI Spiramycin, *MANS Macrolide (not specified), CIP Ciprofloxacin, LEV Levofloxacin, MOX Moxifloxacin, *FLNS Fluoroquinolone not specified, PIT Piperacillin-Tazobactam, AMC Amoxicillin-Clavulanic Acid, AMS Ampicllin-Sulbactam, *PIS Piperacillin-Sulbactam, MER Meropenem, IMI Imipenem, *CANS Carbapenem not specified, VAN Vancomycin, AMI Amikacin, CHL Chloramphenicol, MET Metronidazole, TRS Trimethoprim/sulfamethoxazole, *BLSN β-Lactam not specified, LIN Linezolid [19].

Antibiotics were reported as an intervention in 74.5% (222/298) of studies. A summary of all reported antibiotics is included in **Fig 9** and **S8 Appendix**. Cephalosporins (49.1%; 109/222), penicillins (48.6%; 108/222), and tetracyclines (41.4%; 92/222) were among the most frequently reported antibiotic classes. Ceftriaxone, penicillin, and doxycycline were the most frequent antibiotics used from the three aforementioned antibiotic classes respectively. Just under half (44.1%; 98/222) of studies reported using alternative antimicrobial regimens, and only 21 studies (9.5%; 21/222) reported on azithromycin or chloramphenicol – the only two non-β lactam/non-tetracycline antibiotics with existing randomised controlled trial evidence [17,18]. Almost half of the studies (46.8%; 104/222) reported on antibiotics which have no previous existing trial evidence to support their use in the treatment or prevention of leptospirosis [7,9]. Prior to 2000, the most commonly referenced antibiotic class was penicillin (68.2%; **S7 Appendix**) followed by tetracyclines (50.0%). Between 2000 – 2019, reporting shifted towards penicillin (49.3%) and cephalosporins (46.7%). Since 2020, cephalosporins (70.0%) and tetracyclines (60.0%) have been the most commonly referenced antibiotic classes.

### Organ-specific and other interventions

Immunosuppressive therapy was reported and specified across 41 different studies; 53.7% (22/41) case reports/series, 17.1% (7/41) interventional, 14.6% (6/41) observational, 9.8% (4/41) reviews, and 4.9% (2/41) trial/study protocol or registries. Additionally, immunosuppressive therapy was reported but not specified in 1 systematic review, 1 modelling study, and 5 case reports. Reported immunosuppressive treatments include methylprednisolone (58.5%; 24/41), prednisolone (31.7%; 13/41), dexamethasone (22.0%; 9/41), hydrocortisone (17.1%; 7/41), corticosteroid (non-specific; 4.9%; 2/41), cyclophosphamide (4.9%, 2/41), and cyclosporin (2.4%; 1/41).

Higher level clinical care or organ supportive therapy was reported by 44.0% (131/298) of studies. Reported interventions included intensive treatment unit (ITU) admission (18.1%; 54/298); respiratory supportive measures including high-flow nasal oxygen (0.7%; 2/298), non-invasive ventilation (4.0%; 12/298), and intubation/mechanical ventilation (19.8%; 59/298); and extracorporeal supportive therapies including plasma exchange (3.4%; 10/298), dialysis/haemofiltration (25.8%; 77/298), and extracorporeal membrane oxygenation (3.4%; 10/298).

Reported blood pressure support strategies included intravenous fluid resuscitation (15.1%; 45/298) and vasoactive inotropes (19.5%; 58/298). Diuretic treatment was an additional renal supportive treatment reported in some studies (4.4%; 13/298). Transfusion of blood products was reported in 12.8% (38/298) studies, and organ transplant in 0.3% (1/298).

Other less commonly reported interventions included health promotion strategies (0.7%; 2/298), N-acetyl cysteine as liver supportive care (0.3%; 1/298), and surveillance-only strategies (0.3%; 1/298).

## Discussion

This is the first comprehensive review of reported treatment outcomes, strategies, and measures for human leptospirosis drawing on global data from a broad range of study types. The geographical distribution of included studies shown in **Fig 2** aligns with global case burden estimates previously reported by Costa *et al* [1]. However, this review includes additional studies that also incorporate a range of clinical reports, which have not been previously comprehensively reviewed.

A wide range of outcome types are included, with all COMET Core Areas represented. The physiological/clinical core area had the most frequently reported outcomes, with the renal and urinary COMET domains accounting for 20% of this group. The life impact core area was the most underreported, highlighting a potential focus for future interventional trials.

Most of the included studies were of a descriptive nature, with over half comprising case reports, case series, narrative systematic reviews, literature reviews, study registries, or protocols. There was an escalation of publications reporting disease outcomes at the turn of the millennium, but case reports and case series continued to account for more than 50% of included studies for the decade. The inclusion of descriptive studies in this review may have limited the type of data that could be analysed. In addition, the number of case reports likely over represent outcomes that are of particular importance to clinicians at the expense of patient reported outcomes.

The issues identified in this review such as the heterogeneous outcome reporting or limited numbers of patient-reported outcomes are not unique to leptospirosis and have been documented across other neglected tropical diseases [20,21]. However, leptospirosis presents particular challenges given the breadth of its clinical spectrum and the diversity of affected populations and health system contexts. The additional challenges of diagnostic uncertainty in endemic settings and syndromic overlap with other acute febrile illnesses further compounds outcome inconsistency, and emphasises the need for a disease-specific COS.

### Quality and risk of bias assessments

The QualSyst tool provides more granular detail in the final quality assessment for design types such as interventional or observational analytical studies [15]. This is because factors such as study population characteristics, blinding, sample size calculation, and control of confounders are more relevant to these study designs and are critical to the accurate measurement and validity of the study’s stated outcomes. Despite this, it is notable that both interventional and observational studies scored poorly on these criteria when assessed. Weaknesses in these areas may have introduced outcome reporting bias in certain studies by affecting which outcomes are reported or how outcomes are described, consequently influencing the outcomes included in this review.

There is remarkable outcome reporting bias across the major study types identified in this review. In our assessment, the proportion of studies at-risk of bias does not decrease as more rigorous study designs are used. Both interventional and observational analytical studies reported either a high or low risk of bias in about two thirds of included studies respectively. It is particularly notable that just over half of included trials were at high risk of outcome reporting bias – though perhaps not surprising given that trials were often single centre and underpowered.

These assessments were undertaken to provide context regarding the characteristics and reporting practices of included studies, rather than to inform study exclusion. Consistent with the objectives of the wider COS-LEP project, all reported outcomes were retained to ensure comprehensive outcome identification. Decisions regarding exclusion or prioritisation will be undertaken in subsequent consensus-building stages of the COS-LEP project [12].

### Outcome reporting

We demonstrate in this review the amount of heterogeneity in reported outcomes across the various different types of leptospirosis studies. This inconsistency has been previously highlighted in other healthcare research fields, and poses a significant challenge to synthesizing the necessary information to guide patient management or wider leptospirosis health policy.[22]

We identified that outcomes in the physiological/clinical core area were the predominant efficacy-related outcomes across all study types, particularly in the domains of renal, respiratory, infectious, and general outcomes. The common theme in the outcomes between these domains was the prevention of different manifestation of severe leptospirosis and/or clinical deterioration, highlighting the importance placed on managing these as a treatment goal. This is also reflected in the second most commonly reported core area of resource use – where the top five outcomes (complete medical discharge from health services, length of hospitalisation, ITU admission, the need for dialysis, and ITU length of stay) were all strongly influenced by severe leptospirosis disease.

The inclusion of case reports and series in this review likely led to an increase in reporting of outcomes of priority to healthcare professionals. However, the inclusion of these types of descriptive studies was intentional and pre-defined to ensure comprehensive identification of outcomes reported in the literature consistent with established COMET methodology for outcome mapping in core outcome set development [10,12]. Case reports and series often report rare, unexpected, or clinically important outcomes which may not be captured in other study designs with less clinical focus. Restricting inclusion to analytical studies could have introduced a selection bias towards more investigator-selected endpoints, and risked overlooking outcomes relevant to clinical practice.

Regardless of this limitation, it is notable that outcomes that would be important to other stakeholders such as patients and policymakers, including quality-of-life or treatment cost-effectiveness, were infrequently reported. Despite the growing recognition in clinical trial design of the need for treatment evaluation to extend beyond exclusive clinical endpoints and incorporate patient-reported outcomes of interest, these were concerningly absent from many included studies [23]. Quality of life, an indicator of treatment benefit from the patient perspective, is increasingly becoming an important measure to gauge treatment effect on patients, but was only extracted in this review from two trial protocols [24]. Similarly, there were a limited numbers studies that assessed economic outcomes or cost effectiveness, despite their critical importance to national or institutional governing bodies and decision-makers seeking to implement best practice guidelines. Taken together, these findings suggest that the current treatment evidence base is insufficiently aligned to wider stakeholder priorities. Addressing this gap in patient-reported outcomes will be essential to the COS-LEP development process, and will be explored through a dedicated qualitative study engaging patients and healthcare providers to identify outcomes of greatest relevance.

Harms outcomes accounted for a small proportion of outcomes reported across all studies - 11.7% (35/298). Of these only 17.1% (6/35) were reported in randomised controlled trials. The lack of safety outcome reporting in trials is a wider methodological issue in trial design which can contribute to reporting biases and misrepresentation of an intervention as a safe or preferable option regardless of intervention efficacy [25]. Assessing harms outcomes can provide a broader overview of the risk-benefit of an intervention, for example linking closely to quality of life or treatment adherence [26]. A distinct harms outcome that can occur following antimicrobial treatment of spirochaetal infections such as leptospirosis is a Jarisch-Herxheimer event, a temporary systemic febrile inflammatory reaction [27]. Despite this being of high clinical relevance to health professional and patients, it is only reported in 4.0% (11/298) of studies. Given the lack of reported qualitative studies seeking patient or healthcare provider perspectives on leptospirosis treatment outcomes, exploration of harms outcomes of interest to these groups will be vital to the construction of a future COS.

Given the large number of outcomes identified at this early stage, a key consideration will be focusing the final outcome set on measures that are feasible to implement in low-resource endemic settings. Outcomes and measures selected must be practically achievable within the constraints of local health systems where leptospirosis burden is greatest, including limitations in laboratory infrastructure, clinical staffing, and research data collection capacity. This consideration will be explicitly incorporated into the COS-LEP Delphi and final consensus process.

The policy and public health implications of establishing a COS extend beyond trial methodology. Constructing a standardised outcome set is an essential step towards producing research that can strengthen the evidence base needed to update WHO treatment guidelines, which currently rely on limited and methodologically weak trial data. Consistent outcome reporting across future trials would support antimicrobial stewardship efforts by enabling reliable comparative evaluation of treatment regimens, and inform procurement and resource allocation decisions by health institutions in endemic settings. The process of defining core outcomes and their measurements would also represent the first formal standardisation of certain leptospirosis-specific outcomes, such as pulmonary involvement and Jarisch-Herxheimer reactions, neither of which has been consistently defined across studies and whose precise characterisation is important for both clinical practice and cross-trial comparability.

### Outcome measure

We compile the first comprehensive description of outcomes measures for human leptospirosis disease reported in the literature. The most frequently reported outcome measure category includes 25 chronological time measures categorized across continuous and categorical measures. Even within this category there are varying lengths of time. For example, the measure of severe disease manifestation within a pre-defined time period included ≤ 48 hours, ≥ 48 hours, and  ≤ 21 days following treatment. Depending on the organ system involved, an outcome may not be captured within these time periods. This discrepancy highlights the need to standardise measures used to enable better comparability of results.

We demonstrate that even in the most frequently reported outcome domain there is a wide inconsistency in types of measure. For example, within the domain of renal and urinary outcomes alone there are least 12 different measures consisting of routinely available diagnostics to more experimental tests. We did not identify any patient reported outcome measures in included studies.

Multi-organ involvement is a critical sequela of leptospirosis and a major cause of disease severity.[5] As previously noted, a precise definition of this outcome will be essential in future trials. We report several measures used to capture this outcome, ranging from mortality alone to composite parameters. Composite outcome measures can more accurately capture disease manifestation across one or multiple organ systems, and the use of established scoring criteria offers the advantage of validated, semi-quantitative systems. For example, the modified sequential organ failure assessment score is an established criteria that has been previously reported to guide leptospirosis therapy, and which offers pragmatic and clinically relevant endpoints that would be useful in large Phase III trials [28–30].

Acute kidney injury (AKI) is a common manifestation in leptospirosis disease [31]. Clinical endpoints in trials focused on AKI have commonly relied on measuring small short-term changes in creatinine levels or composite endpoints of long term major adverse kidney events, with several limitations in both approaches [32]. Previous leptospirosis studies have used a range of renal outcome measures to capture AKI events, including biochemical thresholds or the presence of oliguria/anuria, all generally aligned with internationally accepted criteria [33–35]. In addition to the immediate disease process, progression to chronic kidney disease (CKD) after acute leptospirosis infection has been previously documented [36]. According to KDIGO guidelines, CKD is defined as abnormalities of kidney structures or function, present for a minimum of 3 months, with implications for health [37]. Future trial will need to incorporate short and long-term renal outcomes to comprehensively capture this domain.

Other outcomes measures of organ involvement are less well defined and will require a consensus on both measurement and stratification of clinical importance. Of the remaining organ system outcome domains reported in the Physiological/Clinical core area, only 5 had defined associated outcome measures. Pulmonary complications, ranging from acute respiratory distress syndrome (ARDS) to pulmonary haemorrhage, represent severe manifestations associated with an elevated mortality risk [38]. Thrombocytopenia is another well-documented complication linked to clinical bleeding risk, though platelet count thresholds have varied across studies [39]. Reversible hepatic injury is a hallmark of severe leptospirosis, but while associated with an increased mortality risk in observational studies, it rarely progresses to liver failure [40]. Pancreatitis, the primary gastrointestinal complication with reported outcome measures, is an emerging syndrome of interest that often necessitates intensive care management [41]. Neurological manifestations are diverse, ranging from a meningoencephalitis to paralysis, and are also recognized complications of leptospirosis [42].

### Treatment strategies

This systematic review provided an opportunity to compile the first comprehensive list of antimicrobial, immunosuppressive, and other organ-supportive treatments used in the management of human leptospirosis. These data are invaluable to inform discussions relating to a control arm regimen in future clinical trials, and the design of a systematic assessment of treatment options for optimal use. Importantly, these findings are presented to contextualise outcome variability and research practices, rather than to evaluate comparative effectiveness.

Current, although now somewhat outdated, World Health Organisation (WHO) guidance recommends that antibiotic choice be guided by timing of presentation and disease severity [43]. For mild disease, recommended regimens include oral antibiotics such as amoxicillin, ampicillin, doxycycline, or erythromycin. For severe cases, intravenous penicillin is preferred. Third generation cephalosporins or quinolones are noted as potential alternatives. Despite existing previous published international and national guidance on choice of antimicrobial, this review demonstrates that this is not reflective of real-world use of treatments. This is possibly due to a wide range of factors including availability, drug quality assurance, suitable health facilities to dispense and administer medication, direct and indirect costs, wider antimicrobial stewardship concerns, and uncertainty of diagnosis. We could not reliably extract information on treatment regimen composition, and although many listed antibiotics were not given as monotherapy, the dosing regimen information was incomplete and variable between studies. Although the evidence of antimicrobial resistance in human leptospirosis remains unclear due to methodological issues with antimicrobial susceptibility testing, it is generally accepted that there are no emerging resistance issues with the organism [44,45]. Consequently, data on resistance patterns was not systematically reported in included studies, if at all.

Cephalosporins, penicillins, and tetracycline antibiotic classes accounted for the majority of reported antimicrobial regimens in this review - in line with WHO recommendations as well as national guidance from countries where leptospirosis is a public health concern [46–48]. However, there is great variation of within-class choice, method of delivery, and dosing. We show that over half of the studies report the use of alternative antibiotics, and nearly 20% describe therapies with no supporting trial evidence or to which leptospirosis may demonstrate a potential intrinsic resistance [7,45]. These findings suggest an incoherence in antimicrobial strategy at all levels, with profound implications for standardising clinical guidelines, resource planning, and wider antimicrobial stewardship considerations.

Other non-antimicrobial strategies, such as organ-supportive therapies and non-pharmacological interventions, were reported with varying frequency. Renal replacement therapy was among the most frequently described, reported in nearly 20% of studies. Although findings from observational studies suggest a possible survival benefit, this has not been confirmed in randomised clinical trials [49]. Respiratory supportive strategies such as mechanical ventilation often employing lung-protective ventilation were reported at a similar frequency, but likewise lacks validation from trial evidence.

Despite the limited evidence supporting immunosuppressive treatment for leptospirosis there were several studies, which also included randomised controlled trials, that explored the use of different immunosuppressive agents [8]. Similar to antimicrobial regimens, information on dosing and choice of agent was variable between studies.

### Limitations and deviations from protocol

Despite our methodology not limiting study inclusion by language, there may still exist studies not published or indexed in English which will have been missed. We have however demonstrated data capture across 63 countries. There may have been unintended reviewer bias introduced during screening of publications, particularly more narrative-type studies, however we demonstrate a high degree of inter-reviewer agreement in our analysis.

Machine translation was used for a small proportion of non-English studies. While this may have introduced inaccuracies in the interpretation of nuanced clinical terminology, the majority of included studies were published in English (87.9%). Furthermore, data extraction focused on discrete outcome names, definitions, and study characteristics rather than interpretative content, limiting the potential impact of translation errors on findings.

There were no qualitative studies identified during this review, although this was expected in our published protocol. We did not perform subgroup analysis as per our protocol as the majority of studies either did not describe this in sufficient detail or omitted this information. We performed a limited comparison of outcomes and treatments by region and publication time period. These questions, along with more granular analyses of treatments and patient characteristics at the individual level, require further investigation through dedicated descriptive or analytical multi-national epidemiological studies.

## Conclusion

There is a clear lack of consensus for outcome reporting and core outcome measures in the field of human leptospirosis. The findings of this review support the development of a core outcome and core outcome measures set to address this disparity. Given the range of outcome domains identified, it would be prudent to explore the use of a composite outcome in the final core outcome set, to inform appropriate primary and secondary endpoints for future evaluation of treatments. A dedicated qualitative study is required to explore the experiences and perspectives of the general public who have experienced leptospirosis, as these insights are inadequately captured by the existing quantitative evidence. This would ensure that outcomes important to patients are appropriately identified and incorporated into the COS development process. Identifying and standardizing the measure for any outcome chosen in the final core outcome set will be vital. This review lays the foundation for a consensus-driven core outcome set for leptospirosis, with the potential to meaningfully improve trial quality and comparability, and ultimately drive the production of evidence to guide patient care globally.

## Supporting information

S1 Appendix PRISMA checklist. From: Page MJ, McKenzie JE, Bossuyt PM, Boutron I, Hoffmann TC, Mulrow CD, et al. The PRISMA 2020 statement: an updated guideline for reporting systematic reviews. BMJ 2021;372:n71. https://doi.org/10.1136/bmj.n71. This work is licensed under CC BY 4.0. To view a copy of this license, visit https://creativecommons.org/licenses/by/4.0/
(DOCX)

S2 Appendix PRISMA abstract checklist. From: Page MJ, McKenzie JE, Bossuyt PM, Boutron I, Hoffmann TC, Mulrow CD, et al. The PRISMA 2020 statement: an updated guideline for reporting systematic reviews. BMJ 2021;372:n71. https://doi.org/10.1136/bmj.n71. This work is licensed under CC BY 4.0. To view a copy of this license, visit https://creativecommons.org/licenses/by/4.0/
(DOCX)

S3 Appendix Systematic review search strategies. (DOCX)

S4 Appendix Summary of data extraction variables extracted during full-text review using Covidence systematic review software. (DOCX)

S5 Appendix Summary of quality and outcome reporting bias assessment tools used in the systematic review. ORBIT – Outcome Reporting Bias in Trials.
(DOCX)

S6 Appendix Discrete outcome types and frequency of reporting in studies. Reported outcomes categorized by COMET core area, domain, and association with benefit or harm. DIC – Disseminated Intravascular Coagulation, ECG – electrocardiogram, MAP – mean arterial pressure, CKD – chronic kidney disease, ECMO – extracorporeal membrane oxygenation, JHR - Jarisch-Herxheimer reaction *Compound outcome - two compound outcomes are included: MAKE (classified between mortality/survival, renal and urinary, and need for further intervention domains) and normalisation of cardio-respiratory function (classified between cardiac and respiratory/thoracic/mediastinal domains).
(DOCX)

S7 Appendix Summary of reporting region, design of study, reported outcome domains, and antibiotic class by studies published <2000, 2000 – 2019, and ≥2020. (DOCX)

S8 Appendix Tabulated summary of antibiotics reported by studies. (DOCX)
