# Supplementary material for: Efficacy and safety outcomes reported in human leptospirosis studies to inform the development of a core outcome and core outcome measurement set: A systematic review
Source: PLoS Negl Trop Dis. 2026 Jul 13;20(7):e0013651. doi: 10.1371/journal.pntd.0013651 (PMC13395454; doi:10.1371/journal.pntd.0013651)
Supplement: S3 Appendix — (DOCX) [file pntd.0013651.s003.docx]

Cochrane Database of Systematic Reviews (Issue 3 of 12, March 2023)

#1 MeSH descriptor: [Leptospirosis] explode all trees

#2 (leptospir* or (weil* disease))

#3 #1 or #2

#4 (accept* or attitude or barrier or behaviour or challenge or (clinical utility) or (decisional conflict) or (decision making) or experience or (informed consent) or knowledge or outcomes or pyschosocial or (patient satisfaction) or understanding or uptake or (willingness to pay))

#5 #3 and #4

TOTAL

58 RETURNS (21/3/2023; Issue 3 of 12, March 2023)

61 RETURNS (23/11/2023, Issue 11 of 12, November 2023)

70 RETURNS (28/5/2025, Issue 5 of 12, May 2025)

Cochrane Central Register of Controlled Trials (Issue 2 of 12, February 2023)

#1 MeSH descriptor: [Leptospirosis] explode all trees

#2 (leptospir* or (weil* disease))

#3 #1 or #2

#4 (accept* or attitude or barrier or behaviour or challenge or (clinical utility) or (decisional conflict) or (decision making) or experience or (informed consent) or knowledge or outcomes or pyschosocial or (patient satisfaction) or understanding or uptake or (willingness to pay))

#5 #3 and #4

TOTAL

326 RETURNS (21/3/2023, Issue 2 of 12 February 2023)

341 RETURNS (23/11/2023, Issue 10 of 12, October 2023)

358 RETURNS (28/5/2025, Issue 4 of 12, April 2025)

**MEDLINE Ovid (1946 to May 23, 2025)**

1. Ovid MEDLINE(R) ALL
2. 1 Leptospirosis.mp. or exp Leptospirosis/ 11055
3. 2 (leptospir* or weil* disease).mp. [mp=title, book title, abstract, original title, name of substance word, subject heading word, floating sub-heading word, keyword heading word, organism supplementary concept word, protocol supplementary concept word, rare disease supplementary concept word, unique identifier, synonyms, population supplementary concept word, anatomy supplementary concept word] 14145
4. 3 1 or 2 14145
5. 4 human.mp. or Humans/ 21752264
6. 5 3 and 4 7602
7. 6 acceptability.mp. 51135
8. 7 attitude.mp. 323543
9. 8 barrier.mp. 235024
10. 9 behaviour.mp. 241300
11. 10 challenge.mp. 439793
12. 11 clinical utility.mp. 33267
13. 12 decisional conflict.mp. 1366
14. 13 decision making.mp. 269920
15. 14 experience.mp. 806446
16. 15 informed consent.mp. 71399
17. 16 knowledge.mp. 964927
18. 17 outcomes.mp. 1370326
19. 18 psychosocial.mp. 121667
20. 19 patient satisfaction.mp. or patient satisfaction/ 113573
21. 20 understanding.mp. 1103309
22. 21 uptake.mp. 444914
23. 22 willingness to pay.mp. or Willingness To Pay/ 8334
24. 23 6 or 7 or 8 or 9 or 10 or 11 or 12 or 13 or 14 or 15 or 16 or 17 or 18 or 19 or 20 or 21 or 22 5482865
25. 24 consensus.mp. or consensus/ or consensus development/ 219965
26. 25 feasibility study/ or feasibility.mp. 260060
27. 26 pilot study.mp. 114203
28. 27 (program development or evaluation).mp. [mp=title, book title, abstract, original title, name of substance word, subject heading word, floating sub-heading word, keyword heading word, organism supplementary concept word, protocol supplementary concept word, rare disease supplementary concept word, unique identifier, synonyms, population supplementary concept word, anatomy supplementary concept word] 1921424
29. 28 (random* or blind* or placebo* or meta-analys*).mp. [mp=title, book title, abstract, original title, name of substance word, subject heading word, floating sub-heading word, keyword heading word, organism supplementary concept word, protocol supplementary concept word, rare disease supplementary concept word, unique identifier, synonyms, population supplementary concept word, anatomy supplementary concept word] 2012552
30. 29 (Surveys and Questionnaires).mp. [mp=title, book title, abstract, original title, name of substance word, subject heading word, floating sub-heading word, keyword heading word, organism supplementary concept word, protocol supplementary concept word, rare disease supplementary concept word, unique identifier, synonyms, population supplementary concept word, anatomy supplementary concept word] 564047
31. 30 Quantitative.mp. 771909
32. 31 24 or 25 or 26 or 27 or 28 or 29 or 30 5190843
33. 32 5 and 23 and 31 148

TOTAL

148 RETURNS (21/3/2023, 1946 to March 20, 2023)

158 RETURNS (23/11/2023, 1946 to November 22, 2023)

173 RETURNS (28/5/2023, 1946 to May 23, 2025)

**Run 1+2**

**EMBASE Ovid**

**Rune 3
Embase classic + Embase Ovid**

1 Leptospirosis.mp. or exp Leptospirosis/ 14571

2 (leptospir* or weil* disease).mp. [mp=title, abstract, heading word, drug trade name, original title, device manufacturer, drug manufacturer, device trade name, keyword heading word, floating subheading word, candidate term word] 19155

3 1 or 2 19155

4 human.mp. or Humans/ 27520062

5 3 and 4 9805

6 acceptability.mp. 66698

7 attitude.mp. 538302

8 barrier.mp. 331883

9 behaviour.mp. 327269

10 challenge.mp. 572571

11 clinical utility.mp. 50298

12 decisional conflict.mp. 2019

13 decision making.mp. 552584

14 experience.mp. 1281906

15 informed consent.mp. 158344

16 knowledge.mp. 1183800

17 outcomes.mp. 2153432

18 psychosocial.mp. 185515

19 patient satisfaction.mp. or patient satisfaction/ 178133

20 understanding.mp. 1354661

21 uptake.mp. 633892

22 willingness to pay.mp. or Willingness To Pay/ 13609

23 6 or 7 or 8 or 9 or 10 or 11 or 12 or 13 or 14 or 15 or 16 or 17 or 18 or 19 or 20 or 21 or 22 7838835

24 consensus.mp. or consensus/ or consensus development/ 316673

25 feasibility study/ or feasibility.mp. 372297

26 pilot study.mp. 239232

27 (program development or evaluation).mp. [mp=title, abstract, heading word, drug trade name, original title, device manufacturer, drug manufacturer, device trade name, keyword heading word, floating subheading word, candidate term word] 2608953

28 (random* or blind* or placebo* or meta-analys*).mp. [mp=title, abstract, heading word, drug trade name, original title, device manufacturer, drug manufacturer, device trade name, keyword heading word, floating subheading word, candidate term word] 2889281

29 (Surveys and Questionnaires).mp. [mp=title, abstract, heading word, drug trade name, original title, device manufacturer, drug manufacturer, device trade name, keyword heading word, floating subheading word, candidate term word] 12429

30 Quantitative.mp. 1245015

31 24 or 25 or 26 or 27 or 28 or 29 or 30 6872498

32 5 and 23 and 31 231

Total hits

231 RETURNS (21/03/2023, 1974 to March 2023)

243 RETURNS (23/11/2023, 1974 to 2023 November 22)

299 RETURNS (28/5/2025, 1947 to 2025 May 23)

**LILACS**

(leptospirosis)

AND ((leptospir* OR weil* disease))

AND (human)

AND (accept* OR attitude OR barrier OR behaviour OR challenge OR (clinical utility) OR (decisional conflict) OR (decision making) OR experience OR (informed consent) OR knowledge OR outcomes OR pyschosocial OR (patient satisfaction) OR understanding OR uptake OR (willingness TO pay))

AND (consensus OR feasib* OR pilot study OR (program development) OR (random* or blind* or placebo* or meta-analys*) OR (Surveys and Questionnaires) OR Quantitative)

Total hits

674 RETURNS (21/03/2023)

728 RETURNS (23/11/2023)

802 RETURNS (28/5/2025)

**Web of Science (1970 – 2023)**

Web of Science Search Strategy (v0.1)

# Database: Web of Science Core Collection

# Entitlements:

- WOS.SCI: 1970 to 2023

- WOS.AHCI: 1975 to 2023

- WOS.ESCI: 2018 to 2023

- WOS.ISTP: 1990 to 2023

- WOS.SSCI: 1970 to 2023

- WOS.ISSHP: 1990 to 2023

1: ALL=(Leptospirosis)

2: ALL=((leptospir* or (weil* disease)))

3: #2 OR #1

4: ALL=(Human)

5: #3 AND #4

6: ALL=(accept* OR attitude OR barrier OR behaviour OR challenge OR (clinical utility) OR (decisional conflict) OR (decision making) OR experience OR (informed consent) OR knowledge OR outcomes OR pyschosocial OR (patient satisfaction) OR understanding OR uptake OR (willingness TO pay))

7: ALL=(consensus OR feasib* OR pilot study OR (program development) OR (random* or blind* or placebo* or meta-analys*) OR (Surveys and Questionnaires) OR Quantitative)

8: #5 AND #6 AND #7

Total hits

2,584 RETURNS (21/03/23)

2,787 RETURNS (23/11/23)

3,388 RETURNS (28/5/25)

**OPENSigle (GreyNet)**

1. Leptospirosis

14 RETURNS (21/03/23)

14 RETURNS (23/11/23)

8 RETURNS (28/5/25)

**Clinicaltrials.gov**

1. Leptospirosis

16 RETURNS (23/11/23)

18 RETURNS (28/5/25)
