# Supplementary material for: Efficacy and safety outcomes reported in human leptospirosis studies to inform the development of a core outcome and core outcome measurement set: A systematic review
Source: PLoS Negl Trop Dis. 2026 Jul 13;20(7):e0013651. doi: 10.1371/journal.pntd.0013651 (PMC13395454; doi:10.1371/journal.pntd.0013651)
Supplement: S4 Appendix — (DOCX) [file pntd.0013651.s004.docx]

| Data extraction variable and tools | | |
| --- | --- | --- |
| Category | Variables extracted | Tool |
| Study information | Year of publication, institution, country, study design, study inclusion/exclusion criteria | Covidence |
| Study population | Demographics, epidemiological risk factors | Covidence |
| Interventions | Treatment type, dosing, regimen details | Covidence |
| Outcomes | Outcome name | Covidence |
| Outcome measures | Measurement instrument, definition, categorical or continuous | Covidence |
| Study conclusion | Verbatim conclusion as reported by authors | Covidence |

**S4 Appendix - Summary of data extraction variables extracted during full-text review using Covidence systematic review software.**
