# Supplementary material for: Efficacy and safety outcomes reported in human leptospirosis studies to inform the development of a core outcome and core outcome measurement set: A systematic review
Source: PLoS Negl Trop Dis. 2026 Jul 13;20(7):e0013651. doi: 10.1371/journal.pntd.0013651 (PMC13395454; doi:10.1371/journal.pntd.0013651)
Supplement: S5 Appendix — ORBIT – Outcome Reporting Bias in Trials. (DOCX) [file pntd.0013651.s005.docx]

| Category | Assessment tool | Method |
| --- | --- | --- |
| Quality assessment | QualSyst | Data collected in Covidence. Quality score calculated using QualSyst algorithm by study design |
| Outcome reporting bias | ORBIT | Data collected in Covidence. Risk of bias classification assigned using ORBIT tool |

**S5 Appendix - Summary of quality and outcome reporting bias assessment tools used in the systematic review.** ORBIT – Outcome Reporting Bias in Trials
