# Supplementary material for: Efficacy and safety outcomes reported in human leptospirosis studies to inform the development of a core outcome and core outcome measurement set: A systematic review
Source: PLoS Negl Trop Dis. 2026 Jul 13;20(7):e0013651. doi: 10.1371/journal.pntd.0013651 (PMC13395454; doi:10.1371/journal.pntd.0013651)
Supplement: S6 Appendix — Reported outcomes categorized by COMET core area, domain, and association with benefit or harm. DIC – Disseminated Intravascular Coagulation, ECG – electrocardiogram, MAP – mean arterial pressure, CKD – chronic kidney disease, ECMO – extracorporeal membrane oxygenation, JHR - Jarisch-Herxheimer reaction *Compound outcome - two compound outcomes are included: MAKE (classified between mortality/survival, renal and urinary, and need for further intervention domains) and normalisation of cardio-respiratory function (classified between cardiac and respiratory/thoracic/mediastinal domains). (DOCX) [file pntd.0013651.s006.docx]

| **S6 Appendix. Discrete outcome types and frequency of reporting in studies.** | | | | | | |
| --- | --- | --- | --- | --- | --- | --- |
| **COMET Core Area** | **COMET Domain** | **Harm or Benefit Outcome** | **COS-LEP Discrete Outcome** | | | |
|  |  |  | **Outcome** | **N** | **Outcome** | **N** |
| Death | Mortality/  Survival | Benefit | **Mortality** (1–165) | 165 | **Major Adverse Kidney Event (MAKE)*** (30) | 1 |
|  |  |  | **Mortality attributable to leptospirosis**  (41,141) | 2 | **Survival at defined time point** (12,166,167) | 3 |
|  |  |  | **Time to death**  (54,67,111,112,129,143,162) | 7 |  |  |
| Physiological/clinical | Blood and lymphatic system | Benefit | **Anaemia** (91,104,168–170) | 5 | **Platelet count** (17,121,171) | 3 |
|  |  |  | **Duration of anaemia** (170) | 1 | **Thrombocytopenia**  (25,44,61,74,89,104,121,151,164,168,172) | 11 |
|  |  |  | **Change in haemoglobin levels** (173) | 1 | **Haematocrit count** (121) | 1 |
|  |  |  | **Revealed bleed**  (3,33,40,47,88,121,128,137,148,149,154,160,163,164,172,174,175) | 17 | **DIC** (94,105,147) | 3 |
|  |  |  | **Duration of bleeding** (156,176) | 2 | **Normalization of haematological parameters** (19,176–180) | 6 |
|  |  |  | **Leucocytosis** (168) | 1 | **Improvement of platelet counts** (18,167) | 2 |
|  |  |  | **Erythrocyte sedimentation rate** (168) | 1 | **Days until normalization of platelets** (18,19) | 2 |
|  | Cardiac | Benefit | **Cardiac manifestations (non-specific)**  (2,63,87,92,109,112,133,148,155,181,182) | 11 | **Cardiac arrythmia** (8,92,159) | 3 |
|  |  |  | **ECG abnormalities** (183) | 1 | **Normalization of cardio-respiratory functions*** (179) | 1 |
|  | Ear and labyrinth | Benefit | **Hearing** (184) | 1 |  |  |
|  | Eye | Benefit | **Conjunctival suffusion (prevention)** (42) | 1 | **Duration of conjunctival suffusion (prevention)** (185) | 1 |
|  | Gastro-intestinal | Benefit | **Pancreatitis (general)** (31,62,72,159) | 4 | **Bedside index for severity in acute pancreatitis** (31) | 1 |
|  |  |  | **Pancreatitis (by subtype)** (31) | 1 | **Duration of gastrointestinal symptoms** (185) | 1 |
|  |  |  | **Pancreatitis complications** (31) | 1 | **Gastrointestinal haemorrhage** (24,45,112,163) | 4 |
|  | General | Benefit | **Morbidity** (136,186) | 2 | **Change in clinical characteristics** (21,173,177,187) | 4 |
|  |  |  | **Haemodynamic shock**  (25,33,40,69,77,87,92,94,99,109,116,149,172,177,188–191) | 18 | **Change in functional signs** (173) | 1 |
|  |  |  | **Multi-organ failure**  (3,25,33,38,40,67,81,97,99,118,123,159,162,188,192–196) | 19 | **Inadequate clinical response** (20) | 1 |
|  |  |  | **Collapse** (44) | 1 | **Time to resolution of organ dysfunction** (66) | 1 |
|  |  |  | **Duration of vomiting** (44) | 1 | **Resolution of symptoms (non-specific)**  (197–207) | 11 |
|  |  |  | **Duration of myalgia** (42,185) | 2 | **Time to resolutions of symptoms (non-specific)** (56,177,201,208–210) | 6 |
|  |  |  | **Duration of general symptoms** (178,211) | 2 | **Resolution of symptoms at set time period** (168) | 1 |
|  |  |  | **Development of any complication at set timepoint** (212) | 1 | **Duration of malaise** (185) | 1 |
|  | Hepato-biliary | Benefit | **Liver failure**  (17,44,77,80,81,89,126,133,135,148,162,163,172,193,213–215) | 17 | **Change in bilirubin values** (173,188) | 2 |
|  |  |  | **Hepatic tenderness** (178) | 1 | **Normalization of liver function**  (18,42,65,135,178–180,202,204,205,207,216–226) | 22 |
|  |  |  | **Clinical jaundice** (3,27,175,211,215) | 5 | **Need for cholecystectomy** (227) | 1 |
|  |  |  | **Hyperbilirubinaemia** (25,104,168,193,205,228) | 6 | **Time to normalisation of liver function** (19) | 1 |
|  | Immune system | Benefit | **Immune/inflammatory biochemical markers** (114,127,171,187,229,230) | 6 | **Improvement of parameters of oxidative stress and increase antioxidant capacity** (167) | 1 |
|  |  | Harm | **Association of inflammatory marker with onset of JHR** (187) | 1 |  |  |
|  | Infection and infestation | Benefit | **Days of fever** (13,18–20,42,49,66,126,134,135,151,178,182,185,211,212,218,231,232) | 19 | **Rising antibody titre (prevention)** (233) | 1 |
|  |  |  | **Resolution of fever within specified time frame** (212) | 1 | **Decreasing antibody titre (prevention)** (233) | 1 |
|  |  |  | **Persistent fever** (212) | 1 | **Negative microscopic agglutination test (prevention)** (233) | 1 |
|  |  |  | **Clinical diagnosis of leptospirosis (prevention)** (15,141) | 2 | **Change in Leptospira antibody levels over time (prevention)** (234) | 1 |
|  |  |  | **Probable case of leptospirosis (prevention)** (141) | 1 | **Positive blood culture** (235) | 1 |
|  |  |  | **Confirmed case of leptospirosis (prevention)** (141) | 1 | **Positive urine culture** (19) | 1 |
|  |  |  | **Complication of Leptospirosis (general)** (6,19,121,236) | 4 | **Growth inhibition testing on serum** (237) | 1 |
|  |  |  | **Prevention of clinical manifestation of disease**  (15,36,124,131,135,227,235,238–242) | 12 | **No evolution in infection** (173) | 1 |
|  |  |  | **Prevention of clinical manifestation of disease confirmed by laboratory testing**  (15,124,131,235,238,240,241) | 7 | **Stable disease** (39) | 1 |
|  |  |  | **Prevention of seroconversion indicative of disease**  (124,131,233,235,238,239,242–244) | 9 | **Manifestation of severe disease**  (22,30,34,38,40,75,100,108,114,131,136,139,160,161,227,245,246) | 17 |
|  |  |  | **Baseline IgM remains zero (prevention)** (124) | 1 | **Attack rates** (39,131,136,233) | 4 |
|  |  |  | **Baseline IgM non-zero (prevention)** (124,233) | 2 | **Quantitative change in leptospiraemia** (247) | 1 |
|  |  |  | **Presence of antibodies to *Leptospira spp* (prevention)** (233,234,237,238) | 4 | **Persistent immune response to subsequent infection (prevention)** (31,248) | 2 |
|  |  |  | **Fourfold rise in antibody titre (prevention)** (233) | 1 | **Chronic post-leptospirosis symptoms** (249) | 1 |
|  | Musculo-skeletal and connective tissue | Benefit | **Symptoms of arthritis** (218) | 1 |  |  |
|  | Nervous system | Benefit | **Meningitis syndrome** (160,214,250–252) | 5 | **Encephalopathy** (25,26,44,77,253) | 5 |
|  |  |  | **Disease associated paralysis** (254) | 1 | **Seizure** (27,68,160,255) | 4 |
|  |  |  | **Improving CNS manifestations** (50,184,225,252,253,256–260) | 10 | **Central nervous system haemorrhagic manifestation** (27,63,152,261) | 4 |
|  |  |  | **Duration of headache** (42,185) | 2 |  |  |
|  | Pregnancy, puerperium, and perinatal | Benefit | **Low birth weight** (142,262) | 2 | **Term birth** (140) | 1 |
|  |  |  | **Fetal death** (140,142,262) | 3 | **Adverse infant/fetal outcomes (general)** (142,262) | 2 |
|  |  |  | **Neonatal death** (140,142,262) | 3 | **Admission to neonatal intensive care** (142) | 1 |
|  |  |  | **Live birth** (140,263) | 3 | **Median neonatal birth weight** (142) | 1 |
|  |  |  | **Preterm birth** (140,142,262,264) | 4 | **Median gestational age at delivery** (142) | 1 |
|  | Renal and urinary | Benefit | **Intradialysis MAP (mmHg)** (129) | 1 | **Stage of CKD (various criteria)** (265) | 1 |
|  |  |  | **Renal failure**  (17,22,24,27,39,44,45,61,63,67,74,78,80,89,104,110,123,126,133,135,148,157,162,163,175,188,189,193,213–215,228,230,245,251) | 35 | **Time to recovery of renal function**  (49,129,135,178,217,226,253,266) | 8 |
|  |  |  | **Acute kidney injury**  (3,30,95,98,151,267) | 6 | **Normalization of renal function**  (18,42,48,51,65,95,146,157,171,179,180,192,201,202,207,216,217,219,221–225,253,266,268–272) | 30 |
|  |  |  | **Oliguria** (18,126,188) | 3 | **MAKE***(30) | 1 |
|  |  |  | **Change in urine output** (21) | 1 | **Fluid Balance** (18,116) | 2 |
|  |  |  | **Biochemical measurements of renal function**  (21,30,121,146,173,211,226,265,266) | 9 | **Fluid intake (net/gross)** (129) | 1 |
|  |  |  | **Development of chronic kidney disease (CKD)**  (30,39,148,223,265) | 5 |  |  |
|  | Respiratory, thoracic, and mediastinal | Benefit | **Development of pulmonary haemorrhage**  (3,22,28,33,37,47,51,54–58,60,61,69,71,82,115,117,121,133,150,162,172,174,176,180,273) | 28 | **Evolution of lung pathology** (82) | 1 |
|  |  |  | **Respiratory distress**  (3,6,62,67,71,72,78,87,89,94,106,123,160,162,196,250,274) | 17 | **Respiratory complications** (82,118,162,214) | 4 |
|  |  |  | **Severity of respiratory distress** (106) | 1 | **Normalization of cardio-respiratory functions*** (179) | 1 |
|  |  |  | **Respiratory failure**  (17,33,49,56,147,151) | 6 | **Normalization of radiographic imaging** (51,56) | 2 |
|  |  |  | **P/F ratio** (106) | 1 |  |  |
| Life impact | Cognitive functioning | Benefit | **Knowledge/awareness of risk factors** (136) | 1 | **Behaviour related to risk factors** (136) | 1 |
|  | Global quality of life | Benefit | **Quality of life (non-specific)**  (15,16,40,173,184,256) | 6 |  |  |
|  | Delivery of care | Benefit | **Reduce outbreak events (prevention)** (136) | 1 | **Accessibility to healthcare** (136) | 1 |
|  |  |  | **Health system responsiveness/readiness for outbreaks** (136) | 1 | **Adherence to assigned intervention** (41) | 1 |
|  |  | Harm | **Development of antimicrobial resistance** (39,136) | 2 |  |  |
|  | Personal circumstances | Benefit | **Days lost from work for travel and medical attention** (41,232,243) | 3 | **Duration until return to normal work** (42,184) | 2 |
| Resource Use | Economic | Benefit | **Economic outcomes**  (42,131,136,232,243) | 5 | **Cost-effectiveness of treatment** (123) | 1 |
|  | Hospital | Benefit | **Days of clinical illness**  (94,126,130,141,185,236) | 6 | **ITU admission**  (1,2,6,7,12,14,17,70,77,96,97,109,111,113,116,118,121,127,129,149,152,162,164,166,172,174,189,191,194,195,197,204,216,227,250,251,273–280) | 44 |
|  |  |  | **Need for hospitalisation**  (6,126,141,232,239,243) | 6 | **Duration of ITU admission**  (1,12,14,17,109,111,118,127,129,166,194,204,273–275,278–280) | 18 |
|  |  |  | **Days of hospitalisation** (1,3,6,13,14,16,18,20,21,31,41–43,45,50,51,54,60,64,65,67,70,71,75,79,82,87,88,96,97,99,101,109,111–113,116,126,129,133–135,138,146,148,151,159,167,172,173,177,178,191,195,196,204,207,210,215,216,221–225,227,230,231,244,271,274,275,278–282) | 77 | **Need for hospitalisation attributable to leptospirosis** (141) | 1 |
|  |  |  | **Days of hospitalisation attributable to leptospirosis** (41) | 1 | **Complete medical discharge from hospital/clinic**  (33,39,45,46,50,68,101,123,138,159,168–170,172,177,179–181,183,184,186,189–193,197,201–203,206,207,209,210,213,215–217,219,220,222,224,225,228,231,232,244,253,256–259,261,263,264,268,270,272,275,276,278–280,282–297) | 79 |
|  | Need for further intervention | Benefit | **Duration of antibiotic use**  (6,50,65,111,115,151,190,220,292,298) | 10 | **Renal replacement free survival** (167) | 1 |
|  |  |  | **Need for mechanical ventilation**  (2,11,17,41,57,60,82,96,113,116,144,149,151,162,172,193,196,263) | 18 | **Number of dialysis sessions** (18,65,126,129) | 4 |
|  |  |  | **Duration of mechanical ventilation**  (11,12,14,31,41,82,115,127,129,156,158,176,196,221,231,273,274,280,298) | 19 | **Duration of dialysis**  (31,41,78,102,226,268,274) | 7 |
|  |  |  | **Need for non-invasive ventilation** (191) | 1 | **Ultrafiltration rate (ml/dialysis session)** (129) | 1 |
|  |  |  | **Need for high-flow nasal oxygen** (276) | 1 | **Need for haemofiltriation** (116) | 1 |
|  |  |  | **Need for ECMO** (172) | 1 | **MAKE***(30) | 1 |
|  |  |  | **Duration of ECMO** (31,273) | 2 | **Need for blood product transfusion**  (3,57,70,75,82,96,163,172,174) | 9 |
|  |  |  | **Need for ionotropic support**  (3,65,88,113,116,149,162,172,189,250,276) | 11 | **Treatment failure** (39,173,212) | 3 |
|  |  |  | **Duration of inotropic support** (65,268) | 2 | **Partial treatment response** (39) | 1 |
|  |  |  | **Need for dialysis**  (18,30,39,41,65,67,75,78,79,96,97,102,110,112,113,115,116,126,146,148,162,174,191,213,223,251,289,297) | 28 | **Need for subsequent antimicrobial** (20) | 1 |
| Adverse events | Adverse events/  effects | Harm | **Manifestation of Jarisch–Herxheimer reaction (JHR)**  (32,49,102,159,162,173,180,187,199,287,299) | 11 | **Non-serious adverse events** (15,16,41,124) | 4 |
|  |  |  | **JHR manifestation association by antibiotic type** (187) | 1 | **Serious adverse events** (15,16,41,173) | 4 |
|  |  |  | **Manifestation of nosocomial infection**  (12,14,20,31,82,154) | 6 | **Safety (general)** (159,167) | 2 |
|  |  |  | **Drug safety** (41,125,136) | 3 | **Adverse events/side effects (non-specified)**  (13,14,20,21,41,42,82,125,126,141,173,212,232,240–242) | 16 |
|  | | | | | | |

## Full Reference List of Included Studies

1. Allyn J, Miailhe AF, Delmas B, Marti L, Allou N, Jabot J, et al. Severe leptospirosis in tropical and non-tropical areas: A comparison of two french, multicentre, retrospective cohorts. PLoS Negl Trop Dis [Internet]. 2024;18(4):e0012084–e0012084. Available from: https://www.ncbi.nlm.nih.gov/pmc/articles/PMC11034666https://dx.doi.org/10.1371/journal.pntd.0012084

2. Cogliano F, Sadonio MJ, Cravero G, Correa A, Castro MG, Galluccio FR. Leptospirosis: predictores de mala evolución clínica en pacientes hospitalizados, 25 años de experiencia. Actual Sida Infectol (En linea) [Internet]. 2024;32(114):36–45. Available from: https://doi.org/10.52226/revista.v32i114.279

3. Pinto GV, Senthilkumar K, Rai P, Kabekkodu SP, Karunasagar I, Kumar BK. Identification of Dominant Leptospira Serogroups among Leptospirosis Cases and Their Clinical Outcomes: A Prospective Hospital-Based Study in Mangaluru, India. American Journal of Tropical Medicine and Hygiene. 2024 Jun 1;110(6):1230–6.

4. Silva AF da, Figueiredo K, Falcão IWS, Costa FAR, da Rocha Seruffo MC, de Moraes CCG. Study of machine learning techniques for outcome assessment of leptospirosis patients. Sci Rep. 2024 Dec 1;14(1).

5. Parra-Barrera EL, Bello-Piruccini S, Rodríguez K, Duarte-Valderrama C, Torres M, Undurraga EA. Serologically Confirmed Human Leptospirosis in Colombia, 2015-2020. Am J Trop Med Hyg [Internet]. 2024;111(4):856–64. Available from: https://www.ncbi.nlm.nih.gov/pmc/articles/PMC11448516https://dx.doi.org/10.4269/ajtmh.23-0654

6. Uribe-Restrepo P, Perez-Garcia J, Arboleda M, Munoz-Zanzi C, Agudelo-Florez P. Clinical presentation of human leptospirosis in febrile patients: Urabá, Colombia. PLoS Negl Trop Dis. 2024 Sep 1;18(9):e0012449.

7. Gurung S, Tewari D, Sherpa U, Chhophel TP, Siddique AI, Sarmah N, et al. Fatal case of Leptospira wolffii infection in Sikkim, India: An autochthonous case from a new geographical region. Indian J Med Microbiol. 2025 Mar 1;54.

8. Dewi IP, Bagaskara AT, Anggitama AM, Damayanti KRS, Mukti I. Total atrioventricular block as a cardiac manifestation in Weil’s disease: a case report. Journal of Medical Case Reports . 2025 Dec 1;19(1).

9. Marotto LS, Marotto MS, Barrese TZ, Borges CDSC, Guerra JM, Araújo LJT de, et al. Leptospirosis and coinfections leading to fatal multiple organ and system failure. Rev Inst Med Trop Sao Paulo [Internet]. 2025;67:e26–e26. Available from: https://www.ncbi.nlm.nih.gov/pmc/articles/PMC11996032https://dx.doi.org/10.1590/S1678-9946202567026http://www.scielo.br/scielo.php?script=sci_arttext&nrm=iso&lng=pt&tlng=pt&pid=S0036-46652025000100507

10. Ranieri TM, Viegas da Silva E, Vallandro MJ, Oliveira MM de, Barcellos RB, Lenhardt RV, et al. Leptospirosis Cases During the 2024 Catastrophic Flood in Rio Grande Do Sul, Brazil. Pathogens. 2025 Apr 1;14(4).

11. Petakh P, Oksenych V, Kamyshnyi O. Corticosteroid Treatment for Leptospirosis: A Systematic Review and Meta-Analysis. J Clin Med [Internet]. 2024;13(15):4310. Available from: https://www.mdpi.com/journal/jcmhttps://discover.lshtm.ac.uk/openurl/44HYG/44HYG_services_page?sid=OVID:embase&id=doi:10.3390%2Fjcm13154310&id=pmid&issn=2077-0383&isbn=&volume=13&issue=15&spage=4310&pages=&date=2024&title=Journal+of+Clinical+Medicine&atit

12. Ruwanpathirana P, Perera N, Rambukwella R, Priyankara D. Clinical characteristics and outcomes of pulmonary haemorrhage in leptospirosis: A retrospective cohort study from Sri Lanka. medRxiv [Internet]. 2024;((Ruwanpathirana) Professorial Unit in Medicine, National Hospital of Sri Lanka, Colombo, Sri Lanka(Perera) Department of Medicine, Faculty of Medical Sciences, University of Sri Jayewardenepura, Nugegoda, Sri Lanka(Rambukwella) Provincial Director of Heal). Available from: https://www.medrxiv.org/https://discover.lshtm.ac.uk/openurl/44HYG/44HYG_services_page?sid=OVID:embase&id=doi:10.1101%2F2024.03.25.24304826&id=pmid&issn=&isbn=&volume=&issue=&spage=&pages=&date=2024&title=medRxiv&atitle=Clinical+characteristics+and+outcom

13. Ji Z, Jian M, Su X, Pan Y, Duan Y, Ma W, et al. Efficacy and safety of antibiotics for treatment of leptospirosis: a systematic review and network meta-analysis. Syst Rev [Internet]. 2024;13(1):108. Available from: https://discover.lshtm.ac.uk/openurl/44HYG/44HYG_services_page?sid=OVID:medline&id=doi:10.1186%2Fs13643-024-02519-y&id=pmid38627798&issn=2046-4053&isbn=&volume=13&issue=1&spage=108&pages=108&date=2024&title=Systematic+Reviews&atitle=Efficacy+and+safety+of

14. SLCTR/2024/037. The effect of moderate-dose and high-dose intravenous methylprednisolone combined with plasmapheresis versus plasmapheresis alone in improving clinical outcome of patients with leptospirosis pulmonary haemorrhage syndrome. 2024; Available from: https://www.cochranelibrary.com/central/doi/10.1002/central/CN-02786660/full

15. Win TZ, Perinpanathan T, Mukadi P, Smith C, Edwards T, Han SM, et al. Antibiotic prophylaxis for leptospirosis. Cochrane Database of Systematic Reviews [Internet]. 2024 Mar 14;2024(3). Available from: http://doi.wiley.com/10.1002/14651858.CD014959.pub2

16. Win TZ, Han SM, Edwards T, Maung HT, Brett-Major DM, Smith C, et al. Antibiotics for treatment of leptospirosis. Cochrane Database of Systematic Reviews [Internet]. 2024 Mar 14;2024(3). Available from: http://doi.wiley.com/10.1002/14651858.CD014960.pub2

17. Alian S, Asghari H, Najafi N, Davoudi A, Yazdani J. Corticosteroid in the Treatment of Moderate to Severe Thrombocytopenia Due to Leptospirosis. Iran Red Crescent Med J [Internet]. 2014 Oct 5;16(10):e16030. Available from: NS  - Alian 2014

18. De Francesco Daher E, Nogueira CB. Evaluation of penicillin therapy in patients with leptospirosis and acute renal failure. Rev Inst Med Trop Sao Paulo. 2000;42(6):327–32.

19. Edwards CN, Nicholson GD, Hassell TA, Everard COR, Callender J. Penicillin therapy in icteric leptospirosis. American Journal of Tropical Medicine and Hygiene. 1988;39(4):388–90.

20. Suputtamongkol Y, Niwattayakul K, Suttinont C, Losuwanaluk K, Limpaiboon R, Chierakul W, et al. An open, randomized, controlled trial of penicillin, doxycycline, and cefotaxime for patients with severe leptospirosis. Clinical Infectious Diseases. 2004;39(10):1417–24.

21. de Cerqueira MC. Pulsoterapia com metilprednisolona e evolucao nefropatia da leptospirose humana. Salvador; 1985 Feb.

22. Yersin C, Bovet P, Mérien F, Wong T, Panowsky J, Perolat P. Human leptospirosis in the Seychelles (Indian Ocean): a population-based study. Am J Trop Med Hyg [Internet]. 1998 Dec 1;59(6):933–40. Available from: http://www.ncbi.nlm.nih.gov/pubmed/9886203

23. Suzuki K, Nakamura S, Watanabe H. A fatal case of Leptospira autumnalis infection in Lao PDR. Southeast Asian J Trop Med Public Health [Internet]. 1997 Jun;28(2):436–7. Available from: http://www.ncbi.nlm.nih.gov/pubmed/9444037

24. Lepilleur B, Zohir AH. About a case of massive pulmonary haemorrhage due to Leptospira icterohaemorrhagiae with a fatal evolution. Ann Biol Clin (Paris) [Internet]. 2000;58(5):624–6. Available from: http://www.ncbi.nlm.nih.gov/pubmed/11022109

25. Amaya-Villar R, Garnacho-Montero J, Jiménez-Jiménez FJ, García-Garmendia JL, Ortíz-Leyba C. Fulminant leptospirosis in a previously healthy man. Intensive Care Med [Internet]. 2001 Mar 22;27(3):616–616. Available from: http://link.springer.com/10.1007/s001340000844

26. Dimopoulou I, Politis P, Panagyiotakopoulos G, Moulopoulos L, Theodorakopoulou M, Bisirtzoglou D, et al. Leptospirosis presenting with encephalitis-induced coma. Intensive Care Med [Internet]. 2002 Nov 1;28(11):1682–1682. Available from: http://link.springer.com/10.1007/s00134-002-1476-2

27. Theilen HJ, Lück C, Hanisch U, Ragaller M. Fatal intracerebral hemorrhage due to leptospirosis. Infection. 2002;30(2):109–12.

28. Divate SA, Chaturvedi R, Jadhav NN, Vaideeswar P. Leptospirosis associated with diffuse alveolar haemorrhage. J Postgrad Med [Internet]. 2002;48(2):131–2. Available from: http://www.ncbi.nlm.nih.gov/pubmed/12215699

29. Sainaresh V V, Sriramnaveen P, Sivaramakrishna G, Vijayalakshmi DB, Lakshmi AY, Sivakumar V. Subcapsular hematoma of liver in a patient of Weil’s disease. Trop Gastroenterol [Internet]. 2011;32(2):133–4. Available from: http://www.ncbi.nlm.nih.gov/pubmed/21922878

30. Phannajit J, Lertussavavivat T, Limothai U, Tachaboon S, Avihingsanon Y, Praditpornsilpa K, et al. Long-term kidney outcomes after leptospirosis: a prospective multicentre cohort study in Thailand. Nephrology Dialysis Transplantation. 2023 Oct 1;38(10):2182–91.

31. Madrigal TPR, Panlilio MTT, Burog AILD, Danguilan RA, Chavez JR. Incidence of acute pancreatitis among patients with leptospirosis requiring extracorporeal membrane oxygenation (ECMO): a descriptive study. BMJ Open Gastroenterol. 2023 Mar 16;10(1).

32. Chiko Y, Shiokawa K, Namihira I, Itagaki K, Maruyama K, Tachibana Y, et al. Report of Weil’s disease with a fatal course triggered by Jarisch-Herxheimer reaction. Journal of Infection and Chemotherapy. 2023 Aug 1;29(8):800–2.

33. Charles JC, Jayarajah U, Subasinghe D. Clinical characteristics and outcomes of patients with leptospirosis complicated with acute pancreatitis: a systematic review. Vol. 51, Journal of International Medical Research. SAGE Publications Ltd; 2023.

34. Widawati M, Dhewantara PW, Anasi R, Wahono T, Marina R, Pertiwi IP, et al. An investigation of geographical clusters of leptospirosis during the outbreak in Pangandaran, West Java, Indonesia. Geospatial Health (Testo stamp) [Internet]. 2023;18(2). Available from: https://dx.doi.org/10.4081/gh.2023.1221

35. Barrera ELP, Reales-González J, Salas D, Santamaría ER, Bello S, Rico A, et al. Fatal acute undifferentiated febrile illness among clinically suspected leptospirosis cases in Colombia, 2016–2019. PLoS Negl Trop Dis. 2023 Oct 1;17(10 October).

36. Gupta N, Wilson W, Ravindra P. Leptospirosis in India: a systematic review and meta-analysis of clinical profile, treatment and outcomes. Infezioni in Medicina [Internet]. 2023;31(3):290–305. Available from: https://www.infezmed.it/media/journal/Vol_31_3_2023_4.pdf

37. Petakh P, Rostoka L, Isevych V, Kamyshnyi A. Identifying risk factors and disease severity in leptospirosis: A meta-analysis of clinical predictors. Trop Doct. 2023;53(4):464–9.

38. Maillard O. NCT04034550 - Cohort of Hospitalized Patients Suspected of Leptospirosis (COLEPT) [Internet]. 2019 [cited 2024 May 26]. Available from: https://clinicaltrials.gov/study/NCT04034550

39. Köhler F. NCT04288674 - Leptospirosis Registry - LeptoScope (Leptoscope) [Internet]. 2022 [cited 2024 May 26]. Available from: https://classic.clinicaltrials.gov/ct2/show/NCT04288674

40. Hochedez P. French West Indies Leptospirosis Study (LEPTO). 2010 [cited 2023 Nov 23]; Available from: https://classic.clinicaltrials.gov/show/NCT01607047

41. Brett-Major DM, Coldren R. Antibiotics for leptospirosis. Cochrane Database Syst Rev [Internet]. 2012;2(2):CD008264. Available from: http://www.ncbi.nlm.nih.gov/pubmed/22336839

42. Oberai P. Homoeopathy as as add on treatment for severe Leptospirosis patients on conventional care (CTRI/2012/01/002316) [Internet]. Clinical Trials Registry - India. 2012 [cited 2024 Mar 6]. Available from: https://trialsearch.who.int/Trial2.aspx?TrialID=CTRI/2012/01/002316

43. Maze SS, Kirsch RE. Leptospirosis experience at Groote Schuur Hospital, 1969-1979. S Afr Med J [Internet]. 1981 Jan 10;59(2):33–6. Available from: http://www.ncbi.nlm.nih.gov/pubmed/7455830

44. Raoult D, Jeandel P, Mailloux M, Rougier Y. Thrombocytopenia and renal failure in leptospirosis. Am J Trop Med Hyg [Internet]. 1983 Nov;32(6):1464. Available from: http://www.ncbi.nlm.nih.gov/pubmed/6650748

45. Lecour H, Miranda M, Magro C, Gonçalves V, Rocha A. Human leptospirosis — a review of 50 cases. Infection [Internet]. 1989 Jan;17(1):8–12. Available from: http://link.springer.com/10.1007/BF01643489

46. Baridó ME, Alexanderson E, Halabe J, Castro G, Caballero A. [Weil’s disease: report on 5 cases in the valley of Mexico]. Rev Invest Clin [Internet]. 1989;41(3):253–7. Available from: http://www.ncbi.nlm.nih.gov/pubmed/2814000

47. Gonçalves AJR, Carvalho JEM de, Silva JBG e, Rozembaum R, Vieira ARM. Hemoptise/s e síndrome de angústia respiratória do adulto como causas de morte na leptospirose: mudanças de padrões clínicos e anatomopatológicos. Rev Soc Bras Med Trop [Internet]. 1992 Dec;25(4):261–70. Available from: http://www.scielo.br/scielo.php?script=sci_arttext&pid=S0037-86821992000400009&lng=pt&tlng=pt

48. Neves E de S, Pereira MM, Galhardo MCG, Caroli A, Andrade J, Morgado MG, et al. Leptospirosis patient with AIDS the first case reported. Rev Soc Bras Med Trop [Internet]. 1994 Mar;27(1):39–42. Available from: http://www.scielo.br/scielo.php?script=sci_arttext&pid=S0037-86821994000100008&lng=en&tlng=en

49. Marotto PCF, Marotto MS, Santos DL, Souza TNL, Seguro AC. Outcome of Leptospirosis in Children. Am J Trop Med Hyg [Internet]. 1997 Mar 1;56(3):307–10. Available from: https://www.ajtmh.org/view/journals/tpmd/56/3/article-p307.xml

50. Yang CW, Pan MJ, Wu MS, Chen YM, Tsen YT, Lin CL, et al. Leptospirosis: An ignored cause of acute renal failure in Taiwan. American Journal of Kidney Diseases [Internet]. 1997 Dec;30(6):840–5. Available from: https://linkinghub.elsevier.com/retrieve/pii/S0272638697900913

51. Simpson FG, Green KA, Haug GJ, Brookes DL. Leptospirosis associated with severe pulmonary haemorrhage in Far North Queensland. Medical Journal of Australia. 1998 Aug 3;169(3):151–3.

52. Schillinger F, Babeau N, Montagnac R, Milcent T. [Severe renal forms of leptospirosis. Apropos of 6 cases seen in 15 years at one center]. Nephrologie [Internet]. 1999;20(2):81–6. Available from: https://search.bvsalud.org/portal/resource/en/mdl-10227028

53. Sanders EJ, Rigau-Pérez JG, Spiegel RA, Deseda CC, Smits HL, Weyant RS, et al. Increase of leptospirosis in dengue-negative patients after a hurricane in Puerto Rico in 1996 [correction of 1966]. Am J Trop Med Hyg [Internet]. 1999 Sep 1;61(3):399–404. Available from: https://ajtmh.org/doi/10.4269/ajtmh.1999.61.399

54. Yersin C, Bovet P, Mérien F, Clément J, Laille M, Van Ranst M, et al. Pulmonary haemorrhage as a predominant cause of death in leptospirosis in Seychelles. Trans R Soc Trop Med Hyg [Internet]. 2000 Jan;94(1):71–6. Available from: https://academic.oup.com/trstmh/article-lookup/doi/10.1016/S0035-9203(00)90445-0

55. Sánchez Rubio P. Cuidar a un paciente con leptospirosis icterohemorrágica. Enferm Intensiva [Internet]. 2000 Sep;11(1):17–22. Available from: http://www.ncbi.nlm.nih.gov/pubmed/10889614

56. Trivedi S V, Chavda RK, Wadia PZ, Sheth V, Bhagade PN, Trivedi SP, et al. The role of glucocorticoid pulse therapy in pulmonary involvement in leptospirosis. J Assoc Physicians India [Internet]. 2001 Sep;49:901–3. Available from: http://www.ncbi.nlm.nih.gov/pubmed/11837758

57. Akiyama K, Ueki Y, Okimura Y, Goto I, Shiraishi H. A Fatal Case of Weil’s Disease in Miyagi Prefecture. Jpn J Infect Dis. 2001;54:156–7.

58. Seijo A, Coto H, San Juan J, Videla J, Deodato B, Cernigoi B, et al. Dístrés respiratorio debido a hemorragia pulmonar por leptospirosis. Medicina (BAires) [Internet]. 2002;62(2):135–40. Available from: https://search.bvsalud.org/portal/resource/en/biblio-1165116

59. R Vieira SR, Brauner JS, Regina Rios Vieira Rua S. Leptospirosis as a Cause of Acute Respiratory Failure: Clinical Features and Outcome in 35 Critical Care Patients. The Brazilian Journal of Infectious Diseases [Internet]. 2002;6(3):135–9. Available from: www.infecto.org.br/bjid.htm

60. Silva JJP da, Dalston MO, Carvalho JEM de, Setúbal S, Oliveira JMC de, Pereira MM. Clinicopathological and immunohistochemical featuresof the severe pulmonary form of leptospirosis. Rev Soc Bras Med Trop [Internet]. 2002 Aug;35(4):395–9. Available from: http://www.scielo.br/scielo.php?script=sci_arttext&pid=S0037-86822002000400017&lng=en&tlng=en

61. Niwattayakul K, Homvijitkul J, Niwattayakul S, Khow O, Sitprija V. Hypotension, renal failure, and pulmonary complications in leptospirosis. Ren Fail. 2002;24(3):297–305.

62. Kishor KK, Rao P V, Bhat KR, Shastry BA. Pancreatitis in Weil’s Disease. Trop Doct [Internet]. 2002 Oct 25;32(4):230–1. Available from: http://journals.sagepub.com/doi/10.1177/004947550203200416

63. Christova I, Tasseva E, Manev H. Human leptospirosis in Bulgaria, 1989-2001: Epidemiological, clinical, and serological features. Scand J Infect Dis. 2003;35(11–12):869–72.

64. Moranne O, Queyrel V. Leptospirosis in Espiritu Santo, Vanuatu, 8 case reports. Med Trop (Mars) [Internet]. 2003;63(6):611–3. Available from: http://www.ncbi.nlm.nih.gov/pubmed/15077426

65. Covic A, Goldsmith DJA, Gusbeth-Tatomir P, Seica A, Covic M. A retrospective 5-year study in Moldova of acute renal failure due to leptospirosis: 58 cases and a review of the literature. Nephrol Dial Transplant [Internet]. 2003;18(6):1128–34. Available from: https://search.bvsalud.org/portal/resource/en/mdl-12748345

66. Panaphut T, Domrongkitchaiporn S, Vibhagool A, Thinkamrop B, Susaengrat W. Ceftriaxone compared with sodium penicillin g for treatment of severe leptospirosis. Clin Infect Dis [Internet]. 2003 Jun 15;36(12):1507–13. Available from: http://www.ncbi.nlm.nih.gov/pubmed/12802748

67. Costa E, Lopes AA, Sacramento E, Costa YA, Matos ED, Lopes MB, et al. Penicillin at the late stage of leptospirosis: A randomized controlled trial. Rev Inst Med Trop Sao Paulo. 2003;45(3):141–5.

68. Verma B, Daga SR, Sawant D. Leptospirosis in children. Indian Pediatr [Internet]. 2003 Nov;40(11):1081–3. Available from: http://www.ncbi.nlm.nih.gov/pubmed/14660841

69. Yang GG, Hsu YH. Nitric oxide production and immunoglobulin deposition in leptospiral hemorrhagic respiratory failure. Journal of the Formosan Medical Association [Internet]. 2005 Oct;104(10):759–63. Available from: http://www.ncbi.nlm.nih.gov/pubmed/16385381

70. Erdinc FS, Koruk ST, Hatipoglu CA, Kinikli S, Demiroz AP. Three cases of anicteric leptospirosis from Turkey: Mild to severe complications. Journal of Infection. 2006 Feb;52(2).

71. Turhan V, Atasoyu EM, Kucukardali Y, Polat E, Cesur T, Cavuslu S. Case report. Leptospirosis presenting as severe rhabdomyolysis and pulmonary haemorrhage. Journal of Infection. 2006 Jan;52(1).

72. Spichler A, Spichler E, Moock M, Vinetz JM, Leake JAD. Acute pancreatitis in fatal anicteric leptospirosis. American Journal of Tropical Medicine and Hygiene. 2007;76(5):886–7.

73. Manciuc C, Dorobăţ C, Hurmuzache M, Luca V, Mihalache D, Grigorescu O. [Leptospirosis in children. Clinico-biological and therapeutic aspects for the cases hospitalised between 1992-2005]. Rev Med Chir Soc Med Nat Iasi [Internet]. 2007;111(2):383–5. Available from: http://www.ncbi.nlm.nih.gov/pubmed/17983172

74. Ittyachen AM, Krishnapillai T V., Nair MC, Rajan AR. Retrospective study of severe cases of leptospirosis admitted in the intensive care unit. J Postgrad Med. 2007 Oct 1;53(4):232–5.

75. Tovîrnac M, Manole A, Manole M, Trifan M. Retroprospective clinical epidemiological research on leptospirosis cases hospitalised in the Infectious Diseases Clinic of Iaşi. Rev Med Chir Soc Med Nat Iasi [Internet]. 2008;112(2):483–8. Available from: http://www.ncbi.nlm.nih.gov/pubmed/19295024

76. Liverpool J, Francis S, Liverpool CE, Dean GT, Mendez DD. Leptospirosis: Case reports of an outbreak in Guyana. Ann Trop Med Parasitol. 2008 Apr;102(3):239–45.

77. Alves AP, Moura DCS, Spolti GP. Co-infection with hepatitis A and leptospirosis in the Amazon region: report of two cases. Tropical gastroenterology [Internet]. 2011;32(3):234–6. Available from: http://www.ncbi.nlm.nih.gov/pubmed/22332345

78. Datta S, Sarkar RN, Biswas A, Mitra S. Leptospirosis: an institutional experience. J Indian Med Assoc [Internet]. 2011 Oct;109(10):737–8. Available from: http://www.ncbi.nlm.nih.gov/pubmed/22482321

79. Silva Júnior GB, Abreu KLS, Mota RM, Barreto AG, Araújo SM, Rocha H Al, et al. RIFLE and Acute Kidney Injury Network classifications predict mortality in leptospirosis-associated acute kidney injury. Nephrology. 2011 Mar;16(3):269–76.

80. Echeverri LM, Atehortúa S, Ospina S. Leptospirosis con inmunoglobulina M positiva en pacientes hospitalizados en una institución de tercer nivel de Medellín, Colombia, en 2009. Infectio [Internet]. 2011 Jun;15(2):118–23. Available from: http://linkinghub.elsevier.com/retrieve/pii/S0123939211707512

81. Deodhar D, John M. Leptospirosis: experience at a tertiary care hospital in northern India. Natl Med J India [Internet]. 2011;24(2):78–80. Available from: http://www.ncbi.nlm.nih.gov/pubmed/21668048

82. Azevedo AFC, de B Miranda-Filho D, Henriques-Filho GT, Leite A, Ximenes RAA. Randomized controlled trial of pulse methyl prednisolone × placebo in treatment of pulmonary involvement associated with severe leptospirosis. [ISRCTN74625030]. BMC Infect Dis. 2011 Jun 30;11.

83. Lo YC, Kintziger KW, Carson HJ, Patrick SL, Turabelidze G, Stanek D, et al. Severe Leptospirosis Similar to Pandemic (H1N1) 2009, Florida and Missouri, USA. Emerg Infect Dis. 2011 Jun;17(6):1145–6.

84. Montero-Tinnirello J, de la Fuente-Aguado J, Ochoa-Diez M, Cabadas-Avión R. Hemorragia pulmonar por leptospirosis. Vol. 36, Medicina Intensiva. 2012. p. 58–9.

85. Spichler A, Athanazio DA, Vilaça P, Seguro A, Vinetz J, Leake JAD. Comparative analysis of severe pediatric and adult leptospirosis in São Paulo, Brazil. American Journal of Tropical Medicine and Hygiene. 2012 Feb;86(2):306–8.

86. Sharp TM, Bracero J, Rivera A, Shieh WJ, Bhatnagar J, Rivera-Diez I, et al. Fatal human co-infection with Leptospira spp. and dengue virus, Puerto Rico, 2010. Emerg Infect Dis [Internet]. 2012 May;18(5):878–80. Available from: http://www.ncbi.nlm.nih.gov/pubmed/22516301

87. Hin HS, Ramalingam R, Chunn KY, Ahmad N, Ab Rahman J, Mohamed MS. Case report: Fatal co-infection-melioidosis and leptospirosis. American Journal of Tropical Medicine and Hygiene. 2012 Oct;87(4):737–40.

88. Gancheva G, Karcheva M. Icterohaemorrhagic leptospirosis in patients with history of alcohol abuse - Report of two cases. Turkish Journal of Gastroenterology. 2013;24(6):549–55.

89. Cudós MC, Landolt N, Jacob P, Schmeling MF, Chiani Y, Brazza S, et al. Vigilancia intensificada de leptospirosis en Santa Fe y Entre Ríos (2012-2013). Rev Argent Salud Publica. 2014;5(18):24–30.

90. Rodríguez-Vidigal FF, Vera-Tomé A, Nogales-Muñoz N, Muñoz-García-Borruel M, Muñoz-Sanz A. Leptospirosis en un área sanitaria del suroeste español. Rev Clin Esp. 2014;214(5):247–52.

91. Pérez Rodríguez NM, Galloway R, Blau DM, Traxler R, Bhatnagar J, Zaki SR, et al. Case report: Case series of fatal Leptospira spp./dengue virus co-infections - Puerto Rico, 2010-2012. American Journal of Tropical Medicine and Hygiene. 2014 Oct 1;91(4):760–5.

92. Pushpakumara J, Prasath T, Samarajiwa G, Priyadarshani S, Perera N, Indrakumar J. Myocarditis causing severe heart failure - An unusual early manifestation of leptospirosis: A case report Infectious Diseases. BMC Res Notes. 2015;8(1).

93. Ramirez-Ramirez M, Leon-Castaneda O, Rodriguez-Morales A. Leptospirosis in an Urban Setting: Cases Diagnosed at a Private Medical Center of Western Colombia, 2008-2012. Recent Pat Antiinfect Drug Discov [Internet]. 2015 May 21;10(1):59–63. Available from: http://www.eurekaselect.com/openurl/content.php?genre=article&issn=1574-891X&volume=10&issue=1&spage=59

94. Wijesinghe A, Gnanapragash N, Ranasinghe G, Ragunathan MK. Fatal co-infection with leptospirosis and dengue in a Sri Lankan male. BMC Res Notes. 2015 Aug 13;8(1).

95. Srisawat N, Praditpornsilpa K, Patarakul K, Techapornrung M, Daraswang T, Sukmark T, et al. Neutrophil gelatinase associated lipocalin (NGAL) in leptospirosis acute kidney injury: A multicenter study in Thailand. PLoS One. 2015 Dec 1;10(12).

96. Sharp TM, Rivera García B, Pérez-Padilla J, Galloway RL, Guerra M, Ryff KR, et al. Early Indicators of Fatal Leptospirosis during the 2010 Epidemic in Puerto Rico. PLoS Negl Trop Dis. 2016 Feb 25;10(2).

97. Fernando N, Wickremesinghe S, Niloofa R, Rodrigo C, Karunanayake L, De Silva HJ, et al. Protein carbonyl as a biomarker of oxidative stress in severe leptospirosis, and its usefulness in differentiating leptospirosis from dengue infections. PLoS One. 2016 Jun 1;11(6).

98. Teles F, Uchôa JV de M, Mendonça DMB, Costa AFP. Acute kidney injury in leptospirosis: The Kidney Disease Improving Global Outcomes (KDIGO) criteria and mortality. Clin Nephrol. 2016;86(6):303–9.

99. Raffray L, Giry C, Vandroux D, Kuli B, Randrianjohany A, Pequin AM, et al. Major neutrophilia observed in acute phase of human leptospirosis is not associated with increased expression of granulocyte cell activation markers. PLoS One. 2016 Nov 1;11(11).

100. Lee N, Kitashoji E, Koizumi N, Lacuesta TL V., Ribo MR, Dimaano EM, et al. Building prognostic models for adverse outcomes in a prospective cohort of hospitalised patients with acute leptospirosis infection in the Philippines. Trans R Soc Trop Med Hyg. 2017 Dec 1;111(12):531–9.

101. Mutoh Y, Koizumi N, Morino E, Hayakawa K, Kato Y, Ohmagari N. Leptospirosis Cases in the Tokyo Metropolitan Area, Japan. Jpn J Infect Dis [Internet]. 2017 Nov 22;70(6):669–71. Available from: http://www.ncbi.nlm.nih.gov/pubmed/28890513

102. Kupferman T, Coffee MP, Eckhardt BJ. Case report: A cluster of three leptospirosis cases in a New York City abattoir and an unusual complication in the index case. American Journal of Tropical Medicine and Hygiene. 2017;97(6):1679–81.

103. Puca E, Pipero P, Harxhi A, Abazaj E, Gega A, Puca E, et al. The role of gender in the prevalence of human leptospirosis in Albania. J Infect Dev Ctries. 2018 Mar 1;12(3):150–5.

104. Torres Vargas C, Martínez Herreros Á, Sacristán Terroba B. Weil’s disease and acute multifactorial hepatitis. About a case. Gastroenterol Hepatol. 2018 Apr 1;41(4):253–4.

105. Tan TL, Lee LY, Lim WC. Fatal Leptospirosis and Escherichia coli co-infection in a post-partum woman. Med J Malaysia [Internet]. 2018 Dec;73(6):427–9. Available from: http://www.ncbi.nlm.nih.gov/pubmed/30647223

106. Kumar SS, Selvarajan Chettiar KP, Nambiar R. Etiology and Outcomes of ARDS in a Resource Limited Urban Tropical Setting. J Natl Med Assoc. 2018 Aug 1;110(4):352–7.

107. Rahimi R, Omar E, Tuan Soh TS, Mohd Nawi SFA, Md Noor S. Leptospirosis in pregnancy: A lesson in subtlety. Malays J Pathol [Internet]. 2018 Aug;40(2):169–73. Available from: http://www.ncbi.nlm.nih.gov/pubmed/30173235

108. Smith S, Kennedy BJ, Dermedgoglou A, Poulgrain SS, Paavola MP, Minto TL, et al. A simple score to predict severe leptospirosis. PLoS Negl Trop Dis. 2019 Feb 1;13(2).

109. Vandroux D, Chanareille P, Delmas B, Gaüzère BA, Allou N, Raffray L, et al. Acute respiratory distress syndrome in leptospirosis. J Crit Care. 2019 Jun 1;51:165–9.

110. Daher EDF, Soares D de S, Galdino GS, Macedo ÊS, Gomes PEA de C, Pires Neto R da J, et al. Leptospirosis in the elderly: the role of age as a predictor of poor outcomes in hospitalized patients. Pathog Glob Health. 2019 Apr 3;113(3):117–23.

111. Miailhe AF, Mercier E, Maamar A, Lacherade JC, Le Thuaut A, Gaultier A, et al. Severe leptospirosis in non-tropical areas: a nationwide, multicentre, retrospective study in French ICUs. Intensive Care Med. 2019 Dec 1;45(12):1763–73.

112. Gomes PEA de C, Brilhante S de O, Carvalho RB, de Sousa DR, Daher EDF. Pancreatitis as a severe complication of leptospirosis with fatal outcome: A case report. Rev Inst Med Trop Sao Paulo. 2019;61.

113. Fann RJ, Vidya RR, Chong HE, Indralingam V, Christopher Chan WS. Clinical presentations and predictors of mortality for leptospirosis - A study from suburban area in Malaysia. Med J Malaysia [Internet]. 2020 Jan;75(1):52–6. Available from: http://www.ncbi.nlm.nih.gov/pubmed/32008021

114. Wan Yusoff WSY, Abdullah M, Sekawi Z, Amran F, Yuhana MY, Mohd Taib N, et al. Elevated levels of IL-8 in fatal leptospirosis. Pathog Glob Health. 2020 Feb 17;114(2):99–103.

115. Gkentzi D, Lagadinou M, Bountouris P, Dimitrakopoulos O, Triantos C, Marangos M, et al. Epidemiology, clinical and laboratory findings of leptospirosis in Southwestern Greece. Infect Dis. 2020 Jun 2;52(6):413–8.

116. Ajjimarungsi A, Bhurayanontachai R, Chusri S. Clinical characteristics, outcomes, and predictors of leptospirosis in patients admitted to the medical intensive care unit: A retrospective analysis. J Infect Public Health. 2020 Dec 1;13(12):2055–61.

117. Nicodemo AC, Duarte-Neto AN. Pathogenesis of Pulmonary Hemorrhagic Syndrome in Human Leptospirosis. Vol. 104, American Journal of Tropical Medicine and Hygiene. American Society of Tropical Medicine and Hygiene; 2021. p. 1970–2.

118. Ramírez-García R, Quintero JC, Rosado AP, Arboleda M, González VA, Agudelo-Flórez P. Leptospirosis and rickettsiosis, a diagnostic challenge for febrile syndrome in endemic areas. Biomedica [Internet]. 2021;41(2):208–17. Available from: https://www.ncbi.nlm.nih.gov/pmc/articles/PMC8372841https://dx.doi.org/10.7705/biomedica.5598

119. Philip N, Lung Than LT, Shah AM, Yuhana MY, Sekawi Z, Neela VK. Predictors of severe leptospirosis: a multicentre observational study from Central Malaysia. BMC Infect Dis. 2021 Dec 1;21(1).

120. Mai LTP, Dung LP, Mai TNP, Hanh NTM, Than PD, Tran VD, et al. Characteristics of human leptospirosis in three different geographical and climatic zones of Vietnam: a hospital-based study. International Journal of Infectious Diseases. 2022 Jul 1;120:113–20.

121. Reis EAG, Hagan JE, Ribeiro GS, Teixeira-Carvalho A, Martins-Filho OA, Montgomery RR, et al. Cytokine Response Signatures in Disease Progression and Development of Severe Clinical Outcomes for Leptospirosis. PLoS Negl Trop Dis. 2013;7(9).

122. Ittyachen A, Lakshmanakumar VK, Eapen CK, Joseph MR. Methylprednisolone as adjuvant in treatment of acute respiratory distress syndrome owing to leptospirosis - a pilot study. Indian Journal of Critical Care Medicine [Internet]. 2005;9(3):133–6. Available from: www.ijccm.org

123. Galloway RL, Levett PN, Tumeh JW, Flowers CR. Assessing cost effectiveness of empirical and prophylactic therapy for managing leptospirosis outbreaks. Epidemiol Infect. 2009;137(9):1323–32.

124. Brett-Major DM, Lipnick RJ. Antibiotic prophylaxis for leptospirosis. Cochrane Database of Systematic Reviews [Internet]. 2009 Jul 8;2022(2). Available from: https://doi.wiley.com/10.1002/14651858.CD007342.pub2

125. Bernardo DCC, Blanquisco LR, Carpio GCA, Berba RP. OS126 Steroids for Pulmonary Involvement in Leptospirosis: A Meta-Analysis. Respirology [Internet]. 2013 Nov 18;18(S4):1–2. Available from: https://onlinelibrary.wiley.com/doi/10.1111/resp.12183

126. Charan J, Saxena D, Mulla S, Yadav P. Antibiotics for the Treatment of Leptospirosis: Systematic Review and Meta-Analysis of Controlled Trials. Int J Prev Med [Internet]. 2013 May 26;4(5):501–10. Available from: http://www.ncbi.nlm.nih.gov/pmc/articles/PMC3733179/

127. Cleto S, Malaque C, Rodrigues C, Sztajnbok J, Seguro A, Andrade L. Impact of different methods of renal replacement therapy in weil syndrome. Crit Care Med [Internet]. 2015 Dec;43(12):246. Available from: http://journals.lww.com/00003246-201512001-00981

128. Taylor AJ, Paris DH, Newton PN. A systematic review of the mortality from untreated leptospirosis. PLoS Negl Trop Dis [Internet]. 2015;9(6):e0003866-. Available from: http://www.plosntds.org/index.php

129. Cleto SA, Rodrigues CE, Malaque CM, Sztajnbok J, Seguro AC, Andrade L. Hemodiafiltration decreases serum levels of inflammatory mediators in severe leptospirosis: A prospective study. PLoS One. 2016 Aug 1;11(8).

130. Naing C, Reid SA, Aung K. Comparing antibiotic treatment for leptospirosis using network meta-analysis: A tutorial. BMC Infect Dis [Internet]. 2017;17(1):29. Available from: http://www.biomedcentral.com/bmcinfectdis/

131. Schneider M, Velasco-Hernandez J, Min K duk, Leonel D, Baca-Carrasco D, Gompper M, et al. The Use of Chemoprophylaxis after Floods to Reduce the Occurrence and Impact of Leptospirosis Outbreaks. Int J Environ Res Public Health [Internet]. 2017 Jun 3;14(6):594. Available from: http://www.mdpi.com/1660-4601/14/6/594

132. Lindow JC, Tsay AJ, Montgomery RR, Reis EAG, Wunder EA, Araújo G, et al. Elevated activation of neutrophil Toll-like receptors in patients with acute severe leptospirosis: An observational study. American Journal of Tropical Medicine and Hygiene. 2019;101(3):585–9.

133. Herath N, Uluwattage W, Weliwitiya T, Karunanayake L, Lekamwasam S, Ratnatunga N, et al. Sequel and therapeutic modalities of leptospirosis associated severe pulmonary haemorrhagic syndrome (SPHS); A Sri Lankan experience. BMC Infect Dis. 2019 May 22;19(1).

134. Gasem MH, Hadi U, Alisjahbana B, Tjitra E, Hapsari MMDEAH, Lestari ES, et al. Leptospirosis in Indonesia: Diagnostic challenges associated with atypical clinical manifestations and limited laboratory capacity. BMC Infect Dis. 2020 Feb 27;20(1).

135. Guzmán Pérez M, Blanch Sancho JJ, Segura Luque JC, Mateos Rodriguez F, Martínez Alfaro E, Solís García Del Pozo J. Current evidence on the antimicrobial treatment and chemoprophylaxis of human leptospirosis: A meta-analysis. Pathogens. 2021 Sep 1;10(9).

136. Beri D, Moola S, Jagnoor J, Salam A, Bhaumik S. Prevention, control and management of leptospirosis in India: An evidence gap map. Trans R Soc Trop Med Hyg [Internet]. 2021;115(12):1353–61. Available from: http://trstmh.oxfordjournals.org/content/by/year/2013

137. Siatan JE, Burog AIL, Chavez J, Danguilan R, Catli-Burog CA. Incidence of clinically significant bleeding and thromboembolism among patients with leptospirosis requiring extra corporeal membrane oxygenation: A descriptive study. Perfusion. 2022;37(1 SUPPL):38–9.

138. Gupta N, Wilson W, Ravindra P, Raghu R, Saravu K. Coinfection of leptospirosis and coronavirus disease 2019: A retrospective case series from a coastal region in South India. J Med Virol. 2022 Sep 1;94(9):4508–11.

139. Smith S, Liu YH, Carter A, Kennedy BJ, Dermedgoglou A, Poulgrain SS, et al. Severe leptospirosis in tropical Australia: Optimising intensive care unit management to reduce mortality. PLoS Negl Trop Dis. 2019 Dec 1;13(12).

140. Selvarajah S, Ran S, Roberts NW, Nair M. Leptospirosis in pregnancy: A systematic review. PLoS Negl Trop Dis. 2021 Sep 1;15(9).

141. Rashuaman-Conche BH, Loli-Guevara S, Rodriguez-Lopez E, Alva-Diaz C. Efficacy and safety of pre-exposure of antibiotic prophylaxis for leptospirosis: Protocol for a systematic review and meta-analysis. medRxiv [Internet]. 2021;((Rashuaman-Conche, Loli-Guevara, Rodriguez-Lopez) Sociedad Cientifica de San Fernando, Lima, Peru(Rashuaman-Conche, Loli-Guevara, Rodriguez-Lopez) Facultad de Medicina Humana San Fernando, Universidad Nacional Mayor de San Marcos, Lima, Peru(Rodriguez-Lop). Available from: https://www.medrxiv.org/

142. Shrestha M, Choudhury SS, Carolin S V, Rani A, Roy I, Zahir F, et al. Leptospirosis in Pregnancy: Prevalence, Risk Factors, Clinical Characteristics, and Outcomes in a North Indian Population. MedRxiv preprint [Internet]. 2022; Available from: https://doi.org/10.1101/2022.11.02.22281830

143. Murali K V, Sujay R, Pavithran S, Thomas M. Intracranial bleeding in Weil’s disease. J Postgrad Med [Internet]. 2002;48(2):158. Available from: http://www.ncbi.nlm.nih.gov/pubmed/12215709

144. Chawla V, Trivedi TH, Yeolekar ME. Epidemic of leptospirosis: an ICU experience. J Assoc Physicians India [Internet]. 2004 Aug;52:619–22. Available from: http://www.ncbi.nlm.nih.gov/pubmed/15847354

145. O’Leary FM, Hunjan JS, Bradbury R, Thanakrishnan G. Fatal leptospirosis presenting as musculoskeletal chest pain. Medical Journal of Australia. 2004 Jan 1;180(1):29–31.

146. De Francesco Daher E, Zanetta DMT, Abdulkader RCRM. Pattern of renal function recovery after leptospirosis acute renal failure. Nephron Clin Pract. 2004;98(1).

147. Markum HM. Renal involvement in leptospirosis at Dr. Cipto Mangunkusumo and Persahabatan Hospitals. Acta Med Indones [Internet]. 2004;36(3):148–52. Available from: http://www.ncbi.nlm.nih.gov/pubmed/15557684

148. Cetin BD, Harmankaya O, Hasman H, Gunduz A, Oktar M, Seber E. Acute renal failure: A common manifestation of leptospirosis. Ren Fail. 2004;26(6):655–61.

149. Abuauad MC, Osorio G, Rojas JL, Pino L. [Leptospirosis: report of a fulminant fatal case and review]. Revista chilena de infectologia [Internet]. 2005 Mar;22(1):93–7. Available from: http://www.ncbi.nlm.nih.gov/pubmed/15798875

150. Spichler AS, Vilaça PJ, Athanazio D a., Albuquerque JOM, Buzzar M, Castro B, et al. Predictors of lethality in severe leptospirosis in urban Brazil. American Journal of Tropical Medicine and Hygiene. 2008;79(6):911–4.

151. Kang SJ, Ju Lee K, Park KH, Jung SI. Clinical features of leptospirosis experienced in a university hospital between 2001 and 2007. Korean Journal of Medicine. 2009;77(4):453–60.

152. Neligan A, Mullins GM, O’Sullivan SS, Fitzgerald P, Harrington H. A rare cause of fatal intracranial haemorrhage. Ir J Med Sci. 2009 Sep;178(3):343–5.

153. Sakellaridis N, Panagopoulos D, Androulis A. Neuroleptospirosis with hydrocephalus and very elevated cerebrospinal fluid protein. South Med J [Internet]. 2009 May;102(5):549–50. Available from: http://www.ncbi.nlm.nih.gov/pubmed/19373140

154. Gerasymchuk L, Swami A, Carpenter CF, Samarapungavan D, Batke M, Kanhere R, et al. Case of fulminant leptospirosis in a renal transplant patient. Transplant Infectious Disease. 2009 Oct;11(5):454–7.

155. Velasco O, Velasco-Castrejón O, Rivas-Sánchez B, Soriano-Rosas J, Hugo Rivera-Reyes H. Daño miocárdico grave por leptospirosis. Informe de un caso fatal en México. Arch Cardiol Mex [Internet]. 2009;79(4):268–73. Available from: www.elsevier.com.mx

156. Niwattayakul K, Kaewtasi S, Chueasuwanchai S, Hoontrakul S, Chareonwat S, Suttinont C, et al. An open randomized controlled trial of desmopressin and pulse dexamethasone as adjunct therapy in patients with pulmonary involvement associated with severe leptospirosis. Clinical Microbiology and Infection. 2010;16(8):1207–12.

157. Inoue T, Yoshikawa K, Tada M, Nakamura T, Hinoshita F. Two cases of Weil’s disease with acute renal failure in the central Tokyo metropolitan area. Clin Nephrol. 2010;73(1):76–80.

158. Trivedi S V, Vasava AH, Bhatia LC, Patel TC, Patel NK, Patel NT. Plasma exchange with immunosuppression in pulmonary alveolar haemorrhage due to leptospirosis. Indian J Med Res [Internet]. 2010 Mar;131:429–33. Available from: http://www.ncbi.nlm.nih.gov/pubmed/20418558

159. Walter B, Wein B, Bittinger M, Messmann H. Leptospirose (Morbus Weil) in Augsburg: Drei Fälle und ein Überblick. Deutsche Medizinische Wochenschrift. 2010;135(14):675–8.

160. Zaki SA, Shanbag P. Clinical manifestations of dengue and leptospirosis in children in Mumbai: An observational study. Infection. 2010 Aug;38(4):285–91.

161. Kularatne SAM, Budagoda BDSS, de Alwis VKD, Wickramasinghe WMRS, Bandara JMRP, Pathirage LPMMK, et al. High efficacy of bolus methylprednisolone in severe leptospirosis: a descriptive study in Sri Lanka. Postgrad Med J [Internet]. 2011 Jan 1;87(1023):13–7. Available from: NS  - Kularatne 2011

162. Echeverri-Toro LM, Penagos S, Castañeda L, Villa P, Atehortúa S, Ramírez F, et al. Características sociodemográficas y clínicas de pacientes con infección por Leptospira spp. atendidos en cuatro centros hospitalarios de Medellín, Colombia, 2008-2013. Biomedica. 2017;37(1):62–7.

163. Alventosa Mateu C, Plana Campos L, Larrey Ruíz L, Acedo Mayordomo R, Sanchís Artero L, Peño Muñoz L, et al. [Gastrointestinal bleeding and acute hepatic failure by leptospirosis: an entity that should not be forgotten]. Revista de gastroenterologia del Peru [Internet]. 2017;37(1):96–9. Available from: http://www.ncbi.nlm.nih.gov/pubmed/28489846

164. Neaterour P, Rivera A, Galloway RL, Negrón MG, Rivera-Garcia B, Sharp TM. Case report: fatal leptospira spp./Zika virus coinfection - Puerto Rico, 2016. American Journal of Tropical Medicine and Hygiene. 2017;97(4):1085–7.

165. Lin PC, Chi CY, Ho MW, Chen CM, Ho CM, Wang JH. Demographic and clinical features of leptospirosis: three-year experience in central Taiwan. Journal of microbiology, immunology, and infection. 2008 Apr;41(2):145–50.

166. Camous L, Pommier JD, Tressières B, Martino F, Picardeau M, Loraux C, et al. Organ Involvement Related to Death in Critically Ill Patients with Leptospirosis: Unsupervised Analysis in a French West Indies ICU. Crit Care Explor. 2024 Jul 8;6(7):e1126.

167. SLCTR/2018/016. ‘Effectiveness and safety of the use of N-acetylcysteine in patients with kidney failure in leptospirosis’. 2018; Available from: https://www.cochranelibrary.com/central/doi/10.1002/central/CN-01907317/full

168. Lebedev V V, Zhuravlev AI, Zotov S V, Lebedev P V, Pronin MG, Podsadniaia AA, et al. [Use of infusion solution remaxol in the combination treatment of patients with leptospirosis]. Ter Arkh [Internet]. 2013;85(11):58–61. Available from: http://www.ncbi.nlm.nih.gov/pubmed/24432601

169. Gangula RS, Prabhu MM, Stanley W. Weil syndrome causing autoimmune haemolytic anaemia. Natl Med J India [Internet]. 2019;32(2):88–9. Available from: http://www.ncbi.nlm.nih.gov/pubmed/31939404

170. Puca E, Abazaj E, Pipero P, Harxhi A, Ferizaj R, Como N, et al. A case with high bilirubinemia and hemolytic anemia during leptospirosis and a short review of similar cases. Caspian J Intern Med. 2020;11(4):441–5.

171. Drazilova S, Martinkova D, Vojtek A. Mysterious Jaundice. Eur J Case Rep Intern Med. 2019 Oct 28;(Vol 6 Sup 1):427.

172. Cantwell T, Ferre A, Van Sint Jan N, Blamey R, Dreyse J, Baeza C, et al. Leptospirosis-associated catastrophic respiratory failure supported by extracorporeal membrane oxygenation. Journal of Artificial Organs. 2017 Dec 1;20(4):371–6.

173. Jean-Marie J, Calmont I, Djossou F, Curlier E, Cabie A, Niang M, et al. Comparing Two Antibiotic Therapy Periods (3 Versus 7 Days) in Patients With Mild Leptospirosis and Seen at the Hospital in 5 French Overseas Departments (Martinique, Guadeloupe, French Guiana, Reunion, Mayotte) (LEPTO3) [Internet]. 2019 [cited 2023 Nov 23]. Available from: https://classic.clinicaltrials.gov/show/NCT04211649

174. Aydemir H, Akduman D, Oztoprak N, Pişkin N, Celebi G, Akkoyunlu Y. A severe case of Weil’s disease. Mikrobiyol Bul [Internet]. 2007 Jan;41(1):145–50. Available from: http://www.ncbi.nlm.nih.gov/pubmed/17427565

175. Céspedes M, Tapia R, Balda L, Gonzalez D, Glenny M, Vinetz JM. Brote de leptospirosis asociado a la natacion en una fuente de agua subterranea en una zona costera, Lima - Peru. Rev Peru Med Exp Salud Publica. 2009;26(4):441–89.

176. Tatopoulos A, Herbain D, Kazmierczak C, Bollaert PE, Gibot S. Parenteral use of recombinant  activated factor VII during  diffuse alveolar hemorrhage  secondary to leptospirosis. Intensive Care Med [Internet]. 2010;36(3):555–6. Available from: https://dx.doi.org/10.1007/s00134-009-1718-7

177. Asaduzzaman M, Karmaker L, Rahman A, Rahman MS, Awaul MA, Chakraborty SR. Dengue and leptospirosis coinfection: a case series. J Med Case Rep. 2024 Dec 1;18(1).

178. Watt G, Linda Tuazon M, Santiago E, Padre LP, Calubaquib C, Ranoa CP, et al. Placebo-Controlled Trial of Intravenous Penicillin for Severe and Late Leptospirosis. The Lancet. 1988;331(8583):433–5.

179. Ranawaka N, Jeevagan V, Karunanayake P, Jayasinghe S. Pancreatitis and myocarditis followed by pulmonary hemorrhage, a rare presentation of leptospirosis- A case report and literature survey. BMC Infect Dis. 2013 Jan 24;13(1).

180. Sivaprakasam V, Zochowski WJ, Palmer MF. Clinical spectrum of severe leptospirosis in the UK. JMM Case Rep. 2014 Dec 1;1(4).

181. Kumar A, Majumdar B, Goru B, Tewari R, Kumar D, Pandey A. Chorzy trudni nietypowi/Case report A case of complete heart block in a patient with HIV and leptospirosis Całkowity blok przedsionkowo-komorowy u chorego z HIV i leptospirozą. Kardiol Pol [Internet]. 2010;68:562–3. Available from: www.kardiologiapolska.pl

182. Panagopoulos P, Ganitis A, Papanas N, Iosifidou G, Maltsan T, Kioutsouk S, et al. Leptospirosis: a report on a series of five autochthonous cases in a Greek region. Journal of Chemotherapy. 2016 Sep 2;28(5):428–31.

183. Škerk V, Markotić A, Puljiz I, Kuzman I, Tošev EČ, Habuš J, et al. Electrocardiographic changes in hospitalized patients with leptospirosis over a 10-year period. Medical Science Monitor. 2011;17(7).

184. Campbell S, Stewart J, Quail G, Withey G, Smith S, Hanson J. Case Report: Leptospirosis Complicated by Persistent, Bilateral Sensorineural Hearing Loss. Am J Trop Med Hyg. 2023 Dec 6;109(6):1238–41.

185. McClain JB, Ballou WR, Harrison SM, Steinweg DL. Doxycycline therapy for leptospirosis. Ann Intern Med. 1984;100(5):696–8.

186. Miura K, Kakimoto T, Kawada M, Oshima K, Miyanaga R, Shinozaki H, et al. [Case report severe leptospirosis with ARDS in Japan]. Nihon Naika Gakkai Zasshi [Internet]. 2013;102(12):3215–8. Available from: https://search.bvsalud.org/portal/resource/en/mdl-24605571

187. Cagliero J. Study of the Incidence of Jarisch-Herxheimer Reaction in Leptospirosis Patients in New Caledonia (LEPJARNC) [Internet]. 2021 [cited 2024 May 26]. Available from: https://classic.clinicaltrials.gov/ct2/show/NCT04882046

188. Tse KC, Yip PS, Hui KM, Li FK, Yuen KY, Lai KN, et al. Potential benefit of plasma exchange in treatment of severe icteric leptospirosis complicated by acute renal failure. Clin Diagn Lab Immunol. 2002;9(2):482–4.

189. Arcilla MS, Wismans PJ, van Beek-Nieuwland Y, van Genderen PJ. Severe leptospirosis in a Dutch traveller returning from the Dominican Republic, October 2011. Eurosurveillance. 2012 Mar 29;17(13).

190. Gallardo C, Asner S, Manuel O, Berthod D, Williams-Smith J, Jaton K, et al. Leptospirose familiale après rafting en Thaïlande. Rev Med Suisse [Internet]. 2015;11(470):872–6. Available from: https://www.revmed.ch/view/content/302978/full/1/302885/536595

191. Schenk LK, Pavenstädt H, Kümpers P. Fever and Pulmo-Renal Syndrome with Cholecystitis in a Traveller - A Differential Diagnostic Challenge. Deutsche Medizinische Wochenschrift. 2018 Feb 1;143(4):263–6.

192. Silva Cevallos D, Aragón Quijano L, Silva Guachilema D, Silva Michalón F. Leptospirosis y enfermedad de Weil. Reporte de caso clínico. Revista Medicina e Investigación Clínica Guayaquil. 2022 May 15;3(4):51–5.

193. Cocchi S, Codeluppi M, Guaraldi G, Squillace N, Bedini A, Venturelli C, et al. Invasive pulmonary and cerebral aspergillosis in a patient with Weil’s disease. Scand J Infect Dis [Internet]. 2005;37(5):396–8. Available from: http://www.ncbi.nlm.nih.gov/pubmed/16051585

194. Meaudre É, Asencio Y, Montcriol A, Martinaud C, Graffin B, Palmier B, et al. Immunomodulation au cours d’un cas de leptospirose grave avec défaillance multiviscérale : échanges plasmatiques, immunoglobulines ou corticoïdes ? Ann Fr Anesth Reanim. 2008 Feb;27(2):172–6.

195. Wennike N, Bacon M. The dangers of volunteer work in rural Asia - Acute lung injury as the first manifestation of Weil’s disease. Eur J Intern Med. 2008 Dec;19(8).

196. Chauhan V, Mahesh DM, Panda P, Mokta J, Thakur S. Leptospirosis presenting as acute respiratory distress syndrome (ARDS) in sub-Himalayan region. J Assoc Physicians India [Internet]. 2010 Jun;58:390–1. Available from: http://www.ncbi.nlm.nih.gov/pubmed/21125784

197. Dabbas L, V BM. Unique Presentation of Leptospirosis in an Urban Setting: The Interplay of Social Determinants and Clinical Diagnostics. Am J Respir Crit Care Med [Internet]. 2024;209((Dabbas, Baez Martinez) Internal Medicine, Medstar Washington Hospital Center, Washington, DC, United States). Available from: https://www.atsjournals.org/doi/abs/10.1164/ajrccm-conference.2024.209.1_MeetingAbstracts.A3590https://discover.lshtm.ac.uk/openurl/44HYG/44HYG_services_page?sid=OVID:embase&id=doi:10.1164%2Fajrccm-conference.2024.209.1_meetingabstracts.a3590&id=pmid&issn

198. Zamri Z, Shaker AH, Razman J. Leptospirosis complicating with acute large bowel gangrene: a case report. Clin Ter [Internet]. 2012;163(5):399–400. Available from: http://www.ncbi.nlm.nih.gov/pubmed/23099969

199. Lau CL, DePasquale JM. Leptospirosis Diagnostic Challenges, American Samoa. Emerg Infect Dis. 2012 Dec;18(12):2079–81.

200. Ghouse M, Maulana AB, Mohamed Ali MG, Sarasa VD. A two-year study of the efficacy of azithromycin in the treatment of leptospirosis in humans. Indian J Med Microbiol [Internet]. 2006 Oct;24(4):345–6. Available from: http://www.ncbi.nlm.nih.gov/pubmed/17185874

201. Kshirsagar PP, Sonavane AD, Doshi AC, Teltumbde U, Mandalik S. Atypical presentation of leptospirosis. J Assoc Physicians India [Internet]. 2010 Feb;58:117–8. Available from: http://www.ncbi.nlm.nih.gov/pubmed/20653156

202. O’Toole SM, Pathak N, Toms GC, Gelding S V, Sivaprakasam V. Fever, jaundice and acute renal failure. Clin Med (Lond) [Internet]. 2015;15(1):58–60. Available from: https://www.ncbi.nlm.nih.gov/pmc/articles/PMC4954526https://dx.doi.org/10.7861/clinmedicine.15-1-58

203. Sathiyakumar V, Shah NP, Niranjan-Azadi A, Tao J, Tsao A, Martin IW, et al. Snowflakes in August: Leptospirosis Hemorrhagic Pneumonitis. American Journal of Medicine. 2017 Jan 1;130(1):e9–11.

204. Kunikowska AJ, Wildgruber M, Schulte-Frohlinde E, Lahmer T, Schmid RM, Huber W. Liver function assessment using indocyanine green plasma disappearance rate in a young male with icteric leptospirosis: A case report. BMC Infect Dis. 2019 May 28;19(1).

205. Moreira Marques T, Nascimento PO, Almeida A, Tosatto V. Weil’s disease in a young homeless man living in Lisbon. BMJ Case Rep [Internet]. 2020 Jun 11;13(6):e233543. Available from: https://casereports.bmj.com/lookup/doi/10.1136/bcr-2019-233543

206. Schattner A, Dubin I, Glick Y, Nissim E. Acute painless pancreatitis as an unusual presentation of leptospirosis in a low-incidence country. BMJ Case Rep. 2020 Aug 24;13(8).

207. Weiner M, Coen M, Serratrice J, Mavrakanas TA, Leidi A. Acute kidney injury with partial Fanconi syndrome in a patient with leptospirosis: a case report. J Med Case Rep. 2021 Dec 1;15(1).

208. Fairburn AC, Semple SJG. Chloramphenicol and Penicillin in the treatment of Leptospirosis among British troops in Malaya. The Lancet. 1956;267(6906):13–6.

209. Song ATW, Abas L, Andrade LC, Andraus W, D’Albuquerque LAC, Abdala E. A first report of leptospirosis after liver transplantation. Transplant Infectious Disease. 2016 Feb 1;18(1):137–40.

210. Wang N, Han YH, Sung JY, Lee W Sen, Ou TY. Atypical leptospirosis: An overlooked cause of aseptic meningitis. BMC Res Notes. 2016 Mar 10;9(1).

211. RUSSELL RW. Treatment of leptospirosis with oxytetracycline. Lancet. 1958;2(7057):1143–5.

212. Phimda K, Hoontrakul S, Suttinont C, Chareonwat S, Losuwanaluk K, Chueasuwanchai S, et al. Doxycycline versus azithromycin for treatment of leptospirosis and scrub typhus. Antimicrob Agents Chemother. 2007;51(9):3259–63.

213. Behera B, Chaudhry R, Pandey A, Mohan A, Dar L, Premlatha MM, et al. Co-infections due to leptospira, dengue and hepatitis E: a diagnostic challenge. J Infect Dev Ctries [Internet]. 2009 Nov 5;4(1):48–50. Available from: http://www.ncbi.nlm.nih.gov/pubmed/20130379

214. Céspedes Pereña V, Silveira YP, Soria LC, Boloy MD, Wilson EN. Intervención de enfermería en pacientes con leptospirosis en Unidad de Cuidados Intensivos. Revista Información Científica [Internet]. 2014 [cited 2025 Sep 20];4(86):643–51. Available from: https://revinfcientifica.sld.cu/index.php/ric/article/view/1047

215. Maier A, Kaeser R, Thimme R, Boettler T. Acute pancreatitis and vasoplegic shock associated with leptospirosis - A case report and review of the literature. BMC Infect Dis. 2019 May 8;19(1).

216. Liu YH, Chen YH, Chen CM. Fulminant Leptospirosis Presenting with Rapidly Developing Acute Renal Failure and Multiorgan Failure. Biomedicines. 2024 Feb 1;12(2).

217. Friedland JS, Warrell DA. The Jarisch-Herxheimer Reaction in Leptospirosis: Possible Pathogenesis and Review. Clinical Infectious Diseases [Internet]. 1991 Mar 1;13(2):207–10. Available from: http://academic.oup.com/cid/article/13/2/207/521404/The-JarischHerxheimer-Reaction-in-Leptospirosis

218. Pappas G, Akritidis N, Christou L, Mastora M, Tsianos E. Unusual causes of reactive arthritis: Leptospira and Coxiella burnetii. Clin Rheumatol. 2003 Oct;22(4–5):343–6.

219. Seilmaier M, Guggemos W. Schwerer fieberhafter infekt mit nierenbeteiligung nach Südostasienreise. Internist. 2008 Nov;49(11):1372–8.

220. Efe C, Nasirğlu N, Ozaslan E, Yuksel O, Tunca H, Sennaroglu E. Ascites and peritonitis due to leptospirosis. Eur J Gastroenterol Hepatol [Internet]. 2010 Mar;22(3):376–7. Available from: https://journals.lww.com/00042737-201003000-00019

221. Peter G, Narasimha H. Acalculous cholecystitis: A rare presentation of leptospirosis progressing to Weil’s disease. Asian Pac J Trop Med [Internet]. 2011 Dec;4(12):1007–8. Available from: http://linkinghub.elsevier.com/retrieve/pii/S1995764511602356

222. Lagi F, Corti G, Meli M, Pinto A, Bartoloni A. Leptospirosis acquired by tourists in Venice, Italy. J Travel Med. 2013 Mar;20(2):128–30.

223. Herath NJ, Kularatne SA, Weerakoon KG, Wazil A, Subasinghe N, Ratnatunga N V. Long term outcome of acute kidney injury due to leptospirosis? A longitudinal study in Sri Lanka. BMC Res Notes. 2014 Jun 25;7(1).

224. Ríos DI, Chaparro-Solano HM. Challenge in clinical diagnosis and treatment of leptospirosis. Revista Ciencias de la Salud. 2015;13(1):91–7.

225. Karrasch M, Herfurth K, Kläver M, Miethke J, Mayer-Scholl A, Luge E, et al. Severe leptospirosis complicated by Epstein–Barr Virus reactivation. Infection. 2015 Dec 1;43(6):763–9.

226. Taylor D, Karamadoukis L. Plasma exchange in severe leptospirosis with multi-organ failure: A case report. J Med Case Rep. 2013;7.

227. Hall MT, Do TA, Shusko MP. The value of pre-exposure prophylaxis: A case series of US Marines infected with leptospirosis. Travel Med Infect Dis. 2023 Mar 1;52.

228. Chiu HH, Juan HL, Lu PL, Chang K. A graveyard keeper with severe hyperbilirubinemia caused by leptospirosis. Journal of Microbiology, Immunology and Infection. 2021 Jun 1;54(3):536–7.

229. Centre Hospitalier Universitaire de la Réunion. NCT05413720 - Study of the Innate Immune Response to the Acute Phase of Human Leptospirosis-IMMUNOLEPTO [Internet]. 2022 [cited 2024 May 26]. Available from: https://classic.clinicaltrials.gov/ct2/show/NCT05413720

230. Dahanayaka NJ, Agampodi SB, Seneviratna I, Warnasekara J, Rajapakse R, Ranathunga K, et al. Clinical spectrum of endemic leptospirosis in relation to cytokine response. PLoS One. 2021 Dec 1;16(12 December).

231. Zhang XC, Lei XQ, Sun Y, Shan NB. Leptospirosis manifested with severe pulmonary hemorrhagic syndrome successfully treated with veno-venous extracorporeal membrane oxygenation A case report and literature review. Medicine (United States). 2024 Dec 20;103(51):e40942.

232. Suputtamongkol Y, Pongtavornpinyo W, Lubell Y, Suttinont C, Hoontrakul S, Phimda K, et al. Strategies for diagnosis and treatment of suspected leptospirosis: a cost-benefit analysis. PLoS Negl Trop Dis [Internet]. 2010 Jan [cited 2014 Dec 13];4(2):e610. Available from: http://www.pubmedcentral.nih.gov/articlerender.fcgi?artid=2826401&tool=pmcentrez&rendertype=abstract

233. Sehgal SC, Sugunan AP, Murhekar M V, Sharma S, Vijayachari P. Randomized controlled trial of doxycycline prophylaxis against leptospirosis in an endemic area. Int J Antimicrob Agents [Internet]. 2000;13:249–55. Available from: www.ischemo.org

234. Osthoff M. NCT05300425 - Seroprevalence of Leptospirosis in Val Müstair, Switzerland [Internet]. 2022 [cited 2024 May 26]. Available from: https://classic.clinicaltrials.gov/ct2/show/NCT05300425

235. Takafuji ET, Kirkpatrick JW, Miller RN, Karwacki JJ, Kelley PW, Gray MR, et al. An Efficacy Trial of Doxycycline Chemoprophylaxis against Leptospirosis. New England Journal of Medicine [Internet]. 1984 Feb 23;310(8):497–500. Available from: http://www.nejm.org/doi/abs/10.1056/NEJM198402233100805

236. Sandoval KL, Cada KJS, Dimasin RVD, Labana R V. A One Health approach to the prevention, control, and management of leptospirosis: a scoping review. Discover public health [Internet]. 2025;22(1):108. Available from: https://link.springer.com/journal/12982https://discover.lshtm.ac.uk/openurl/44HYG/44HYG_services_page?sid=OVID:embase&id=doi:10.1186%2Fs12982-025-00489-7&id=pmid&issn=3005-0774&isbn=&volume=22&issue=1&spage=108&pages=&date=2025&title=Discover+public+healt

237. Beder M. NCT03497572 - Study to Evaluate the Persistence of Anti-leptospira Antibody in Subjects Vaccinated With Spirolept® Vaccine (SPT-001) [Internet]. 2018 [cited 2024 May 26]. Available from: https://classic.clinicaltrials.gov/ct2/show/NCT03497572

238. Gonsalez CR, Casseb J, Monteiro FG, Paula-Neto JB, Fernandez RB, Silva M V, et al. Use of doxycycline for leptospirosis after high-risk exposure in São Paulo, Brazil. Rev Inst Med Trop Sao Paulo [Internet]. 1998 Jan;40(1):59–61. Available from: http://www.ncbi.nlm.nih.gov/pubmed/9713140

239. Illangasekera VLU, Kularatne SAM, Kumarasiri PVR, Pussepitiya D, Premaratne MDN. Is oral penicillin an effective chemoprophylaxis against leptospirosis? A placebo controlled field study in the Kandy District, Sri Lanka. Southeast Asian J Trop Med Public Health [Internet]. 2008 Sep;39(5):882–4. Available from: http://www.ncbi.nlm.nih.gov/pubmed/19058584

240. Shivaraj B, Ts R, Anithraj BY, Bayari R. A study on prophylactic doxycycline to reduce the incidence of leptospirosis among paddy field farmers in a coastal district of India. International Journal of Infectious Diseases [Internet]. 2012 Jun;16:e462. Available from: https://linkinghub.elsevier.com/retrieve/pii/S1201971212008193

241. Guidugli F, Castro AA, Atallah ÁN. Antibiotics for preventing leptospirosis. In: Guidugli F, editor. The Cochrane Database of Systematic Reviews [Internet]. Chichester, UK: John Wiley & Sons, Ltd; 2000. Available from: https://doi.wiley.com/10.1002/14651858.CD001305

242. Alikhani A, Salehifar E, Zameni F, Rafiei A, Yazdani-Charati J, Delavaryan L, et al. Comparison of azithromycin vs doxycycline prophylaxis in leptospirosis, a randomized double blind placebo-controlled trial. J Infect Dev Ctries. 2018;12(11):991–5.

243. Gómez-Martín MC, Rodríguez-Benjumeda LM, de Eguilior-Mestre MC, Lozano-Domínguez MC, Luque-Márquez R, Jódar-Sánchez F, et al. Epidemiología de la leptospirosis en los humedales del sur de España. Gac Sanit [Internet]. 2023 Jan 1;37. Available from: https://linkinghub.elsevier.com/retrieve/pii/S021391112300002X

244. Gilks CF, Lambert HP, Broughton ES, Baker CC. Failure of penicillin prophylaxis in laboratory acquired leptospirosis. Postgrad Med J [Internet]. 1988 Mar 1;64(749):236–8. Available from: https://academic.oup.com/pmj/article/64/749/236/7047609

245. Turhan V, Atasoyu EM, Solmazgul E, Evrenkaya R, Cavuslu S. Anicteric leptospirosis and renal involvement. Ren Fail. 2005;27(4):491–2.

246. Jean-Marie J. Study of Decreasing Kinetics of the Leptospiremia During Antibiotic Treatment of Leptospirosis in Martinique (Ciné LEPTO; NCT02000635) [Internet]. 2022 Mar [cited 2023 Nov 23]. Available from: https://classic.clinicaltrials.gov/show/NCT02000635

247. Juhel C. NCT02898519 - Immune Response After Leptospirosis Infection (SERO7LEPTO) [Internet]. 2016 [cited 2024 May 26]. Available from: https://classic.clinicaltrials.gov/ct2/show/NCT02898519

248. Francois C, Diallo K, Fayeulle S, Raffray L. Chronic Post-leptospirosis Manifestations in Reunion (LEPTONIC) [Internet]. 2022 [cited 2023 Nov 23]. Available from: https://classic.clinicaltrials.gov/show/NCT05436756

249. Binder WD, Mermel LA. Leptospirosis in an urban setting: case report and review of an emerging infectious disease. J Emerg Med [Internet]. 1998 Nov;16(6):851–6. Available from: https://linkinghub.elsevier.com/retrieve/pii/S0736467998000973

250. Assez N, Mauriaucourt P, Cuny J, Goldstein P, Wiel E. Ictère fébrile... et si c’était une leptospirose. À propos d’un cas de L.interrogans Icterohaemorrhagiae dans le Nord de la France. Ann Fr Anesth Reanim. 2013 Jun;32(6):439–43.

251. Bandara AGNMK, Kalaivarny G, Perera N, Indrakumar J. Aseptic meningitis as the initial presentation of Leptospira borgpetersenii serovar Tarassovi: two case reports and a literature review. BMC Infect Dis. 2021 Dec 1;21(1).

252. Lakmali JPR, Thirumavalavan K, Dissanayake D. A rare case of posterior reversible encephalopathy syndrome in a patient with severe leptospirosis complicated with rhabdomyolysis and acute kidney injury; a case report. BMC Infect Dis. 2021 Dec 1;21(1).

253. Maldonado F, Portier H, Kisterman JP. Bilateral facial palsy in a case of leptospirosis. Scand J Infect Dis. 2004;36(5):386–7.

254. Makhija P, Gopinath S, Kannoth S, Radhakrishnan K. A case of post-leptospirosis autoimmune epilepsy presenting with sleep-related hypermotor seizures. Epileptic Disorders. 2017 Dec 1;19(4):456–60.

255. Shiddapur G, Adapa SM, Vutukuru KKR, Kondapalli MP, Agarwal S. A Case of Acute Disseminated Encephalomyelitis in Adults: Unravelling the Influenza B and Leptospirosis Co-infection. Journal of Clinical and Diagnostic Research [Internet]. 2024;18(6):OD07EP-OD10. Available from: https://www.jcdr.net/article_fulltext.asp?issn=0973-709x&year=2024&month=June&volume=18&issue=6&page=OD07-OD10&id=19497https://discover.lshtm.ac.uk/openurl/44HYG/44HYG_services_page?sid=OVID:embase&id=doi:10.7860%2FJCDR%2F2024%2F70148.19497&id=pmid&issn=2

256. Siddiqui A, Randev S, Singla N, Dhavan A, Guglani V, Gupta V. Acute disseminated encephalomyelitis due to a zoonotic co-infection. J Trop Pediatr. 2023 Feb 1;69(1).

257. Finsterer J, Stöllberger C, Sehnal E, Stanek G. Mild leptospirosis with three-year persistence of IgG- and IgM-antibodies, initially manifesting as carpal tunnel syndrome. Journal of Infection. 2005 Aug;51(2).

258. George P. Two uncommon manifestations of leptospirosis: Sweet’s syndrome and central nervous system vasculitis. Asian Pac J Trop Med [Internet]. 2011 Jan;4(1):83–4. Available from: http://linkinghub.elsevier.com/retrieve/pii/S1995764511600412

259. Baburaj P, Varma SS, Harikrishnan BL. Hypokalemic paralysis in leptospirosis. J Assoc Physicians India [Internet]. 2012 Mar;60:53–4. Available from: http://www.ncbi.nlm.nih.gov/pubmed/22799118

260. Alvarez-Torres E, Acaba LA, Berrocal MH. Sub-internal limiting membrane hemorrhage associated with Weil’s disease. Am J Ophthalmol Case Rep. 2019 Dec 1;16.

261. Sengupta M, Latha T, Mandal S, Mukhopadhyay K. Foetal outcome of Leptospira and Rickettsial infections during pregnancy: a systematic review. Trans R Soc Trop Med Hyg. 2024 Dec 1;118(12):814–28.

262. Sundarsingh V, Kumar RM, Kulkarni M, Mammoo FR, Rodrigues PR, Prashanth YM. Unusual presentation of Sjogren’s syndrome during pregnancy: a case report. J Med Case Rep. 2024 Dec 1;18(1).

263. Shaked Y, Shpilberg O, Samra D, Samra Y. Leptospirosis in Pregnancy and Its Effect on the Fetus: Case Report and Review. Clinical Infectious Diseases [Internet]. 1993 Aug 1;17(2):241–3. Available from: https://academic.oup.com/cid/article-lookup/doi/10.1093/clinids/17.2.241

264. Carrillo-Larco RM, Altez-Fernandez C, Acevedo-Rodriguez JG, Ortiz-Acha K, Ugarte-Gil C. Leptospirosis as a risk factor for chronic kidney disease: A systematic review of observational studies. PLoS Negl Trop Dis. 2019 May 1;13(5).

265. Yap DYH, Chan GSW, Chan KW, Kwan LPY, Wong WTK, Lam MF, et al. Cortical necrosis in a kidney transplant recipient due to leptospirosis. Nephrology. 2014;19(4):257–8.

266. Nattachai S. NCT05425524 - Leptospirosis Care Bundle Study [Internet]. 2019 [cited 2024 May 26]. Available from: https://classic.clinicaltrials.gov/ct2/show/NCT05425524

267. Macaraeg CS, Aldecoa KA. FOREST MYSTERY TO CRITICAL CARE: A RARE CASE OF WEIL’S SYNDROME IN A NONENDEMIC AREA WITH EXTREME HYPERBILIRUBINEMIA AND COMPLICATED BY A LEUKEMOID REACTION. Chest [Internet]. 2024;166(4 Supplement):A2935EP – A2936. Available from: https://discover.lshtm.ac.uk/openurl/44HYG/44HYG_services_page?sid=OVID:embase&id=doi:10.1016%2Fj.chest.2024.06.1769&id=pmid&issn=0012-3692&isbn=&volume=166&issue=4+Supplement&spage=A2935&pages=A2935-A2936&date=2024&title=Chest&atitle=FOREST+MYSTERY+TO+CR

268. Shah I. Non-oliguric renal failure - A presentation of leptospirosis. Ann Trop Med Parasitol. 2009 Jan;103(1):53–6.

269. Katsanos KH, Katopodis KP, Kosmidou M, Siamopoulos KC, Tsianos E V. Leptospira interrogans icterhaemorrhagiae in a patient with Crohns disease. Vol. 16, Inflammatory Bowel Diseases. 2010. p. 1824–5.

270. Windpessl M, Prammer W, Nömeyer R, Dinkhauser P, Wimmer L, Müller P, et al. Leptospirosis and renal failure: a case series. Wien Klin Wochenschr [Internet]. 2014;126(7–8):238–42. Available from: https://dx.doi.org/10.1007/s00508-014-0501-0

271. Navarro-Torres M, Neugarten J. Leptospirosis: An Underrecognized Cause of AKI. American Journal of Kidney Diseases. 2022 Apr;79(4 (Supplement 2)):S76.

272. Wang HJ, Chen GZ, Zhou CJ, Fu Y, Yao LN. Veno-venous extracorporeal membrane oxygenation successfully treated a case of severe pulmonary hemorrhage caused by leptospirosis. BMC Infect Dis. 2020 Dec 1;20(1).

273. van de Weyer RWL, Ramakers BP, Pickkers P. Leptospirosis. Ned Tijdschr Geneeskd [Internet]. 2015;159:A7797. Available from: http://www.ncbi.nlm.nih.gov/pubmed/25740183

274. Pimenta D, Democratis J. Risky behaviour: a rare complication of an uncommon disease in a returning traveller. BMJ Case Rep [Internet]. 2013 Oct 9;2013. Available from: http://www.ncbi.nlm.nih.gov/pubmed/24108774

275. Balakrishnan T, Moore A, Krishnamoorthy S, Davies G, Lee PKH. Weil’s disease presenting as atypical pneumonia. Acute Medicine. 2014;13(4):178–81.

276. Alian S, Taghipour M, Sharifian R, Fereydouni MA. Cavernous sinus thrombosis syndrome and brainstem involvement in patient with leptospirosis: Two rare complications of leptospirosis. Journal of Research in Medical Sciences. 2014;907.

277. Connor-Schuler R, Khan A, Goyal N, Zimny E. Pressor support during a Jarisch Herxheimer reaction after initiation of treatment for Weil’s disease. American Journal of Emergency Medicine. 2017 Aug 1;35(8):1211.e3-1211.e4.

278. Kallel H, Bourhy P, Mayence C, Houcke S, Hommel D, Picardeau M, et al. First report of human Leptospira santarosai infection in French Guiana. J Infect Public Health. 2020 Aug 1;13(8):1181–3.

279. Lecadieu A, Veyret S, Persichini R, Duarte L, Caron M, Vidal C, et al. Case Report: Refractory Acute Respiratory Distress Syndrome Supported by Extracorporeal Membrane Oxygenation due to Coinfection with Chlamydia pneumoniae and Leptospirosis in Reunion Island. American Journal of Tropical Medicine and Hygiene. 2021 Mar 1;104(3):866–7.

280. Sirak Petros, Ute Leonhardt, Lothar. Serum Procalcitonin and Proinflammatory Cytokines in a Patient with Acute Severe Leptospirosis. Scand J Infect Dis [Internet]. 2000 Jan 8;32(1):104–5. Available from: http://www.tandfonline.com/doi/full/10.1080/00365540050164362

281. Dall’Antonia M, Sluga G, Whitfield S, Teall A, Wilson P, Krahé D. Leptospirosis pulmonary haemorrhage: A diagnostic challenge. Emergency Medicine Journal. 2008 Jan;25(1):51–2.

282. Bismaya K, Dev P, Favas TT, Pathak A. Neuro-Leptospirosis: Experience from a tertiary center of North India. Rev Neurol (Paris). 2023 Mar 1;179(3):238–43.

283. Anupama YJ, Harish KR. WCN25-4523 Leptospirosis in patients on chronic haemodialysis – A case series. Kidney Int Rep [Internet]. 2025 Feb;10(2):S752. Available from: https://linkinghub.elsevier.com/retrieve/pii/S2468024924033576

284. Vilaichone RK, Mahachai V, Wilde H. Acute Acalculous Cholecystitis in Leptospirosis. J Clin Gastroenterol [Internet]. 1999 Oct;29(3):280–3. Available from: http://journals.lww.com/00004836-199910000-00012

285. Chedraui PA, San Miguel G. A case of leptospirosis and pregnancy. Arch Gynecol Obstet [Internet]. 2003 Nov;269(1):53–4. Available from: http://www.ncbi.nlm.nih.gov/pubmed/14605821

286. Stefos A, Georgiadou SP, Gioti C, Loukopoulos A, Ioannou M, Pournaras S, et al. Leptospirosis and pancytopenia: Two case reports and review of the literature. Journal of Infection. 2005 Dec;51(5).

287. Lin CY, Chiu NC, Lee CM. Leptospirosis after typhoon. American Journal of Tropical Medicine and Hygiene. 2012 Feb;86(2):187–8.

288. Krishnan A, Karnad DR, Medhekar TP. Paralysis due to renal potassium wasting: An unusual presentation of leptospirosis [5]. Nephrology Dialysis Transplantation. 2003 Nov;18(11):2454–5.

289. McLean M, Ruscoe Q, Kline T, King C, Nesdale A. A cluster of three cases of leptospirosis in dairy farm workers in New Zealand. N Z Med J [Internet]. 2014 Jan 24;127(1388):13–20. Available from: http://www.ncbi.nlm.nih.gov/pubmed/24481382

290. Koe SLL, Tan KT, Tan TC. Leptospirosis in pregnancy with pathological fetal cardiotocography changes. Singapore Med J. 2014 Feb;55(2).

291. de Sainte Marie B, Delord M, Dubourg G, Gautret P, Parola P, Brouqui P, et al. Leptospirosis presenting as honeymoon fever. International Journal of Infectious Diseases. 2015 May 1;34:102–4.

292. Liao CY, Ben RJ, Wu HM, Chang SK, Liu MY, Chin HK, et al. Acute respiratory distress syndrome manifested by leptospirosis successfully teated by extracorporeal membrane oxygenation (ECMO). Internal Medicine. 2015 Nov 15;54(22):2943–6.

293. Muthuppalaniappan VM, Rajakariar R, Blunden MJ. Leptospirosis presenting as haemolytic uraemic syndrome: A case report. BMC Nephrol. 2018 Jan 29;19(1).

294. Bhatt M, Rastogi N, Soneja M, Biswas A. Uncommon manifestation of leptospirosis: A diagnostic challenge. BMJ Case Rep. 2018;2018.

295. Chen TL, Wang L shinn. Leptospiral presentation in Behçet’s disease: A case report. Journal of the Formosan Medical Association. 2020 Mar 1;119(3):743–6.

296. Coursin DB, Updike SJ, Maki DG. Massive rhabdomyolysis and multiple organ dysfunction syndrome caused by leptospirosis. Intensive Care Med [Internet]. 2000 Jun;26(6):808–12. Available from: http://link.springer.com/10.1007/s001340051252

297. Perez Garzón M, Zarama-Eraso MA, Sánchez Herrera P, Tipasoca Pineda J. Atypical clinical debut associated with Jarisch Herxheimer reaction in an asplenic patient with leptospirosis: case report and review. BMC Infect Dis. 2024 Dec 1;24(1).

298. Sheng Y. Role of hydrocortisone of large dosage in treatment of leptospirosis by penicillin- clinical observation of 130 cases. Chinese journal of practical internal medicine. 1984;(2):19–20.
