# Supplementary material for: Efficacy and safety outcomes reported in human leptospirosis studies to inform the development of a core outcome and core outcome measurement set: A systematic review
Source: PLoS Negl Trop Dis. 2026 Jul 13;20(7):e0013651. doi: 10.1371/journal.pntd.0013651 (PMC13395454; doi:10.1371/journal.pntd.0013651)
Supplement: S7 Appendix — (DOCX) [file pntd.0013651.s007.docx]

| **S7 Appendix. Summary of reporting region, design of study, reported outcome domains, and antibiotic class by studies published <2000, 2000 – 2019, and ≥2020** | | | |
| --- | --- | --- | --- |
|  | **Year categories** | | |
|  | **<2000**  **(n=27)** | **2000 – 2019**  **(n=194)** | **≥2020**  **(n=77)** |
| Region reporting studies |  |  |  |
| South East Asia | 3 (11.1) | 10 (5.2) | 10 (13.0) |
| Europe | 4 (14.8) | 55 (28.4) | 12 (15.6) |
| South Asia | -- | 40 (20.6) | 19 (24.7) |
| Middle East | 1 (3.7) | 9 (4.6) | 1 (1.3) |
| North America | 3 (11.1) | 12 (6.2) | 4 (5.2) |
| Africa | 2 (7.4) | 4 (2.1) | 3 (3.9) |
| Latin America (including Caribbean) | 8 (29.6) | 37 (19.1) | 16 (20.8) |
| East Asia | 4 (14.8) | 21 (10.8) | 10 (13.0) |
| Oceania | 2 (7.4) | 6 (3.1) | 2 (2.6) |
|  |  |  |  |
| Design type of study |  |  |  |
| Interventional | 9 (33.3) | 15 (7.7) | -- |
| Observational | 3 (11.1) | 45 (23.2) | 22 (28.6) |
| Systematic Review with narrative analysis | -- | 6 (3.1) | 7 (9.1) |
| Surveillance | 2 (7.4) | 1 (0.5) | 3 (3.9) |
| Modelling | -- | 2 (1.0) | 1 (1.3) |
| Economic Evaluation | -- | 1 (0.5) | -- |
| Case report | 10 (37.0) | 104 (53.6) | 27 (35.1) |
| Case series | 3 (11.1) | 9 (4.6) | 2 (2.6) |
| Systematic Review with meta analysis | -- | 3 (1.6) | 8 (10.4) |
| Trial/study registry | -- | 7 (3.6) | 8 (10.4) |
| Trial/study protocol | -- | 1 (0.5) | 1 (1.3) |
|  |  |  |  |
| Outcomes reported by domain* |  |  |  |
| **Death** | 15 (55.6) | 110 (56.7) | 42 (54.6) |
| **Physiological/Clinical** |  |  |  |
| Renal outcomes | 10 (37.0) | 55 (28.4) | 14 (18.2) |
| General outcomes | 6 (22.2) | 42 (21.7) | 13 (16.9) |
| Infection outcomes | 9 (33.3) | 29 (15.0) | 22 (28.6) |
| Hepatic outcomes | 5 (18.5) | 37 (19.1) | 8 (10.4) |
| Respiratory outcomes | 5 (18.5) | 36 (18.6) | 8 (10.4) |
| Blood outcomes | 4 (14.8) | 33 (17.0) | 6 (7.8) |
| Nervous system outcomes | 4 (14.8) | 18 (9.3) | 5 (6.5) |
| Cardiac outcomes | -- | 13 (6.7) | 2 (2.6) |
| Gastroenterological outcomes | 2 (7.4) | 6 (3.1) | 1 (1.3) |
| Immune system outcomes | -- | 3 (1.6) | 4 (5.2) |
| Pregnancy outcomes | 1 (3.7) | -- | 4 (5.2) |
| Eye outcomes | 1 (3.7) | 1 (0.5) | -- |
| Ear outcomes | -- | -- | 1 (1.3%) |
| Musculoskeletal outcomes | -- | 1 (0.5) | -- |
| **Resource Use** |  |  |  |
| Hospital outcomes | 13 (48.2) | 101 (52.1) | 46 (59.7) |
| Need for further intervention outcomes | 2 (7.4) | 53 (27.3) | 19 (24.7) |
| Economic outcomes | -- | 4 (2.1) | 2 (2.6) |
| **Adverse outcomes** | 3 (11.1) | 20 (10.3) | 11 (14.3) |
| **Life impact outcomes** |  |  |  |
| Quality of life outcomes | -- | 1 (0.5) | 5 (6.5) |
| Personal circumstances outcomes | -- | 1 (0.5) | 5 (6.5) |
| Delivery of care outcomes | -- | 1 (0.5) | 2 (2.6) |
| Cognitive functioning outcomes | -- | -- | 1 (1.3) |
|  |  |  |  |
| Antibiotics reported by class (n=222) |  |  |  |
| Penicillin | 15 (68.2) | 74 (49.3) | 17 (34.0) |
| Penicillin and beta-lactamase inhibitor | -- | 15 (10.0) | 7 (14.0) |
| Macrolide | 1 (4.6) | 16 (10.7) | 14 (28) |
| Cephalosporin | 3 (13.6) | 70 (46.7) | 35 (70.0) |
| Chloramphenicol | 1 (4.6) | 4 (2.7) | 3 (6.0) |
| Fluoroquinolone | 1 (4.6) | 18 (12.0) | 4 (8.0) |
| Tetracycline | 11 (50.0) | 51 (34.0) | 30 (60.0) |
| Carbapenem | -- | 10 (6.7) | 10 (20.0) |
| Aminoglycoside | 2 (9.1) | 3 (2.0) | -- |
| Nitroimidazole | 1 (4.6) | 2 (1.3) | -- |
| Glycopeptide | -- | 4 (2.7) | 3 (6.0) |
| Diaminopyrimidines  /sulfonamide | 1 (4.6) | 1 (0.7) | -- |
| Oxazolidinone | -- | -- | 1 (2.0) |
| *percentages do not add up to 100% as multiple outcomes can be reported by study | | | |
