# Supplementary material for: Efficacy and safety outcomes reported in human leptospirosis studies to inform the development of a core outcome and core outcome measurement set: A systematic review
Source: PLoS Negl Trop Dis. 2026 Jul 13;20(7):e0013651. doi: 10.1371/journal.pntd.0013651 (PMC13395454; doi:10.1371/journal.pntd.0013651)
Supplement: S8 Appendix — (DOCX) [file pntd.0013651.s008.docx]

| **Supplemental Document 8 - Tabulated summary of antibiotics reported by studies** | | | | |
| --- | --- | --- | --- | --- |
| **Antibiotic class**  **% (N)** | **Antibiotic type** | **N (% of class)** | **Antibiotic type** | **N (% of class)** |
| **Penicillin**  48.6% (108) | Amoxicillin | 10 (9.3%) | Flucloxacillin | 1 (0.9%) |
|  | Ampicillin | 17 (15.7%) | Penicillin | 93 (86.1%) |
| **Penicillin + β-lactamase inhibitor**  9.9% (22) | Ampicillin/Sulbactam | 6 (27.3%) | Piperacillin/Tazobactam | 17 (77.3%) |
|  | Amoxicillin/Clavulanic Acid | 7 (31.8%) | Piperacillin/Sulbactam | 1 (4.5%) |
| **Macrolide**  14.0% (31) | Erythromycin | 1 (32.3%) | Spiramycin | 1 (32.3%) |
|  | Clarithromycin | 6 (19.4%) | Macrolide (not specified)) | 2 (6.5%) |
|  | Azithromycin | 17 (54.8%) |  |  |
| **Cephalosporin**  49.1% (109) | Ceftriaxone | 98 (89.9%) | Cefoperazone | 1 (0.9%) |
|  | Cefotaxime | 13 (11.9%) | Cefixime | 1 (0.9%) |
|  | Ceftazidime | 4 (3.7%) | 3^rd^ generation cefalosporin (non-specific) | 2 (1.8%) |
|  | Cefepime | 2 (1.8%) | Cefalosporin (not specified) | 2 (1.8%) |
|  | Cefalothin | 1 (0.9%) |  |  |
| **Carbapenem**  9.0% (20) | Meropenem | 18 (90.0%) | Imipenem | 2 (10.0%) |
|  | Carbapenem (non-specific) | 1 (0.5%) |  |  |
| **Beta-lactam (non-specific)**  0.9% (2) |  | | | |
| **Tetracycline**  41.4% (92) | Tetracycline | 4 (4.3%) | Oxytetracycline | 3 (3.3%) |
|  | Doxycycline | 85 (92.4%) | Minocycline | 1 (1.1%) |
|  | Omadacycline | 1 (1.1%) |  |  |
| **Fluoroquinolone**  10.4% (23) | Ciprofloxacin | 11 (47.8%) | Moxifloxacin | 3 (13.0%) |
|  | Levofloxacin | 8 (34.8%) | Fluoroquinolone (non-specific) | 2 (8.7%) |
| **Nitroimidazole**  1.4% (3) | Metronidazole |  |  |  |
| **Glycopeptide**  3.2% (7) | Vancomycin |  |  |  |
| **Diaminopyrimidines**  **/sulfonamide**  0.9% (2) | Co-trimoxazole |  |  |  |
| **Chloramphenicol**  3.6% (8) |  | | | |
| **Aminoglycoside**  2.3% (5) | Amikacin |  |  |  |
| **Oxazolidinone**  0.5% (1) | Linezolid |  |  |  |

Number of studies reporting antibiotics = 222. Sum of studies reported per antibiotic may not equal number reported per antibiotic class as studies may report one more than one antibiotic used.
