# Supplementary material for: Efficacy and safety outcomes reported in human leptospirosis studies to inform the development of a core outcome and core outcome measurement set: A systematic review
Source: PLoS Negl Trop Dis. 2026 Jul 13;20(7):e0013651. doi: 10.1371/journal.pntd.0013651 (PMC13395454; doi:10.1371/journal.pntd.0013651)
Supplement: Attachment — Submitted filename: Response_to_reviewers.docx [file pntd.0013651.s010.docx]

| **Journal Requirements** |  |
| --- | --- |
| 1) We do not publish any copyright or trademark symbols that usually accompany proprietary names, eg ©,  ®, or TM  (e.g. next to drug or reagent names). Therefore please remove all instances of trademark/copyright symbols throughout the text, including: - ® on page: 16.. | This has now been removed. |
| 2) We have noticed that you have uploaded Supporting Information files, but you have not included a list of legends. Please add a full list of legends for your Supporting Information files after the references list. | These have now been added. |
| 3) Some material included in your submission may be copyrighted. According to PLOSu2019s copyright policy, authors who use figures or other material (e.g., graphics, clipart, maps) from another author or copyright holder must demonstrate or obtain permission to publish this material under the Creative Commons Attribution 4.0 International (CC BY 4.0) License used by PLOS journals. Please closely review the details of PLOSu2019s copyright requirements here: PLOS Licenses and Copyright. If you need to request permissions from a copyright holder, you may use PLOS's Copyright Content Permission form. Please respond directly to this email and provide any known details concerning your material's license terms and permissions required for reuse, even if you have not yet obtained copyright permissions or are unsure of your material's copyright compatibility. Once you have responded and addressed all other outstanding technical requirements, you may resubmit your manuscript within Editorial Manager.  Potential Copyright Issues:  - Figure 2. Please (a) provide a direct link to the base layer of the map (i.e., the country or region border shape) and ensure this is also included in the figure legend; and (b) provide a link to the terms of use / license information for the base layer image or shapefile. We cannot publish proprietary or copyrighted maps (e.g. Google Maps, Mapquest) and the terms of use for your map base layer must be compatible with our CC BY 4.0 license. Note: if you created the map in a software program like R or ArcGIS, please locate and indicate the source of the basemap shapefile onto which data has been plotted. If your map was obtained from a copyrighted source please amend the figure so that the base map used is from an openly available source. Alternatively, please provide explicit written permission from the copyright holder granting you the right to publish the material under our CC BY 4.0 license. If you are unsure whether you can use a map or not, please do reach out and we will be able to help you. The following websites are good examples of where you can source open access or public domain maps: * U.S. Geological Survey (USGS) - All maps are in the public domain. (http://www.usgs.gov) * PlaniGlobe - All maps are published under a Creative Commons license so please cite u201cPlaniGlobe, http://www.planiglobe.com, CC BY 2.0u201d in the image credit after the caption. (http://www.planiglobe.com/?lang=enl) * Natural Earth - All maps are public domain. (<http://www.naturalearthdata.com/about/terms-of-use/>). | This map was created using QGIS utilising data provided by Natural Earth ([www.naturalearthdata.com](http://www.naturalearthdata.com)). Terms of use are found [here](https://www.naturalearthdata.com/about/terms-of-use/). The figure legend has been updated. |
| 4) Please amend your detailed Financial Disclosure statement. This is published with the article. It must therefore be completed in full sentences and contain the exact wording you wish to be published. State the initials, alongside each funding source, of each author to receive each grant. For example: "This work was supported by the National Institutes of Health (####### to AM; ###### to CJ) and the National Science Foundation (###### to AM).". If you did not receive any funding for this study, please simply state: u201cThe authors received no specific funding for this work.u201d | This will be amended directly on the submission system. Please note that the funding program does not have a grant number attached. |
| We have amended your Competing Interest statement to comply with journal style. We kindly ask that you double check the statement and let us know if anything is incorrect. | These have been reviewed and we find no issue with this. Thank you very much. |
| The file inventory includes files for Figures 3a to 3f. We would recommend either combining these into a single Figure file with separate internal panels, or renumbering them as individual figures , as we are not able to publish multiple components of a single figure as separate files.  NOTE: Please ensure that all included figures are referenced to ensure that your paper is fully reviewed. | As we would like to keep the resolution of each of the sunburst chart images, we have decided to organise the images as separate Figure files. |
| Thank you for stating"The complete cleaned summary dataset is available at DOI:10.5281/zenodo.17148991. " | This repository status of this has been activated and should now be searchable and accessible on Zenodo. |
| **Reviewer comments on Methods** |  |
| Reviewer #1: yes the methodology is acceptable but it would be interesting to note what number of papers were from non English literature. study design is appropriate the hypothesis is clear and appropriate sample size: Not applicable here analysis is adequate | Thank you for this suggestion. We have now incorporated the language breakdown into the **Table 1** characteristics of studies. |
| Reviewer #2: The study applies standardised systematic review methods. The study investigates hospitalised patients and attempts to develop a controlled ontology for description of clinical outcomes. | Thank you. |
| Reviewer #3: The objectives of the study are clearly articulated with a clear testable hypothesis stated The study design is appropriate to address the stated objectives | Thank you. |
| **Reviewer comments on Results** |  |
| Reviewer #1: analysis can be improved i would request the authors to please divide the literature available into 3 groups, before the year 2000, year 2000 till 2019, year 2020 till date and provide the difference in methods used for diagnosis and its implication on the final diagnosis. how many cases may be missed by serology or microscopic detection. the outcome measure and the antibiotic selection is also likely to be different in these 3 time periods. it is possible that before the year 2000, mortality might have been the indicators, where as subsequently organ system dysfunction, time to discharge, duration of ICU stay may be outcome measures. it would be interesting to build up this story of changing paradigms of outcome here over the decades. it would be interesting if the entire SE Asian region is combines and compared with other regions during these periods | Thank you for your comment and recommendations. While we agree completely regarding the importance of documenting changes in diagnostics methods for leptospirosis over time, the primary objective of this systematic review was to broadly catalogue the reported outcomes and outcome measures across the literature to inform the development of a core outcome set. To undertake an accurate analysis of diagnostic accuracy would require methodological data that is inconsistently reported across the wide variety of included studies, and falls outside the predefined scope of this review.  We also have concerns that exploring in detail the change in reported outcomes over time or when contrasting different regions, while providing insights into evolving research priorities and reporting patterns in human leptospirosis, would be taken to imply that they are contributory to a hierarchy of importance among outcomes.  However, to address the important points that your raise, we have decided to summarise the data on reporting region, design of study, reported outcome domains, and antibiotic classes as reported by studies published in the year categories that you have suggested. This information is presented in **Supplemental Document 5**, and is also referred to directly in the manuscript’s “General characteristics” section paragraph 2 (line 198 – 204); “Reported Outcomes” section paragraph 2 (line 255 – 258), paragraph 3 (line 265 – 268), paragraph 4 (line 271 – 272), paragraph 5 (line 275 – 276), paragraph 6 (line 285 – 286); and “Antibiotic interventions” section paragraph 1 (line 336 – 344) |
| Reviewer #2: Yes to all | Thank you. |
| Reviewer #3: The results are clearly and completely presented The figures (Tables, Images) are of sufficient quality for clarity | Thank you. |
| **Reviewer comments on Conclusion** |  |
| Reviewer #1: conclusions are clear as far as the data interpretation is concerned. i donot think that this study touches on public health aspects. | Thank you for your comment. We agree that the limited reporting of quality-of-life and economic outcomes likely reflects the relative under-representation of public health considerations in leptospirosis research. However, a more expansive commentary of broader public health implications is beyond the scope of this systematic review which focuses on cataloguing reported outcomes to inform the development of a core outcome set for use in clinical trials. |
| Reviewer #2: Yes to all | Thank you. |
| Reviewer #3: The conclusions are supported by the data presented | Thank you. |
| **Reviewer comment on Editorial and Data Presentation Modifications** |  |
| Reviewer #1: i would request the authors to please divide the literature available into 3 groups, before the year 2000, year 2000 till 2019, year 2020 till date and provide the difference in methods used for diagnosis and its implication on the final diagnosis. how many cases may be missed by serology or microscopic detection. the outcome measure and the antibiotic selection is also likely to be different in these 3 time periods. it is possible that before the year 2000, mortality might have been the indicators, where as subsequently organ system dysfunction, time to discharge, duration of ICU stay may be outcome measures. it would be interesting to build up this story of changing paradigms of outcome here over the decades. it would be interesting if the entire SE Asian region is combines and compared with other regions during these periods. it would be interesting to know the outcomes changing according to antibiotics choices. | Thank you for your comment. We hope that we have addressed this for you in the peer review section regarding our Results.  We note the additional comment here regarding outcomes changing according to antibiotic choices. While analysing outcomes according to antibiotics would be informative, the available data are not suitable for such comparisons given the predominance of descriptive studies, lack of standardised outcome definitions, and inconsistent reporting of treatment regimens or justifications for their use. However, we allude to the need for this in future studies in the “Limitations and deviations from protocol” section paragraph 2 (line 594 – 598). |
| Reviewer #2: Not applicable | Thank you. |
| Reviewer #3: No | Thank you. |
| **Reviews comments in Summary and General Comments** |  |
| Reviewer #1: In my view, these changes would give us more realistic picture of the leptospirosis story . | Many thanks again for your insightful commentary. |
| Reviewer #2: The manuscript provides a list of categorised outcomes and antibiotics used in a curated list of leptospirosis studies. The main finding presented is that non standardised ontologies are used and that this needs to be improved. The authors mention the WHO guidelines for leptospirosis - what are the recommended antibiotics for treatment? How does that differ from usage in the studies? Is AMR a problem? - unlikely due to the diverse zoonotic sources. The study catalogues the antibiotics used at a study level, would it be more appropriate to look at the number of patients? Is severe leptospirosis resulting in hospitalisation associated with delay in initial diagnosis/delay in seeking medical attention (possible socio-economic factor in LMICs) worth looking into? Is antibiotic therapy ineffective once organ/system shutdown has commenced? How effective is antibiotic therapy for leptospirosis otherwise? (with WHO recommended antibiotics). No mention of disease severity associated with species of Leptospira or serovar - Should this be included as part of the information collected? | Thank you for your insights. We appreciate the opportunity to clarify several of the points you have raised.  We would like to provide some context to our responses to your queries. Our review was primarily designed to systematically catalogue and categorise reported outcomes and their measures to inform the development of a core outcome set, rather than to evaluate treatment effectiveness, disease prognosis, or clinical determinants of severity. Although the points you raise are valid and areas of leptospirosis research where there is critical need of focus, some of these slightly fall outside the predefined scope and therefore the data extraction framework used for this review. However, we would like to respond in detail to each of your queries in turn below.  The antibiotic recommendations based on the most recent WHO guidelines are based on disease severity. In severe cases, the guidance states that leptospirosis should be treated with high doses of intravenous penicillin. Less severe cases may be treated with “oral antibiotics such as amoxicillin, ampicillin, doxycycline, or erythromycin”. Third generation cephalosporins, such as ceftriaxone and cefotaxime, and quinolones may “also appear to be effective”. It should be noted that the WHO guidelines do not provide a comprehensive review of the evidence underlying these recommendations. We report in our results that the most three most frequently reported antibiotic classes – penicillin, cephalosporins, and tetracyclines – are generally aligned with WHO recommendations. However, there are some reported intra-class variations which do not align with these. We also report that almost half of included studies reported using antibiotics with no previous existing trial or guidance evidence to support their use. We have updated paragraph 2 (line 544-548) in the “Treatment Strategies” section of the Discussion to better describe existing WHO guidance.  With respect to antimicrobial resistance, there is currently no documented incidence of clinically significant resistance amongst *Leptospira spp*. A key limitation in our knowledge is that there is no methodological consensus for phenotypic susceptibility testing. Consequently, resistance pattern testing was not systematically reported (if at all) in included studies. We have added a brief statement in the second paragraph of the “Treatment section” (line 599 – 563) in our Discussion to reflect this.  Summary reporting or further analysis at the patient level (e.g. number of patients treated with *x* antibiotic, manifestations of disease severity associated with delay in diagnosis/hospital presentation, disease severity associated with serovar/species) lies outside the scope of this review. However, robust data collection summarising these characteristics would not have been feasible due to the wide variety of designs of included studies, as well as substantial variability in granular accounting of treatments or clinical characteristics at the individual patient level between studies. We have included a statement in the “Limitations and deviations from protocol” section of the Discussion (line 595 - 598) summarising this aspect.  As we have mentioned above, our study design and information capture was unable to comment on treatment effectiveness of antimicrobials or other pharmacological and non-pharmacological treatments.  We hope this clarifies the focus of the review and the rationale for the analyses undertaken. |
| Reviewer #3: This manuscript presents a large systematic review of efficacy and safety outcomes reported in human leptospirosis studies, with the objective of informing the development of a core outcome set. The authors synthesise data from 298 studies across 63 countries and apply recognised methodological standards (PRISMA, COMET, PROSPERO). The review clearly demonstrates extensive heterogeneity in outcome reporting, outcome measures, and treatment strategies, and highlights major gaps in patient-centred and harms outcomes. Overall, this is a timely and valuable contribution that addresses an important barrier to evidence-based leptospirosis research. Major Strengths • Exceptional breadth and global coverage, including a wide range of study designs. • Clear demonstration of outcome heterogeneity across all COMET core areas. • Novel synthesis of outcome measures and treatment practices not previously collated. Major Comments 1. Influence of descriptive studies Over half of included studies are descriptive (case reports/series), which likely overemphasises clinician-prioritised and severe outcomes. While acknowledged, the manuscript would benefit from a clearer justification in the Methods and a more explicit discussion of how this may shape the observed outcome landscape and COS development. 2. Outcome grouping and domain assignment Outcomes and domains were derived inductively. Please clarify whether outcome grouping and mapping to COMET domains were independently reviewed or discussed among authors, and how disagreements were resolved. 3. Patient-reported outcomes The near-complete absence of patient-reported outcomes is a key finding. The Discussion should more explicitly state that qualitative research and patient engagement will be essential in subsequent COS development phases, and clarify whether this is planned within COS-LEP. 4. Outcome reporting bias assessment ORBIT assessments were conducted by a single reviewer. Please clarify whether any calibration or secondary checking was undertaken, and briefly discuss how outcome reporting bias may affect COS formulation. 5. Interpretation of treatment heterogeneity The extensive variability in antimicrobial and adjunctive therapies is well described. To avoid overinterpretation, the discussion should clearly state that these findings contextualise outcome variability rather than evaluate treatment effectiveness. | Thank you for your thoughtful and constructive comments. We have addressed each point below.   1) Influence of descriptive studies   The decision to include a broad range of study designs in the systematic review was prespecified in the protocol of the wider core outcome set development for leptospirosis trials (COS-LEP) project. Given the limited high-quality treatment evidence in leptospirosis, our intention was for the core outcome development process to be as comprehensive as possible at each stage. As a neglected infectious disease, we anticipated that high quality analytical studies would be relatively few, and where available, may report a limited set of repeatedly measured outcomes.  The inclusion of descriptive studies was therefore intentional to ensure comprehensive identification of outcomes reported in the literature consistent with established COMET methodology for outcome mapping in core outcome set development. Case reports and series often report rare, unexpected, or clinically important outcomes which may not be captured in other study designs with less clinical focus. These may also provide insights into uncommon or severe manifestations of disease. Restricting inclusion to analytical designs alone could introduce a selection bias toward more investigator-selected endpoints, and risk overlooking outcomes relevant to clinical practice.  We have now added sections to reflect the above justifications. The first is in the second paragraph of the “Study search strategy and selection” section of the Methods (line 136-139) to justify our approach. The second is an expansion of paragraph 3 in the “Outcome reporting” section (line 436 – 443) of the Discussion.   2) Outcome grouping and domain assignment   Outcome grouping and mapping to COMET domains were discussed between study authors as well as being reviewed at the COS-LEP steering committee meeting. There were fortunately no disagreements regarding classifications, but if there had been this would have been resolved by discussion between authors, and where necessary, at steering committee meetings. We have added a sentence in the first paragraph of the “Data Synthesis and Analysis” section (line 187 -189) to describe this.   3) Patient reported outcomes   We agree that the paucity of patient-reported outcomes is an important finding. The COS-LEP project did include a separate qualitative study exploring treatment outcomes directly with stakeholder groups, including patients and healthcare providers, to identify outcomes of importance from lived experience perspectives. We have updated paragraph 4 of the “Outcome reporting” section (line 444 – 457) and the Conclusion paragraph (line 604 – 609) to emphasise the need for further qualitative research as suggested.   4) Outcome reporting bias assessment   Both ORBIT (outcome reporting bias) and QualSyst (study quality) assessments were conducted by the lead author. We acknowledge that single-reviewer assessment may have introduced some subjectivity. However, as pre-specified in the COS-LEP protocol, these assessments were undertaken to provide context regarding the characteristics and reporting practices of included studies, rather than to inform study exclusion. The primary objective of this stage of the core outcome set development process was to achieve the broadest possible identification of outcomes and outcome measures across the literature. Excluding studies on the basis of methodological quality would have risked omitting potentially relevant outcomes and introduced a selection bias, which would be inconsistent with the aims of outcome generation in COS development.  We have added a statement in paragraph 1 as well as a new third paragraph of the “Quality and Risk of Bias Assessment” section of the Discussion (lines 408-409 and lines 416-420) to emphasise the above.   5) Interpretation of treatment heterogeneity   This is a very good point to make, and we agree that antimicrobial and adjunctive therapies summaries in this review should not be interpreted as evidence of treatment effectiveness.  We have included a statement in the first paragraph of the “Treatment strategies” section of the Discussion (line 542 – 543) to explicitly state this. |

**Reviewers' Comments:**

PLOS authors have the option to publish the peer review history of their article ([what does this mean?](https://track.editorialmanager.com/CL0/https:%2F%2Fjournals.plos.org%2Fplosntds%2Fs%2Feditorial-and-peer-review-process%23loc-peer-review-history/1/010f019b96bfc8ac-8b41753b-8847-41e3-a723-8a3062b7cbd2-000000/r0sNHvwG-533yRHEvKBD8N6oYf7t63QP2FavW8wkrCI=245)). If published, this will include your full peer review and any attached files.


If you choose “no”, your identity will remain anonymous but your review may still be made public.


**Do you want your identity to be public for this peer review?** For information about this choice, including consent withdrawal, please see our [Privacy Policy](https://track.editorialmanager.com/CL0/https:%2F%2Fwww.plos.org%2Fprivacy-policy/1/010f019b96bfc8ac-8b41753b-8847-41e3-a723-8a3062b7cbd2-000000/KgYP2gO2pPF5k5Egnv4__mf2UJhZ2SD_8QUCM_K0k6o=245).

Reviewer #1: **Yes:** Ashish Bhalla

Reviewer #2: No

Reviewer #3: No

[NOTE: If reviewer comments were submitted as an attachment file, they will be attached to this email and accessible via the submission site. Please log into your account, locate the manuscript record, and check for the action link "View Attachments". If this link does not appear, there are no attachment files.]

**Figure resubmission:**

While revising your submission, we strongly recommend that you use PLOS’s NAAS tool (https://ngplosjournals.pagemajik.ai/artanalysis) to test your figure files. NAAS can convert your figure files to the TIFF file type and meet basic requirements (such as print size, resolution), or provide you with a report on issues that do not meet our requirements and that NAAS cannot fix.

After uploading your figures to PLOS’s NAAS tool - https://ngplosjournals.pagemajik.ai/artanalysis, NAAS will process the files provided and display the results in the "Uploaded Files" section of the page as the processing is complete. If the uploaded figures meet our requirements (or NAAS is able to fix the files to meet our requirements), the figure will be marked as "fixed" above. If NAAS is unable to fix the files, a red "failed" label will appear above. When NAAS has confirmed that the figure files meet our requirements, please download the file via the download option, and include these NAAS processed figure files when submitting your revised manuscript.

**Reproducibility:**

To enhance the reproducibility of your results, we recommend that authors of applicable studies deposit laboratory protocols in protocols.io, where a protocol can be assigned its own identifier (DOI) such that it can be cited independently in the future. Additionally, PLOS ONE offers an option to publish peer-reviewed clinical study protocols. Read more information on sharing protocols at https://plos.org/protocols?utm_medium=editorial-email&utm_source=authorletters&utm_campaign=protocols
