# Supplementary material for: Efficacy and safety outcomes reported in human leptospirosis studies to inform the development of a core outcome and core outcome measurement set: A systematic review
Source: PLoS Negl Trop Dis. 2026 Jul 13;20(7):e0013651. doi: 10.1371/journal.pntd.0013651 (PMC13395454; doi:10.1371/journal.pntd.0013651)
Supplement: Peer review history

# Peer review: Decision Letter 0

**DOI:** 10.1371/journal.pntd.0013651.r001

**Authors:** Stuart Blacksell, Vasantha Neela

6 Jan 2026

PNTD-D-25-01839

Efficacy and safety outcomes reported in human leptospirosis studies to inform the development of a core outcome and core outcome measures set: a systematic review by the COS-LEP study group

PLOS Neglected Tropical Diseases

Dear Dr. Edwards,

Thank you for submitting your manuscript to PLOS Neglected Tropical Diseases. After careful consideration, we feel that it has merit but does not fully meet PLOS Neglected Tropical Diseases's publication criteria as it currently stands. Therefore, we invite you to submit a revised version of the manuscript that addresses the points raised during the review process.

Please submit your revised manuscript within by Mar 07 2026 11:59PM. If you will need more time than this to complete your revisions, please reply to this message or contact the journal office at plosntds@plos.org. When you're ready to submit your revision, log on to https://www.editorialmanager.com/pntd/ and select the 'Submissions Needing Revision' folder to locate your manuscript file.

Please include the following items when submitting your revised manuscript:

* A letter that responds to each point raised by the editor and reviewer(s). You should upload this letter as a separate file labeled 'Response to Reviewers'. This file does not need to include responses to any formatting updates and technical items listed in the 'Journal Requirements' section below.

* A marked-up copy of your manuscript that highlights changes made to the original version. You should upload this as a separate file labeled 'Revised Manuscript with Track Changes'.

* An unmarked version of your revised paper without tracked changes. You should upload this as a separate file labeled 'Manuscript'.

If you would like to make changes to your financial disclosure, competing interests statement, or data availability statement, please make these updates within the submission form at the time of resubmission. Guidelines for resubmitting your figure files are available below the reviewer comments at the end of this letter.

We look forward to receiving your revised manuscript.

Kind regards,

Vasantha Kumari Neela

Academic Editor

PLOS Neglected Tropical Diseases

Stuart Blacksell

Section Editor

PLOS Neglected Tropical Diseases

Shaden Kamhawi

co-Editor-in-Chief

PLOS Neglected Tropical Diseases

orcid.org/0000-0003-4304-636XX

Paul Brindley

co-Editor-in-Chief

PLOS Neglected Tropical Diseases

orcid.org/0000-0003-1765-0002

**Journal Requirements:**

1) We do not publish any copyright or trademark symbols that usually accompany proprietary names, eg ©,  ®, or TM  (e.g. next to drug or reagent names). Therefore please remove all instances of trademark/copyright symbols throughout the text, including:

- ® on page: 16..

2) We have noticed that you have uploaded Supporting Information files, but you have not included a list of legends. Please add a full list of legends for your Supporting Information files after the references list.

3) Some material included in your submission may be copyrighted. According to PLOSu2019s copyright policy, authors who use figures or other material (e.g., graphics, clipart, maps) from another author or copyright holder must demonstrate or obtain permission to publish this material under the Creative Commons Attribution 4.0 International (CC BY 4.0) License used by PLOS journals. Please closely review the details of PLOSu2019s copyright requirements here: PLOS Licenses and Copyright. If you need to request permissions from a copyright holder, you may use PLOS's Copyright Content Permission form.

Please respond directly to this email and provide any known details concerning your material's license terms and permissions required for reuse, even if you have not yet obtained copyright permissions or are unsure of your material's copyright compatibility. Once you have responded and addressed all other outstanding technical requirements, you may resubmit your manuscript within Editorial Manager.

Potential Copyright Issues:

- Figure 2. Please (a) provide a direct link to the base layer of the map (i.e., the country or region border shape) and ensure this is also included in the figure legend; and (b) provide a link to the terms of use / license information for the base layer image or shapefile. We cannot publish proprietary or copyrighted maps (e.g. Google Maps, Mapquest) and the terms of use for your map base layer must be compatible with our CC BY 4.0 license.

Note: if you created the map in a software program like R or ArcGIS, please locate and indicate the source of the basemap shapefile onto which data has been plotted.

If your map was obtained from a copyrighted source please amend the figure so that the base map used is from an openly available source. Alternatively, please provide explicit written permission from the copyright holder granting you the right to publish the material under our CC BY 4.0 license.

If you are unsure whether you can use a map or not, please do reach out and we will be able to help you. The following websites are good examples of where you can source open access or public domain maps:

* U.S. Geological Survey (USGS) - All maps are in the public domain. (http://www.usgs.gov)

* PlaniGlobe - All maps are published under a Creative Commons license so please cite u201cPlaniGlobe, http://www.planiglobe.com, CC BY 2.0u201d in the image credit after the caption. (http://www.planiglobe.com/?lang=enl)

* Natural Earth - All maps are public domain. (http://www.naturalearthdata.com/about/terms-of-use/).

4) Please amend your detailed Financial Disclosure statement. This is published with the article. It must therefore be completed in full sentences and contain the exact wording you wish to be published.

State the initials, alongside each funding source, of each author to receive each grant. For example: "This work was supported by the National Institutes of Health (####### to AM; ###### to CJ) and the National Science Foundation (###### to AM).".

If you did not receive any funding for this study, please simply state: u201cThe authors received no specific funding for this work.u201d

**Reviewers' Comments:**

Reviewer's Responses to Questions

**Key Review Criteria Required for Acceptance?**

As you describe the new analyses required for acceptance, please consider the following:

**Methods**

-Are the objectives of the study clearly articulated with a clear testable hypothesis stated?

-Is the study design appropriate to address the stated objectives?

-Is the population clearly described and appropriate for the hypothesis being tested?

-Is the sample size sufficient to ensure adequate power to address the hypothesis being tested?

-Were correct statistical analysis used to support conclusions?

-Are there concerns about ethical or regulatory requirements being met?

Reviewer #1: yes

the methodology is acceptable but it would be interesting to note what number of papers were from non English literature.

study design is appropriate

the hypothesis is clear and appropriate

sample size: Not applicable here

analysis is adeqaute

Reviewer #2: The study applies standardised systematic review methods. The study investigates hospitalised patients and attempts to develop a controlled ontology for description of clinical outcomes.

Reviewer #3: The objectives of the study are clearly articulated with a clear testable hypothesis stated

The study design is appropriate to address the stated objectives

**********

**Results**

-Does the analysis presented match the analysis plan?

-Are the results clearly and completely presented?

-Are the figures (Tables, Images) of sufficient quality for clarity?

Reviewer #1: analysis can be improved

i would request the authors to please divide the literature available into 3 groups, before the year 2000, year 2000 till 2019, year 2020 till date and provide the difference in methods used for diagnosis and its implication on the final diagnosis. how many cases may be missed by serology or microscopic detection.

the outcome measure and the antibiotic selection is also likely to be different in these 3 time periods. it is possible that before the year 2000, mortality might have been the indicators, where as subsequently organ system dysfunction, time to discharge, duration of ICU stay may be outcome measures.

it would be interesting to build up this story of changing paradigms of outcome here over the decades.

it would be interesting if the entire SE Asian region is combines and compared with other regions during these periods

Reviewer #2: Yes to all

Reviewer #3: The results are clearly and completely presented

The figures (Tables, Images) are of sufficient quality for clarity

**********

**Conclusions**

-Are the conclusions supported by the data presented?

-Are the limitations of analysis clearly described?

-Do the authors discuss how these data can be helpful to advance our understanding of the topic under study?

-Is public health relevance addressed?

Reviewer #1: conclusions are clear as far as the data interpretation is concerned.

i donot think that this study touches on public health aspects.

Reviewer #2: Yes to all

Reviewer #3: The conclusions are supported by the data presented

**********

**Editorial and Data Presentation Modifications?**

Use this section for editorial suggestions as well as relatively minor modifications of existing data that would enhance clarity. If the only modifications needed are minor and/or editorial, you may wish to recommend “Minor Revision” or “Accept”.

Reviewer #1: i would request the authors to please divide the literature available into 3 groups, before the year 2000, year 2000 till 2019, year 2020 till date and provide the difference in methods used for diagnosis and its implication on the final diagnosis. how many cases may be missed by serology or microscopic detection.

the outcome measure and the antibiotic selection is also likely to be different in these 3 time periods. it is possible that before the year 2000, mortality might have been the indicators, where as subsequently organ system dysfunction, time to discharge, duration of ICU stay may be outcome measures.

it would be interesting to build up this story of changing paradigms of outcome here over the decades.

it would be interesting if the entire SE Asian region is combines and compared with other regions during these periods.

it would be interesting to know the outcomes changing according to antibiotics choices.

Reviewer #2: Not applicable

Reviewer #3: No

**********

**Summary and General Comments**

Use this section to provide overall comments, discuss strengths/weaknesses of the study, novelty, significance, general execution and scholarship. You may also include additional comments for the author, including concerns about dual publication, research ethics, or publication ethics. If requesting major revision, please articulate the new experiments that are needed.

Reviewer #1: In my view, these changes would give us more realistic picture of the leptospirosis story .

Reviewer #2: The manuscript provides a list of categorised outcomes and antibiotics used in a curated list of leptospirosis studies. The main finding presented is that non standardised ontologies are used and that this needs to be improved. The authors mention the WHO guidelines for leptospirosis - what are the recommended antibiotics for treatment? How does that differ from usage in the studies? Is AMR a problem? - unlikely due to the diverse zoonotic sources. The study catalogues the antibiotics used at a study level, would it be more appropriate to look at the number of patients? Is severe leptospirosis resulting in hospitalisation associated with delay in initial diagnosis/delay in seeking medical attention (possible socio-economic factor in LMICs) worth looking into? Is antibiotic therapy ineffective once organ/system shutdown has commenced? How effective is antibiotic therapy for leptospirosis otherwise? (with WHO recommended antibiotics). No mention of disease severity associated with species of Leptospira or serovar - Should this be included as part of the information collected?

Reviewer #3: This manuscript presents a large systematic review of efficacy and safety outcomes reported in human leptospirosis studies, with the objective of informing the development of a core outcome set. The authors synthesise data from 298 studies across 63 countries and apply recognised methodological standards (PRISMA, COMET, PROSPERO). The review clearly demonstrates extensive heterogeneity in outcome reporting, outcome measures, and treatment strategies, and highlights major gaps in patient-centred and harms outcomes. Overall, this is a timely and valuable contribution that addresses an important barrier to evidence-based leptospirosis research.

Major Strengths

• Exceptional breadth and global coverage, including a wide range of study designs.

• Clear demonstration of outcome heterogeneity across all COMET core areas.

• Novel synthesis of outcome measures and treatment practices not previously collated.

Major Comments

1. Influence of descriptive studies

Over half of included studies are descriptive (case reports/series), which likely overemphasises clinician-prioritised and severe outcomes. While acknowledged, the manuscript would benefit from a clearer justification in the Methods and a more explicit discussion of how this may shape the observed outcome landscape and COS development.

2. Outcome grouping and domain assignment

Outcomes and domains were derived inductively. Please clarify whether outcome grouping and mapping to COMET domains were independently reviewed or discussed among authors, and how disagreements were resolved.

3. Patient-reported outcomes

The near-complete absence of patient-reported outcomes is a key finding. The Discussion should more explicitly state that qualitative research and patient engagement will be essential in subsequent COS development phases, and clarify whether this is planned within COS-LEP.

4. Outcome reporting bias assessment

ORBIT assessments were conducted by a single reviewer. Please clarify whether any calibration or secondary checking was undertaken, and briefly discuss how outcome reporting bias may affect COS formulation.

5. Interpretation of treatment heterogeneity

The extensive variability in antimicrobial and adjunctive therapies is well described. To avoid overinterpretation, the discussion should clearly state that these findings contextualise outcome variability rather than evaluate treatment effectiveness.

**********

PLOS authors have the option to publish the peer review history of their article (what does this mean?). If published, this will include your full peer review and any attached files.

If you choose “no”, your identity will remain anonymous but your review may still be made public.

**Do you want your identity to be public for this peer review?**  For information about this choice, including consent withdrawal, please see our Privacy Policy.

Reviewer #1: **Yes:** Ashish Bhalla

Reviewer #2: No

Reviewer #3: No

[NOTE: If reviewer comments were submitted as an attachment file, they will be attached to this email and accessible via the submission site. Please log into your account, locate the manuscript record, and check for the action link "View Attachments". If this link does not appear, there are no attachment files.]

**Figure resubmission:**

While revising your submission, we strongly recommend that you use PLOS’s NAAS tool (https://ngplosjournals.pagemajik.ai/artanalysis) to test your figure files. NAAS can convert your figure files to the TIFF file type and meet basic requirements (such as print size, resolution), or provide you with a report on issues that do not meet our requirements and that NAAS cannot fix.

After uploading your figures to PLOS’s NAAS tool - https://ngplosjournals.pagemajik.ai/artanalysis, NAAS will process the files provided and display the results in the "Uploaded Files" section of the page as the processing is complete. If the uploaded figures meet our requirements (or NAAS is able to fix the files to meet our requirements), the figure will be marked as "fixed" above. If NAAS is unable to fix the files, a red "failed" label will appear above. When NAAS has confirmed that the figure files meet our requirements, please download the file via the download option, and include these NAAS processed figure files when submitting your revised manuscript.

**Reproducibility:**

To enhance the reproducibility of your results, we recommend that authors of applicable studies deposit laboratory protocols in protocols.io, where a protocol can be assigned its own identifier (DOI) such that it can be cited independently in the future. Additionally, PLOS ONE offers an option to publish peer-reviewed clinical study protocols. Read more information on sharing protocols at https://plos.org/protocols?utm_medium=editorial-email&utm_source=authorletters&utm_campaign=protocols

# Author comment: Author response to Decision Letter 1

**DOI:** 10.1371/journal.pntd.0013651.r002

18 Mar 2026

Attachment Submitted filename: Response_to_reviewers.docx

# Peer review: Decision Letter 1

**DOI:** 10.1371/journal.pntd.0013651.r003

**Authors:** Stuart Blacksell, Vasantha Neela

20 Apr 2026

Dear Ms. Edwards,

We are pleased to inform you that your manuscript 'Efficacy and safety outcomes reported in human leptospirosis studies to inform the development of a core outcome and core outcome measurement set: a systematic review' has been provisionally accepted for publication in PLOS Neglected Tropical Diseases.

Before your manuscript can be formally accepted you will need to complete some formatting changes, which you will receive in a follow up email. A member of our team will be in touch with a set of requests.

Please note that your manuscript will not be scheduled for publication until you have made the required changes, so a swift response is appreciated.

IMPORTANT: The editorial review process is now complete. PLOS will only permit corrections to spelling, formatting or significant scientific errors from this point onwards. Requests for major changes, or any which affect the scientific understanding of your work, will cause delays to the publication date of your manuscript.

Should you, your institution's press office or the journal office choose to press release your paper, you will automatically be opted out of early publication. We ask that you notify us now if you or your institution is planning to press release the article. All press must be co-ordinated with PLOS.

Thank you again for supporting Open Access publishing; we are looking forward to publishing your work in PLOS Neglected Tropical Diseases.

Best regards,

Vasantha Kumari Neela

Academic Editor

PLOS Neglected Tropical Diseases

Stuart Blacksell

Section Editor

PLOS Neglected Tropical Diseases

Shaden Kamhawi

co-Editor-in-Chief

PLOS Neglected Tropical Diseases

orcid.org/0000-0003-4304-636XX

Paul Brindley

co-Editor-in-Chief

PLOS Neglected Tropical Diseases

orcid.org/0000-0003-1765-0002

***********************************************************

p.p1 {margin: 0.0px 0.0px 0.0px 0.0px; line-height: 16.0px; font: 14.0px Arial; color: #323333; -webkit-text-stroke: #323333}span.s1 {font-kerning: none

Reviewer's Responses to Questions

**Key Review Criteria Required for Acceptance?**

As you describe the new analyses required for acceptance, please consider the following:

**Methods**

-Are the objectives of the study clearly articulated with a clear testable hypothesis stated?

-Is the study design appropriate to address the stated objectives?

-Is the population clearly described and appropriate for the hypothesis being tested?

-Is the sample size sufficient to ensure adequate power to address the hypothesis being tested?

-Were correct statistical analysis used to support conclusions?

-Are there concerns about ethical or regulatory requirements being met?

Reviewer #3: Strengths:

The methodology is rigorous and transparent, following PRISMA and COMET guidelines.

The inclusion of multiple languages and global databases ensures broad representation.

The use of QualSyst and ORBIT tools for quality and bias assessment is appropriate.

Suggestions:

Justify the inclusion of descriptive studies (case reports/series) more explicitly. While acknowledged in the discussion, a brief rationale in the methods would strengthen the approach.

Clarify how outcome grouping and COMET domain mapping were validated (e.g., consensus among authors, steering committee review).

Consider adding a flowchart or table summarizing the data extraction process (e.g., variables extracted, tools used).

**********

**Results**

-Does the analysis presented match the analysis plan?

-Are the results clearly and completely presented?

-Are the figures (Tables, Images) of sufficient quality for clarity?

Reviewer #3: Strengths:

The results are comprehensive and well-organized, with clear thematic grouping of outcomes and measures.

The use of sunburst charts and treemaps effectively visualizes the heterogeneity in outcomes and treatments.

The global distribution of studies (63 countries) and temporal trends (e.g., shift in antibiotic use) are insightful.

Suggestions:

Highlight key gaps in the results section (e.g., underreporting of patient-centered outcomes, lack of standardized measures).

Discuss the implications of regional variations (e.g., why cephalosporins are more common in recent studies from South Asia).

**********

**Conclusions**

-Are the conclusions supported by the data presented?

-Are the limitations of analysis clearly described?

-Do the authors discuss how these data can be helpful to advance our understanding of the topic under study?

-Is public health relevance addressed?

Reviewer #3: Strengths:

The conclusion is concise and actionable, reinforcing the need for a COS and patient-centered outcomes.

The call for qualitative research and standardized measures is well justified.

Suggestions:

End with a strong, forward-looking statement (e.g., "This review lays the foundation for a COS that could transform leptospirosis research and patient care globally").

**********

**Editorial and Data Presentation Modifications?**

Use this section for editorial suggestions as well as relatively minor modifications of existing data that would enhance clarity. If the only modifications needed are minor and/or editorial, you may wish to recommend “Minor Revision” or “Accept”.

Reviewer #3: None

**********

**Summary and General Comments**

Use this section to provide overall comments, discuss strengths/weaknesses of the study, novelty, significance, general execution and scholarship. You may also include additional comments for the author, including concerns about dual publication, research ethics, or publication ethics. If requesting major revision, please articulate the new experiments that are needed.

Reviewer #3: Title and Abstract

Strengths:

The title is clear, concise, and accurately reflects the scope of the review.

The abstract effectively summarizes the background, methods, results, and conclusions, adhering to the journal’s requirements.

Suggestions:

Consider emphasizing the global health burden of leptospirosis in the abstract to underscore its relevance.

Clarify the novelty of the review (e.g., "first comprehensive synthesis of outcomes and measures in leptospirosis research").

Introduction

Strengths:

The introduction provides a compelling rationale for the study, highlighting the gap in standardized outcome reporting and its impact on clinical trials.

The global burden of leptospirosis is well contextualized with recent data (e.g., 1.03 million cases/year, USD 29.3 billion productivity loss).

The link between heterogeneous outcome reporting and weak evidence for treatments is clearly articulated.

Suggestions:

Briefly mention why a core outcome set (COS) is urgently needed for leptospirosis, beyond just improving trial quality (e.g., guiding policy, resource allocation).

Add a sentence on the potential impact of a COS on patient care and public health.

Methods

Strengths:

The methodology is rigorous and transparent, following PRISMA and COMET guidelines.

The inclusion of multiple languages and global databases ensures broad representation.

The use of QualSyst and ORBIT tools for quality and bias assessment is appropriate.

Suggestions:

Justify the inclusion of descriptive studies (case reports/series) more explicitly. While acknowledged in the discussion, a brief rationale in the methods would strengthen the approach.

Clarify how outcome grouping and COMET domain mapping were validated (e.g., consensus among authors, steering committee review).

Consider adding a flowchart or table summarizing the data extraction process (e.g., variables extracted, tools used).

Results

Strengths:

The results are comprehensive and well-organized, with clear thematic grouping of outcomes and measures.

The use of sunburst charts and treemaps effectively visualizes the heterogeneity in outcomes and treatments.

The global distribution of studies (63 countries) and temporal trends (e.g., shift in antibiotic use) are insightful.

Suggestions:

Highlight key gaps in the results section (e.g., underreporting of patient-centered outcomes, lack of standardized measures).

Discuss the implications of regional variations (e.g., why cephalosporins are more common in recent studies from South Asia).

Discussion

Strengths:

The discussion critically addresses the heterogeneity in outcome reporting and its implications for evidence synthesis.

The limitations are honestly acknowledged, including the influence of descriptive studies and potential reviewer bias.

The need for patient-reported outcomes and qualitative research is strongly emphasized.

Suggestions:

Strengthen the policy implications: How could a COS influence WHO guidelines, antimicrobial stewardship, or resource allocation?

Address the feasibility of implementing a COS in low-resource settings (e.g., cost, training needs).

Compare your findings with other neglected tropical diseases (e.g., dengue, melioidosis) to highlight unique challenges in leptospirosis.

Conclusion

Strengths:

The conclusion is concise and actionable, reinforcing the need for a COS and patient-centered outcomes.

The call for qualitative research and standardized measures is well justified.

Suggestions:

End with a strong, forward-looking statement (e.g., "This review lays the foundation for a COS that could transform leptospirosis research and patient care globally").

Minor Edits and Clarifications

Abstract: Replace "weak due to poor trial design" with "limited by heterogeneous outcome reporting and trial design."

Introduction: Clarify the timeline of the COS-LEP project (e.g., "This review is Phase 1 of the COS-LEP project").

Methods: Specify if machine translation was used for non-English studies and its potential limitations.

Results: Define abbreviations (e.g., MAT, ELISA) at first use.

Discussion: Avoid overstating the lack of AMR evidence—acknowledge the methodological challenges in testing susceptibility.

Overall Assessment:

This is a high-quality, timely systematic review that fills a critical gap in leptospirosis research. With minor revisions to sharpen the narrative and visuals, it will be a valuable resource for researchers, clinicians, and policymakers. The manuscript is well positioned for acceptance in PLOS Neglected Tropical Diseases.

**********

PLOS authors have the option to publish the peer review history of their article (what does this mean?). If published, this will include your full peer review and any attached files.

If you choose “no”, your identity will remain anonymous but your review may still be made public.

**Do you want your identity to be public for this peer review?**  For information about this choice, including consent withdrawal, please see our Privacy Policy.

Reviewer #3: No

# Editor report: Acceptance letter

**DOI:** 10.1371/journal.pntd.0013651.r004

**Authors:** Stuart Blacksell, Vasantha Neela

Dear Ms. Edwards,

We are delighted to inform you that your manuscript, "Efficacy and safety outcomes reported in human leptospirosis studies to inform the development of a core outcome and core outcome measurement set: a systematic review," has been formally accepted for publication in PLOS Neglected Tropical Diseases.

We have now passed your article onto the PLOS Production Department who will complete the rest of the publication process. All authors will receive a confirmation email upon publication.

The corresponding author will soon be receiving a typeset proof for review, to ensure errors have not been introduced during production. Please review the PDF proof of your manuscript carefully, as this is the last chance to correct any scientific or type-setting errors. Please note that major changes, or those which affect the scientific understanding of the work, will likely cause delays to the publication date of your manuscript. Note: Proofs for Front Matter articles (Editorial, Viewpoint, Symposium, Review, etc...) are generated on a different schedule and may not be made available as quickly.

Soon after your final files are uploaded, the early version of your manuscript will be published online unless you opted out of this process. The date of the early version will be your article's publication date. The final article will be published to the same URL, and all versions of the paper will be accessible to readers.

For Research Articles, you will receive an invoice from PLOS for your publication fee after your manuscript has reached the completed accept phase. If you receive an email requesting payment before acceptance or for any other service, this may be a phishing scheme. Learn how to identify phishing emails and protect your accounts at https://explore.plos.org/phishing.

Thank you again for supporting open-access publishing; we are looking forward to publishing your work in PLOS Neglected Tropical Diseases.

Best regards,

Shaden Kamhawi

co-Editor-in-Chief

PLOS Neglected Tropical Diseases

Paul Brindley

co-Editor-in-Chief

PLOS Neglected Tropical Diseases
